# Active generation of nanoholes in DNA origami scaffolds for programmed catalysis in nanocavities

**DOI:** 10.1038/s41467-019-12933-9

**Published:** 2019-10-31

**Authors:** Jianbang Wang, Liang Yue, Ziyuan Li, Junji Zhang, He Tian, Itamar Willner

**Affiliations:** 1 Institute of Chemistry, Center for Nanoscience and Nanotechnology, The Hebrew University of Jerusalem, Jerusalem, 91904 Israel; 2 Key Laboratory for Advanced Materials, School of Chemistry and Molecular Engineering, East China University of Science and Technology, Shanghai, China

**Keywords:** Supramolecular chemistry, DNA nanotechnology, DNA nanostructures

## Abstract

DNA origami tiles provide nanostructures for the spatial and temporal control of functional loads on the scaffolds. Here we introduce the active generation of nanoholes in the origami scaffolds using DNAzymes or light as triggers and present the programmed and switchable catalysis in the resulting nanocavities. We engineer “window” domains locked into the origami scaffolds by substrates of the Zn^2+^-ion- or Pb^2+^-ion-dependent DNAzymes. Using Zn^2+^ ions and/or Pb^2+^ ions, the programmed unlocking of the “window” domains is demonstrated. The tailored functionalization of the origami scaffolds allows the programmed operation of catalytic processes in the confined nanocavities. Also, the “window” domain is integrated into the origami scaffold using photoisomerizable azobenzene-modified locks. The cyclic photoisomerization of the locks between the cis and trans states leads to a reversible opening and closure of the nanoholes and to the cyclic light-induced switching of catalytic processes in the nanocavities.

## Abstract (web summary)

DNA origami provide scaffolds for spatial and temporal control of catalytic functions in confined nanocavities. Here the authors engineer nanoholes in DNA origami scaffolds.

## Introduction

The programmed assembly of two-dimensional (2D) and three-dimensional (3D) DNA origami nanostructures represents a major advance in DNA nanotechnology^1–3^. Besides ingenious shapes of origami structures generated by the programmed folding of the long-chain M13 phage DNA with dictated “staple” strands, origami structures were functionalized with protruding nucleic acid tethers or edge-modified oligonucleotide strands. The protruding strands were used as anchoring sites for the organization of polymers^4,5^, proteins^6^, and nanoparticles^7–9^ on the origami scaffolds. Unique functions of the nanostructures assembled on the origami scaffolds were demonstrated, such as the operation of enzyme cascades^10,11^, the design of plasmonic antennas^12,13^, and the assembly of chiroplasmonic structures^14,15^. In addition, dynamic processes of the constituents linked to the origami structures were demonstrated^16^. These included, e.g., the design of DNA walkers^17–19^, electric field operation of a robotic arm^20^, and the signal-triggered translocation of chiroplasmonic nanostructures^14,21–23^. The edge functionalization of origami tiles was applied to design programmed multi-component origami structures^24^ and particularly to develop switchable origami dimers^25–27^. For example, the reversible pH-driven formation of edge-confined, i-motif, or triplex nucleic acid functionalities were applied to stimulate the reversible dimerization and separation of origami tiles. In addition, pH or light was used to induce the reversible isomerization of linear/bent origami nanostructures^25,28^. In addition, the programmed reversible exchange of the compositions of the pairs of origami tiles using the K^+^-ion-induced formation of G-quadruplexes and their separation, in the presence of crown ether, was demonstrated^29^. Besides 2D DNA origami nanostructures, ingenious 3D origami systems were fabricated. For example, the self-assembly of an origami box^30^, the stepwise assembly of gigadalton-scale programmable DNA structures^31^, and the light-driven motion of 3D origami bundles to yield reversible chiroptical functions^21,32^ have been demonstrated. Different applications of origami nanostructures were suggested, including programmed catalysis^33^, controlled drug-release^34,35^, logic gate operations^36,37^, and sensing^38^.

Most of these functional origami structures involved, however, the bottom-up modification of the origami rafts, the edge modification of origami tiles, or the folding of the tiles into tubes. One may, however, consider the functionalization of origami structures with nanocavities (holes or barrels) that might act as containments or channels for guided chemical transformations. To date, such cavities have been fabricated within the passive assembly of the origami tiles^39,40^ and these cavities were used for the site-specific docking of antibodies^41^, the reconstitution of membrane proteins^42^, and the functionalization of solid-state pores for selective transport^40,43^. In addition, DNA structures (not origami) have been introduced into membranes and these acted as channels for the potential-stimulated transport of charges species across the membranes^44,45^. In contrast, the present study introduces the concept of “active” fabrication of nanoholes in origami tiles. We report on the DNAzyme-guided active formation of nanoholes in the origami scaffolds and the molecular “mechanical” unlocking of the nanoholes by lifting the covered “window” domains. By applying two different DNAzymes, the programmed and triggered fabrication of nanoholes in the origami structures is demonstrated. We further utilize the cavities in the different origami scaffolds as confined nano-environments for selective and specific catalysis. In addition, we highlight a design for the reversible light-driven mechanical opening and closure of the nanoholes, and the switchable catalysis in the nanocavities.

## Results

### Active and programmed generation of nanoholes by DNAzymes

**Fig. 1:**
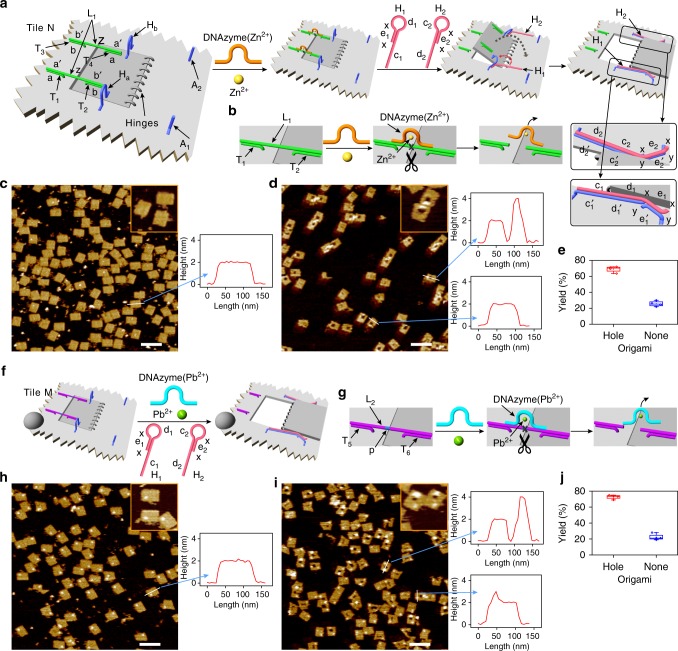
DNAzyme-driven formation of nanoholes in origami tiles. **a** Schematic mechanistic formation of the nanohole in the origami tile N using the Zn^2+^-ion-dependent DNAzyme as unlocking catalyst. The origami tile includes a locked “window” domain linked to the origami scaffold by eight hinges, and is modified by two “handles” and two “anchor” tethers. Note that the scheme only shows the key part of the origami tile. **b** Schematic Zn^2+^-dependent DNAzyme-driven unlocking of the “window” by the cleavage of the substrate using the hairpin-assisted opening of the “window” through hybridization to the handles and its pulling to the anchoring sites. **c** AFM image corresponding to the locked configuration of origami tiles. **d** AFM image corresponding to the unlocked origami tiles that include the nanoholes. Insets: enlarged structures of the respective tiles. The AFM images are accompanied by respective cross-section analyses of the tiles confirming the active formation of the nanoholes. Source data are provided as a Source Data file. **e** Statistical analysis of the nanohole-functionalized tiles generated by the Zn^2+^-ion-dependent DNAzyme (for details, see Supplementary Table 1). **f** Schematic mechanistic formation of the nanohole in the origami tile M using the Pb^2+^-ion-dependent DNAzyme as unlocking catalyst. The origami tile is labeled with a four-hairpin label (marked with a hemispheroid) to identify the tile. **g** Schematic Pb^2+^-ion-dependent DNAzyme-driven unlocking of the “window” by the cleavage of the ribonucleobase-modified substrate, associated with the origami tile. **h** AFM image corresponding to the origami tiles prior to the unlocking process. **i** AFM image of the origami tiles after the Pb^2+^-ion-dependent DNAzyme-driven unlocking of the “window”. Insets correspond to enlarged structures of the respective tiles. Cross-section analyses confirm the structural features of the labeled nanohole-modified tiles. Source data are provided as a Source Data file. **j** Statistical analysis of the “nanohole”-functionalized tiles generated by the Pb^2+^-ion-dependent DNAzyme (for details, see Supplementary Table 4). Error bars indicate the SD from the analyses of four imaged areas (2 μm × 2 μm). Scale bars: 200 nm

Catalytic nucleic acids (DNAzymes) find broad interest in the area of DNA nanotechnology due to their versatile applications for sensing^46–49^, operation of nanodevices^19,50^, synthesis of smart materials, e.g., hydrogels^51^, and the development of stimuli-responsive drug carriers^52^. For example, the hemin associated with the K^+^-ion-stabilized G-quadruplex yields a horseradish peroxidase-mimicking DNAzyme^53^. In addition, specific nucleic acid sequences bind metal ions^54–56^, e.g., Mg^2+^, Zn^2+^, Pb^2+^, or organic ligands, e.g., histidine^57^, to yield supramolecular catalytic structures with nucleic acids as substrates, often ribonucleobase-modified nucleic acid substrates. The catalytic hydrolysis and cleavage of the substrates by these structures were demonstrated. Figure 1a outlines the principles to stimulate the active DNAzyme-driven formation of a nanohole in an origami scaffold. The origami tile N (left in Fig. 1a) includes a domain acting as a “window” to be opened upon the DNAzyme-driven formation of the nanohole in the scaffold. The “window” domain is linked to the origami scaffold by means of eight hinges. In addition, the “window” domain is firmly locked into the origami frame by the crosslinking of the protruding strands T_1_/T_3_ and T_2_/T_4_, linked to the origami frame and the “window” by the hybridization of the strand L_1_ (complementary sequences “a/a′” and “b/b′”). The interbridging strand L_1_ includes the domain “z” that provides the substrate for the catalytically inactive Zn^2+^-ion-dependent DNAzyme sequence. In addition, two units (H_a_/H_b_) at opposite sides of the “window”, consisting of a single strand linked at its ends to the origami frame (one end) and the “window” raft (the other end), are engineered and act as “handles” assisting the opening of the “window”. Furthermore, two protruding anchor strands, A_1_ and A_2_, “y-e_1_′-y” and “y-e_2_′-y”, respectively, are engineered onto the origami tile for stretching the “window” into the open position (vide infra) (Fig. 1a and Supplementary Fig. 1). In the presence of Zn^2+^ ions and the helper hairpins H_1_/H_2_, the “window” domain is opened actively by the following mechanism: the Zn^2+^-ion-dependent DNAzyme^55,58^ cleaves the two substrate domains “z” associated with the locks (Fig. 1b). The hairpins H_1_/H_2_ added to the origami system include the domains “x-e_1_-x-d_1_-c_1_”/“d_2_-c_2_-x-e_2_-x” that hybridize with the handles H_a_/H_b_ placed on opposite sides of the “window.” It leads to the opening of H_1_ and H_2_ to yield the “toehold” tethers “x-e_1_-x” and “x-e_2_-x,” which form duplexes “e_1_/e_1_′” and “e_2_/e_2_′” with the protruding anchors A_1_ and A_2_, respectively. This process provides the hybridization-guided mechanical stretching and opening of the “window” on the origami raft and the generation of the nanohole (right in Fig. 1a). Figure 1c, d show the atomic force microscopy (AFM) images of the origami tiles before (Fig. 1c) and after (Fig. 1d) the addition of Zn^2+^ ions and the helper hairpins H_1_/H_2_. Before the addition of Zn^2+^ ions and H_1_/H_2_, intact origami tiles are observed (Fig. 1c). The addition of Zn^2+^ ions and H_1_/H_2_ leads to the formation of nanoholes in the origami tiles (Fig. 1d). The cross-section analysis of the origami tiles confirms the Zn^2+^ ions/H_1_/H_2_-guided unlocking of the “windows” and the formation of the nanoholes. Although the intact origami tiles show a height of ca. 2 nm and length of ca. 100 nm, the interaction with Zn^2+^ ions/H_1_/H_2_ yields a domain ca. 50 nm long and 2 nm high, followed by a vacant domain (20–25 nm long) and a high domain (ca. 4 nm high) that corresponds to the open origami “window” laying down on the origami raft (double height of the origami-bright domain on the AFM images). Statistical analyses of four 2 μm × 2 μm scanned areas reveal that ca. 70% of the origami tiles include the nanoholes, where ca. 30% are either in the locked configuration or not well-defined structures (Fig. 1e, Supplementary Fig. 2 and Supplementary Table 1). It should be noted that both components of the Zn^2+^ ions and H_1_/H_2_ are essential to unlock the “window” and to generate the nanoholes. In the presence of Zn^2+^ ions only, a low yield (ca. 8%) of open nanoholes are observed, and in the presence of H_1_/H_2_, only no nanohole-containing origami structures are detected (Supplementary Figs. 3 and 4, and Supplementary Tables 2 and 3; in addition, Supplementary Fig. 5 provides a statistical analysis of the results according to Student’s *T*-test). Further support for the DNAzyme-triggered unlocking of the “window” domain, and the subsequent “helpers” induced opening of the “window” and its fixation on the anchoring sites, was obtained by complementary fluorescence resonance energy transfer (FRET) experiments. In these experiments, the handle H_a_ was internally modified with Cy3, and the anchoring foothold was modified with Cy5. The FRET signal (Cy3 to Cy5) generated upon the “helper”-induced opening of the window, and the use of an appropriate calibration curve allowed us to quantify the yield of “open window” structures (for a detailed discussion of the FRET experiments, extraction of the calibration curve, the FRET results, and the quantification of the “open window” origami structures, see Supplementary Fig. 6 and accompanying discussion). The results reveal that the yield of “open window” in origami structure is ca. 70%, in agreement with the statistical analysis of AFM images.

Similarly, the active unlocking of the “window” domain associated with the origami scaffold M using the Pb^2+^-ion-dependent DNAzyme^56^ as the unlocking catalyst was demonstrated (Fig. 1f). In this system, the lock strand L_2_ is used to crosslink the “window” domain to the origami scaffold (Fig. 1f, g). The strand L_2_ includes the ribonucleotide-containing domain “p” that acts as the substrate of the Pb^2+^-ion-dependent DNAzyme. The tile M is modified with the eight hinges, the two handle units, and the protruding tethers for anchoring of the open “window” through hybridization of the helpers/handles/anchors (Supplementary Fig. 7). The Pb^2+^-ion-dependent DNAzyme sequence is hybridized, in a catalytically inactive configuration, with the substrate sequence “p”. In the presence of Pb^2+^ ions and H_1_/H_2_, the substrate domains are cleaved (Fig. 1g), leading to the active unlocking of the “window” and to the opening of the nanohole by the “handle”-stimulated opening of H_1_/H_2_ and the “mechanical” stretching of the “window” on the origami raft by the hybridization of the single-stranded toehold associated with the open hairpins with anchoring sites A_1_/A_2_. It is noteworthy that the origami tile unlocked by the Pb^2+^-ion-dependent DNAzyme is labeled with a four-hairpin label (to distinguish between the origami tiles being unlocked by Zn^2+^ ions and/or Pb^2+^ ions, vide infra). Figure 1h, i show the AFM images corresponding to the intact locked origami tiles and the unlocked origami tiles, respectively. The label (bright spot) is observed on all tiles. Subjecting the tiles to Pb^2+^ ions and H_1_/H_2_ unlocks the “window” domains leading to the opening of the nanoholes (inset shows the enlarged image of the resulting tiles). In addition to the hole and the label, the folding of the “window” across the “hinges” and the lay-down of the “window” on the origami raft opposite to the locking domain are clearly visible (brighter and higher domain). The cross-section analysis of the respective tiles shows the intact locked tiles with the characteristics ca. 100 nm length and ca. 2 nm height. A spike ca. 1 nm high corresponding to the label is observed on the background height of the origami frame. The unlocked tiles show the background height of the origami scaffold, 2 nm, a void domain that confirms the formation of the nanoholes, and a domain of double height, ca. 4 nm, consistent with the lay-down of the stretched “window” on the origami. Figure 1j presents the statistical analysis of the contents of the unlocked nanohole-origami tiles (four 2 μm × 2 μm scanned areas; Supplementary Fig. 8 and Supplementary Table 4), where ca. 72% of the origami tiles include the nanoholes. As before, control experiments imply that the activation of the Pb^2+^-ion-dependent DNAzyme and the addition of H_1_/H_2_ are essential to generate the high yield of nanoholes (see Supplementary Figs. 9 and 10, and Supplementary Tables 5 and 6, and accompanying discussion). These results indicate the high-yield formation of nanohole-containing origami structures upon the Pb^2+^-ion-dependent DNAzyme cleavage of the locks and the concomitant “mechanical” stretching of the “window” on the origami rafts by means of the handles/helper hairpins/anchoring footholds (H_a_/H_1_/A_1_ and H_b_/H_2_/A_2_).

**Fig. 2:**
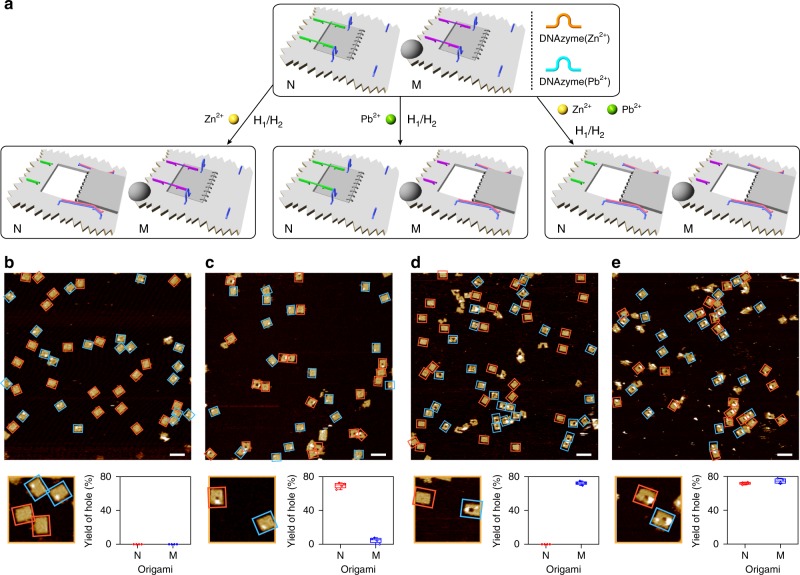
Programmed and selective formation of nanoholes in the mixture of origami tiles N and M, dictated by the two DNAzymes. **a** Top, schematic mixture of the origami tiles N and M. Bottom-left, selective unlocking and formation of nanoholes in tiles N by activating the Zn^2+^-ion-dependent DNAzyme only. Bottom-middle, selective unlocking and formation of nanoholes in origami tiles M by activating the Pb^2+^-ion-dependent DNAzyme only. Bottom-right, the concomitant formation of nanoholes in the mixture of origami tiles N and M upon activation of the two Zn^2+^-ion- and Pb^2+^-ion-dependent DNAzymes. **b** AFM image corresponding to the mixture of locked origami tiles N and M. **c** AFM image of the origami tile mixture generated upon the activation of the Zn^2+^-ion-dependent DNAzyme (Cf. bottom-left in **a**). **d** AFM image corresponding to the origami tile mixture generated upon the activation of the Pb^2+^-ion-dependent DNAzyme (Cf. bottom-middle in **a**). **e** AFM image of the origami tile mixture generated upon the activations of Zn^2+^-ion- and Pb^2+^-ion-dependent DNAzymes (Cf. bottom-right in **a**). Each of the AFM images is accompanied by a statistical analysis of the origami constituents N and M in the respective systems (for details, see Supplementary Tables 7–9). Error bars indicate the SD from the analyses of four imaged areas (2 μm × 2 μm). Scale bars: 200 nm

The programmed unlocking of the locked origami tiles, in the presence of Zn^2+^ ions and/or Pb^2+^ ions, was then evaluated (Fig. 2). In the presence of Zn^2+^ ions and the helper hairpins, only the origami tiles lacking the labels, N, are unlocked to yield the nanoholes. In the presence of Pb^2+^ ions and the helper hairpins, only the labeled origami tiles, M, are unlocked to yield the nanoholes, and in the presence of Zn^2+^ ions, Pb^2+^ ions and the helper hairpins, the guided formation of the nanoholes in the two origami tiles proceeds (Fig. 2a). Figure 2b-e show the AFM images of the respective systems and the statistical analysis of the contents of the tiles in the different systems. Figure 2b shows the AFM image of the intact origami tiles N and M, and Fig. 2c depicts the AFM image of the origami mixture after treatment with Zn^2+^ ions and H_1_/H_2_. The unlabeled tiles N reveal a high content of nanohole-modified rafts, whereas no nanoholes are observed in the labeled origami tiles M. The statistical analysis of the origami tiles indicates ca. 70% of hole-containing rafts and ca. 30% of closed or non-defined structures of the unlabeled tiles N. The content of the closed tiles M is ca. 100% (Supplementary Fig. 11 and Supplementary Table 7). Figure 2d shows the AFM image of the origami mixture treated with Pb^2+^ ions and H_1_/H_2_. The unlabeled tiles N stay closed (100%), whereas the labeled tiles M include nanoholes (ca. 72%) and a content of ca. 28% origami rafts that stay locked or show a non-defined structure (respective statistical evaluation of the structures; Supplementary Fig. 12 and Supplementary Table 8). Upon treatment of the origami mixture with Zn^2+^ ions, Pb^2+^ ions, and H_1_/H_2_, both tiles, N and M, reveal nanohole-containing structures with yields corresponding to ca. 72% and ca. 75%, respectively (Fig. 2e, Supplementary Fig. 13, and Supplementary Table 9). It should be noted that the hairpin marker in tile M is distinguishable from the “open-window” domain associated with tile N by the height characterizing the hairpin marker (3 nm) and the height of a double-layer origami structure of the “open window” domain (4 nm).

### Programmed catalysis in DNAzyme-drilled nanocavities

**Fig. 3:**
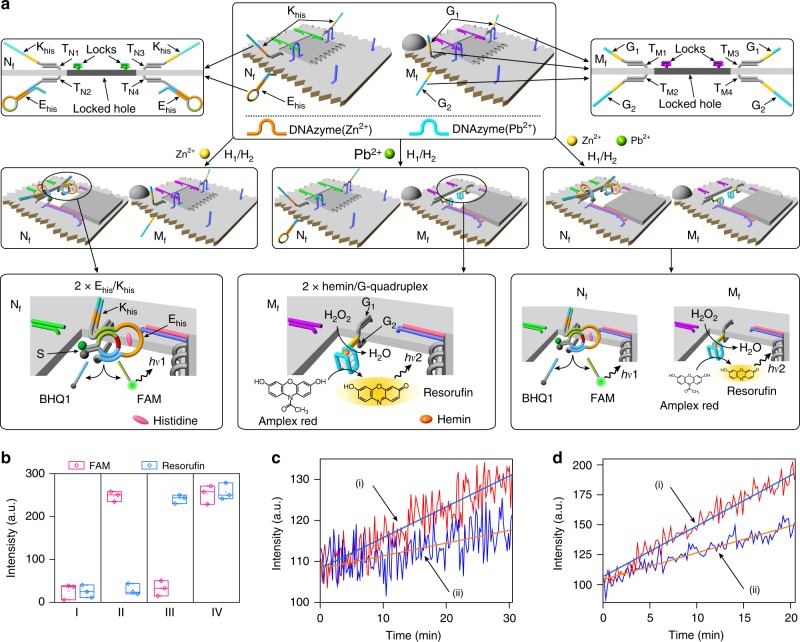
Programmed catalytic transformation in confined cavities in DNA origami scaffolds. **a** The mixture of two origami tiles N_f_ and M_f_, which include the locking/unlocking components for the opening of respective “window” domains by the Zn^2+^-ion- and/or Pb^2+^-ion-dependent DNAzymes (Top). Middle-left, treatment of the origami mixture with Zn^2+^ ions unlocks the “window” associated with tile N_f_ and its opened “window” allows the K_his_ strand-induced uncaging of the E_his_ and the activation of the histidine-dependent DNAzyme in the resulting cavity. Middle-center, subjecting the origami mixture to Pb^2+^ ions leads to the unlocking of the “window” of tile M_f_. The opened “window” in tile M_f_ allows the interaction of the subunits G_1_ and G_2_, and the assembly of the hemin/G-quadruplex DNAzyme in the resulting cavity. Middle-right, treatment of the origami mixture with Zn^2+^ ions and Pb^2+^ ions leads to the unlocking of the two “windows” associated with N_f_ and M_f_. The opened “windows” in both tiles allow the activation of the respective catalytic transformations. **b** Fluorescence outputs (FAM and/or resorufin) generated by: Entry I, the locked mixture of N_f_ and M_f_. Entry II, the treatment of the origami mixture with Zn^2+^ ions and the activation of the histidine-dependent DNAzyme in the confined cavity of N_f_. Entry III, the treatment of the origami mixture with Pb^2+^ ions and the activation of the hemin/G-quadruplex DNAzyme in the confined cavity of M_f_. Entry IV, treatment of the origami mixture with Zn^2+^ ions and Pb^2+^ ions. Error bars indicate the SD from three independent experiments (for details, see Supplementary Fig. 18). **c** The catalytic activities of the histidine-dependent DNAzymes in: (i) the confined cavities in the origami tiles N_f_ and (ii) the homogenous identical buffer solution. The concentration of the histidine-dependent DNAzymes in the cavity or bulk solution is identical, 24.6 nM. Source data are provided as a Source Data file. **d** The catalytic activities of the hemin/G-quadruple DNAzymes in: (i) the confined cavities in the origami tiles M_f_ and (ii) the homogenous identical buffer solution. The concentration of the hemin/G-quadruple DNAzymes in the cavity or bulk solution is identical, 17.9 nM. Source data are provided as a Source Data file

In the next step, the Zn^2+^ ions/Pb^2+^ ions programmed unlocking of the origami tiles were used to stimulate controlled catalysis in the confined nanocavities (Fig. 3). Towards this goal, the Zn^2+^-ion-responsive tile N is further engineered to generate the tile N_f_ (Top-middle and left enlargement of Fig. 3a and Supplementary Fig. 14). One side of the origami raft is modified with the strand K_his_ that hybridizes with the protruding strands T_N1_/T_N3_. The bottom side of the origami raft is functionalized with the hairpin E_his_ that is attached to the raft through hybridization with the protruding strands T_N2_/T_N4_. It is noteworthy that the origami raft domain is engineered with 2 × K_his_/E_his_ units. The hairpin E_his_ includes the histidine-dependent DNAzyme sequence in a caged, inactive, configuration. The locked structure of the origami tile prohibits inter-communication between K_his_ and E_his_. The Zn^2+^-ion-induced unlocking of the “window” allows the interaction between K_his_ and E_his_ in the resulting cavity (middle-left in Fig. 3a). The units K_his_ and E_his_ are predesigned to allow the K_his_-induced opening of the hairpin E_his_ to yield the uncaged histidine-dependent DNAzyme^57^ in the cavity. The binding of the histidine-dependent DNAzyme to the FAM/BHQ1-modified substrate S leads, in the presence of histidine, to the cleavage of S. The resulting fluorescence of the FAM-modified fragment provides the transduction signal for the catalytic process proceeding in the confined cavity of the Zn^2+^-ion-responsive tiles N_f_ (Supplementary Fig. 15). Similarly, top-middle and accompanying right enlargement of Fig. 3a depict the modification of the origami tile M to form tile M_f_ with an engineered cavity allowing the programmed activation of the hemin/G-quadruplex horseradish peroxidase-mimicking DNAzyme (Supplementary Fig. 16). Toward this end, the upper origami raft is functionalized with the guanosine-rich sequences G_1_ that are linked to the raft through hybridization with the protruding tethers T_M1_/T_M3_. The bottom surface of the origami is functionalized by the guanosine-rich sequences G_2_ that are linked to the raft through hybridization with the protruding tethers T_M2_/T_M4_. It is worth noting that the origami raft is modified with 2 × G_1_/G_2_ pairs. In the locked configuration, the strands G_1_ and G_2_ are separated by the origami raft and cannot interact. The Pb^2+^-ion-induced unlocking of the origami tile allows the inter-communication of G_1_ and G_2_ in the resulting cavity. In the presence of K^+^ ions and hemin, the two strands assemble into the K^+^-ion-stabilized hemin/G-quadruplex horseradish peroxidase-mimicking DNAzyme that catalyzes the H_2_O_2_ oxidation of Amplex Red to the fluorescent Resorufin product. The resulting fluorescence of Resorufin provides a transduction signal for the catalytic process proceeding in the confined cavity of the Pb^2+^-ion-responsive tiles M_f_ (middle-center of Fig. 3a and Supplementary Fig. 17). Figure 3b shows the programmed activation of the DNAzyme-catalyzed processes operating in the respective nanocavities of the origami tiles (Supplementary Fig. 18). In these experiments, the mixture of the tiles N_f_ and M_f_ is subjected to the respective ion triggers. The mixture in the absence of Zn^2+^ ions or Pb^2+^ ions does not show any fluorescence signals (entry I) consistent with the lack of formation of any active DNAzymes. In the presence of Zn^2+^ ions, only the fluorescence of FAM is observed, and no fluorescence of Resorufin is detected (entry II). This is consistent with the selective Zn^2+^-ion-stimulated activation of the histidine-dependent DNAzyme units associated with the tile N_f_. Similarly, subjecting the mixture to Pb^2+^ ions results in the selective unlocking of tile M_f_, accompanied by the generation of the fluorescence of Resorufin (entry III). Under these conditions, no fluorescence of FAM is observed, indicating that the tiles N_f_ stay in the locked, inactive, configuration. Treatment of the mixture with Zn^2+^ ions and Pb^2+^ ions leads to the unlocking of the two types of origami tiles, resulting in the fluorescence of FAM and Resorufin (entry IV) consistent with the activation of the two types of DNAzymes. An important issue to address involves, however, an assessment on the activities of the hemin/G-quadruplex and histidine-dependent DNAzyme in the confined reaction cavities in the origami tiles vs. the activities of these DNAzymes in a homogenous aqueous phase (under the same concentrations). In fact, previous studies demonstrated that biocatalyic cascades in confined environments reveal enhanced activities due to the spatial concentrations of the catalysts in the confined volumes^59–61^. Thus, the confinement of the different DNAzyme subunits could affect the activities of the DNAzymes. Knowing the concentrations of the active DNAzyme associated with the cavities, we compared their activities in the confined environments with the activities of the DNAzyme in a homogenous phase at the same concentrations (Figs. 3c, d). We find that the activities of the histidine-dependent DNAzyme and of the hemin/G-quadruplex horseradish peroxidase-mimicking DNAzyme in the confined cavities are ca. twofold higher as compared with their activities in the homogenous solution.

### Light-induced switchable unlocking and locking of nanoholes

**Fig. 4:**
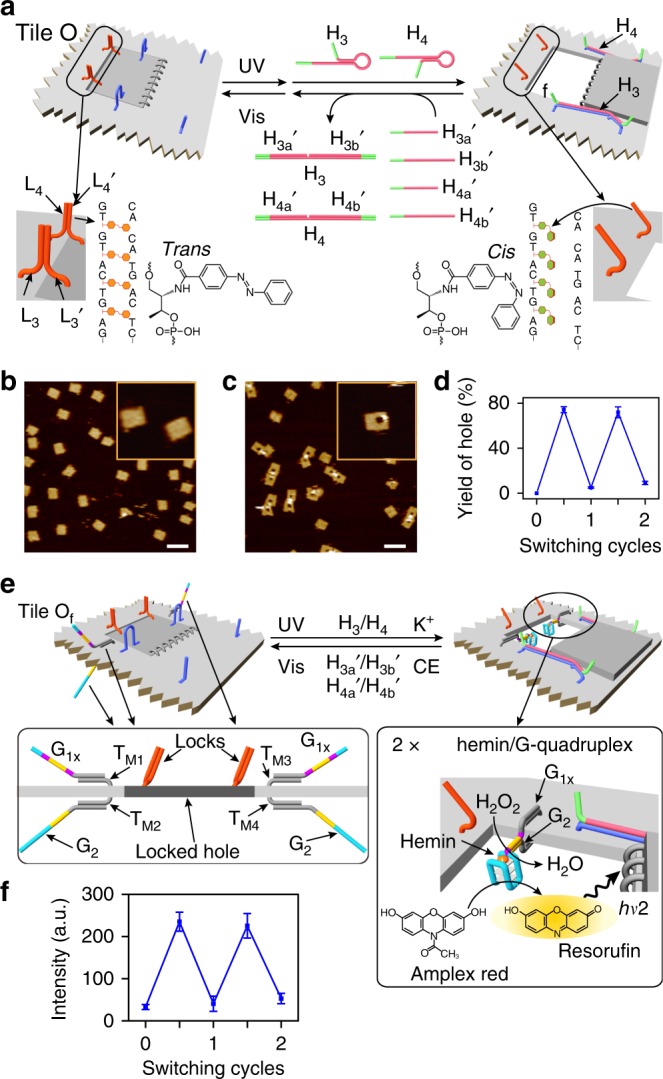
Switchable photoinduced unlocking and locking of the cavity in the origami tile. **a** Mechanism for the photoinduced unlocking and locking of the “window” domain in the origami tile O using trans-azobenzene stabilized locks. Photoisomerization of the trans-azobenzene units to cis-azobenzene separates the locks and the H_3_/H_4_ assisted mechanical opening of the nanoholes. Subjecting the “opened window” to the counter strands and the photoisomerization of the cis-azobenzene units to trans-azobenzene, recovers the locked configuration of tile O. **b** AFM image corresponding to the locked origami tiles. **c** AFM image of the “opened window” origami tiles generated upon photochemical unlocking of the tiles and concomitant treatment with H_3_/H_4_. The reverse locking of the tiles is achieved by irradiation of the “opened window” tiles and concomitant treatment of the tile with anti-helper strands. **d** Statistical analysis of the “open window” tile upon the cyclic irradiation of the origami tile in the presence of the respective helper hairpins and the anti-helper strands, respectively (for details, see Supplementary Tables 10–14). Error bars indicate the SD from the analyses of four imaged areas (2 μm × 2 μm). **e** Mechanism for the photoinduced reversible activation of the hemin/G-quadruplex DNAzyme in the photogenerated cavity associated with the tile O_f_. The photoinduced unlocking of the “window” and in the presence of hairpins H_3_/H_4_, and K^+^ ions and hemin lead to the formatioin of the hemin/G-quadruplex DNAzyme in the resulting cavity, resulting in catalyzed H_2_O_2_ oxidatioin of Amplex Red to the fluorescent Resorufin product. Irradiation of the “open window”/catalytic system in the presence of the anti-helper strands and 18-crown-6-ether results in the separation of the G-quadruplex catalytic units and the regeneration of the locked configuration of the tile. **f** Cyclic and reversible fluorescence changes generated by the origami tiles upon photoinduced unlocking or locking the tiles in the presence of the respective helpers/anti-helper strands and added K^+^ ions/crown ether. Error bars indicate the SD from three independent experiments (for details, see Supplementary Fig. 30). Scale bars: 200 nm

The DNAzyme-catalyzed formation of the nanoholes in the origami tiles and the resulting catalytic reactions in the confined cavities are, however, single-cycle processes and the switchable locking and unlocking of the nanoholes are practically prohibited. We, thus, searched for a locking/unlocking trigger that excludes the need to add nucleic acid strands. This is feasible by the application of light signals to reversibly open and close the nanoholes. This was achieved by applying photoisomerizable azobenzene-modified oligonucleotides as light-triggered units for the reversible unlocking and locking of the nanoholes as outlined in Fig. 4a. The “window” is engineered into the origami tile O by applying eight hinges and two handles, H_a_/H_b_, on opposite sides of the “window,” which link the “window” to the origami raft. The “window” is locked to the raft through the formation of two duplex locks between protruding strands L_3_/L_3_′ and L_4_/L_4_′. The locked duplexes are stabilized by trans-azobenzene units that intercalate in base pairs. In addition, the origami tile is modified with two protruding foothold tethers, A_1_/A_2_, which function as anchoring sites for stretching and fixing of the open “window” (Supplementary Fig. 19). Photoisomerization of the trans-azobenzene and in the presence of the helper hairpins, H_3_/H_4_, leads to the unlocking of the “window” and to its “mechanical” opening and stretching over the origami tile by the duplexes H_a_/H_3_/A_1_ and H_b_/H_4_/A_2_. Subjecting the hole-containing origami tiles to the anti-strands, H_3a_′/H_3b_′ and H_4a_′/H_4b_′, and the concomitant photoisomerization of the cis-azobenzene units to trans-azobenzene (*λ* > 420 nm) result in the displacement of the stretching strands from the handle units, H_a_/H_b_, and the anchoring tethers, A_1_/A_2_, leading to the formation of H_3_/H_3a_′/H_3b_′ and H_4_/H_4a_′/H_4b_′ as wastes and to the reclosure of the “flexible window” into the locked structure. By the reversible photochemical trans ⇔ cis isomerization of the azobenzene units in the presence of the helper and counter-helper strands, the nanoholes are cycled between open and closed states, respectively. Figure 4b, c show the AFM images of the intact closed tiles and the photogenerated nanohole-containing tiles (*λ* = 365 nm) in the presence of the helper hairpins, respectively. Figure 4d shows the cyclic opening and closure of the nanoholes upon the photoisomerization of the azobenzene units in the presence of the helper and counter-helper strands. The yield of nanohole-containing tiles is ca. 74% (Supplementary Figs 20–24 and Supplementary Tables 10–14). As before, the photoisomerization of the azobenzene units (*λ* = 365 nm) and the added hairpins H_3_/H_4_ are essential to generate the high yield of the stretched open “window” (see Supplementary Figs. 25–27 and Supplementary Tables 15 and 16, and accompanying discussion).

### Light-induced switchable catalysis in nanocavities

The light-induced opening and closure of the nanoholes were then applied to reversibly switch the hemin/G-quadruplex horseradish peroxidase-mimicking DNAzyme in the photogenerated nanocavities (Fig. 4e and Supplementary Fig. 28). Toward this goal, the upper and lower surfaces of the photo-responsive origami tiles are functionalized with guanosine-rich strands G_1×_ and G_2_, which hybridize with the protruding tethers T_M1_/T_M3_ and T_M2_/T_M4_, respectively. In the locked configuration, the strands G_1×_ and G_2_ are separated by the origami tile (left of Fig. 4e). The photochemical unlocking of the “window” and its opening in the presence of K^+^ ions and the helper hairpins result in the inter-communication of G_1×_ and G_2_ positioned on opposite sides of the origami raft and their assembly into the K^+^-ion-stabilized G-quadruplex in the nanocavity. It is noteworthy that the origami raft is modified with 2 × G_1×_/G_2_ pairs that yield two G-quadruplex units in the unlocked cavity. The binding of hemin to the G-quadruplex yields the hemin/G-quadruplex DNAzyme that catalyzes the H_2_O_2_ oxidation of Amplex Red to the fluorescent Resorufin product (right of Fig. 4e). The subsequent treatment of the catalytic origami nanostructure with 18-crown-6-ether (CE), the counter strands H_3a_′/H_3b_′ and H_4a_′/H_4b_′, and the illumination of the system with visible light results in the separation of the G-quadruplex DNAzyme (via elimination of the K^+^ ions, the removal of the stretching strands through the separation of the duplexes H_a_/H_3_/A_1_ and H_b_/H_4_/A_2_, and the locking of the nanohole through the regeneration of the trans-azobenzene-stabilized duplex locks. That is, the reversible treatment of the origami tiles with UV/strands/K^+^ ions and visible light/counter strands/crown ether (CE) leads to the cyclic “ON”/“OFF” switching of the hemin/G-quadruplex DNAzyme (Supplementary Fig. 29). Figure 4f demonstrates that the photo-responsive origami tiles indeed activate the switchable “ON”/“OFF” catalytic activities of the DNAzyme by the sequential triggered “mechanical” opening and closure of the cavity in which the catalysts are formed and separated (Supplementary Fig. 30), respectively.

## Discussion

The present study has introduced a method for the active generation of nanoholes in origami tiles and the programmed operation of catalytic transformations in the resulting nanocavities. One approach has included the unlocking of the nanoholes in the origami tiles using metal-ion-dependent DNAzymes as unlocking biocatalysts. The programmed unlocking of a mixture of origami tiles by Zn^2+^-ion- and/or Pb^2+^-ion-dependent DNAzymes, and the subsequent programmed operation of catalytic transformations in the confined nanocavities have been demonstrated. A second method to reversibly unlock and lock the nanoholes by means of auxiliary light signals, and to switch “ON”/“OFF” catalytic functions in the nanocavities using photoisomerizable azobenzene-modified strands as locks were accomplished. Beyond the nanotechnological impact of this study to design nanoholes in origami tiles as confined cavities for programmed catalytic transformations, the results highlight further applications of these nanostructures. For example, by appropriate design of the active origami rafts, the programmed unlocking of different-sized nanohole patterns may be envisaged. Such nanohole arrays could be used for multiplexed sensing. In addition, the incorporation of different enzymes or plasmonic nanoparticles into the engineered nanocavities could provide versatile means to operate biocatalytic cascades or to assemble plasmonic devices.

## Methods

### Materials

Oligonucleotides were purchased from Integrated DNA Technologies and FAM/BHQ1-labeled oligonucleotide was purified by high-performance liquid chromatography. Single-stranded M13mp18 DNA was obtained from New England Biolabs. Freeze ‘N Squeeze DNA Gel Extraction spin columns were purchased from Bio-Rad. Amicon centrifugal filters (100 k, NMWL) were purchased from Merck Millipore Ltd. Other chemical reagents were purchased from Sigma-Aldrich.

### Preparation of DNA origami tiles and the hairpins

To prepare DNA origami tiles, single-stranded M13mp18 phage DNA (10 nM) and short staple strands (100 nM, unmodified staple strands and functional staple strands) (Supplementary Tables 17–24) were dissolved in the TAE buffer (Tris, 20 mM; acetic acid, 20 mM; EDTA, 1 mM; pH 8.0) with 12.5 mM Mg^2+^. The mixture was heated to 95 °C in a thermal cycler and then allowed to cool down to 20 °C at a rate of −0.6 °C min^−1^. The DNA origami tiles were purified using agarose electrophoresis (1%, 100 V, 1.5 h, at 0 °C) to remove the excess staple strands and were then extracted from the gel bands using Freeze ‘N Squeeze spin columns.

All the hairpin strands (10 μM) were annealed from 90 °C to 10 °C at a rate of −3 °C min^−1^ in the TAE buffer (Tris, 20 mM; acetic acid, 20 mM; EDTA, 1 mM; pH 8.0) with 6 mM Mg^2+^ and 5 mM Na^+^.

### Formation of nanoholes in the origami tiles N and M

The purified origami tile N (2 nM) including the Zn^2+^-ion-dependent DNAzyme sequence (20 nM) was added with the Zn^2+^ ions (5 mM) (10 mM Tris, 20 mM Mg^2+^, pH 7.0). The sample was kept at 30 °C for 10 min. Then the sample was centrifuged (100 k NMWL, 3000 × *g*, 10 min, three times) to remove the DNAzyme sequence and the Zn^2+^ ions and the buffer was changed to TAE buffer with 6 mM Mg^2+^ and 5 mM Na^+^. The sample was added with the helper hairpins H_1_/H_2_ (10 nM for each one) and was kept at 25 °C for 10 h.

For the control experiments on the tile N (2 nM), only Zn^2+^ ions (5 mM) or the helper hairpins H_1_/H_2_ (10 nM for each one) were used in the nanohole-forming processes.

For the FRET measurement, the origami tile N with the internally Cy3-modified handle H_a-C3_ and Cy5-modified anchoring foothold A_1-C5_ was prepared. After the unlocking of the “window” by the Zn^2+^-ion-dependent DNAzyme (Zn^2+^, 5 mM) and the centrifugation (100 k NMWL, 3000 × *g*, 10 min, three times), the fluorescence spectrum of the origami tile N (set at 20 nM) was measured (*λ*_ex_ = 532 nm). Then the helper strands H_1_ and H_2_ were added into the sample to fix the “window” to the anchoring sites. The fluorescence spectrum of the origami tile N in the open state (set at 20 nM) was measured (*λ*_ex_ = 532 nm). For the calibration curve, the mixtures of the strands H_a-C3_ (20 nM) and A_1-C5_ (20 nM) were subjected to H_1-C_ with variable concentrations (0, 5, 10, 15, and 20 nM) and their fluorescence spectra were measured (*λ*_ex_ = 532 nm).

To form a nanohole in origami tile M, the sample of the purified tile M (2 nM) including the Pb^2+^-ion-dependent DNAzyme sequence (20 nM) was added with Pb^2+^ ions (100 μM) (10 mM Tris, 20 mM Mg^2+^, pH 7.0). The sample was kept at 30 °C for 10 min and then was centrifuged (100 k NMWL, 3000 × *g*, 10 min, three times) to remove the DNAzyme sequence and the Pb^2+^ ions, and the buffer was changed to TAE buffer with 6 mM Mg^2+^ and 5 mM Na^+^. The sample was added with the helper hairpins H_1_/H_2_ (10 nM for each one) and was kept at 25 °C for 10 h.

For the control experiments on the tile M (2 nM), only Pb^2+^ ions (100 μM) or the helper hairpins H_1_/H_2_ (10 nM for each one) were used in the nanohole-forming processes.

For the selective formation of nanoholes in the origami mixture, the equal amount of purified origami tiles N and M (2 nM for each tile) were mixed with the Zn^2+^-ion-dependent DNAzyme sequence (20 nM) and the Pb^2+^-ion-dependent DNAzyme sequence (20 nM) (10 mM Tris, 20 mM Mg^2+^, pH 7.0). It was divided into four samples and were added with different ion triggers. One sample was the prior mixture without added triggers as a reference. The second sample was added with the trigger of Zn^2+^ ions (5 mM). The third sample was added with the trigger of Pb^2+^ ions (100 μM). The fourth sample was added with triggers of Zn^2+^ ions (5 mM) and Pb^2+^ ions (100 μM). The samples were kept at 30 °C for 10 min, and then the DNAzyme sequences and the metal ions were removed by centrifugation (100 k NMWL, 3000 × *g*, 10 min, three times) and the buffer solutions were changed to TAE buffer with 6 mM Mg^2+^ and 5 mM Na^+^. Then the helper hairpins H_1_/H_2_ (20 nM for each one) were added and the sample was kept at 25 °C for 10 h.

### Measurement of the programmed catalytic activities

For the measurements of the catalytic reactions in the origami mixture, four samples of the mixture of the origami tiles N_f_ and M_f_ (20 nM, 120 μL for each one) with the Zn^2+^-ion-dependent DNAzyme sequence (200 nM) and the Pb^2+^-ion-dependent DNAzyme sequence (200 nM) (10 mM Tris, 20 mM Mg^2+^, pH = 7) were prepared. Then the four samples were added with different components of Zn^2+^ ions (5 mM) and/or Pb^2+^ ions (100 μM) as triggers to unlock the “windows.” One sample was added with no trigger acting as a reference, the second sample was added with Zn^2+^ ions, the third sample was added with Pb^2+^ ions, and the fourth sample was added with both ions. All the samples were kept at 30 °C for 10 min. After removing the DNAzyme sequences and metal ions (100 k NMWL, 3000 × *g*, 10 min, three times), the helper hairpins H_1_/H_2_ (200 nM for each one) were added into the four samples, respectively, and the samples were kept at 25 °C for 10 h in TAE buffer with 6 mM Mg^2+^ and 5 mM Na^+^. Then each sample was divided into two parts with same volumes. One part was added with histidine (5 mM) and substrate S (1 μM). The concentration of each tile was set at 15 nM (50 μL) in TAE buffer (with 6 mM Mg^2+^, 5 mM Na^+^, and 200 mM K^+^). The catalytic reaction was taken at 30 °C for 6 h and the fluorescence spectrum of the product (FAM-labeled fragment) was measured using a Cary Eclipse Fluorescence Spectrophotometer (Varian, Inc.) (*λ*_ex_ = 495 nm). The other one was treated with hemin (30 nM), Amplex Red (100 μM) and H_2_O_2_ (5 mM), and the concentration of each tile was 15 nM (50 μL) in TAE buffer (with 6 mM Mg^2+^, 5 mM Na^+^, and 200 mM K^+^). After incubation time of 10 min at 28 °C, the fluorescence spectrum of the product (Resorufin) was measured using the fluorescence spectrophotometer (*λ*_ex_ = 571 nm).

For comparing the activities of the histidine-dependent DNAzyme and the hemin/G-quadruplex horseradish peroxidase-mimicking DNAzyme in the confined cavities with their activities in the homogenous solution, the activities of the DNAzymes in the two different conditions were measured at the same concentrations of the active DNAzymes (24.6 nM for the histidine-dependent DNAzyme and 17.9 nM for the hemin/G-quadruplex horseradish peroxidase-mimicking DNAzyme). The time-dependent fluorescence changes of the products generated by the histidine-dependent DNAzyme and the hemin/G-quadruplex horseradish peroxidase-mimicking DNAzyme were measured at *λ*_em_ = 518 nm and *λ*_em_ = 585 nm, respectively.

### Switchable unlocking and locking of nanoholes in origami O

The origami tile O (2 nM; in TAE buffer with 6 mM Mg^2+^ and 5 mM Na^+^) was irradiated under UV light (*λ* = 365 nm, 25 °C) for 5 min and then was added with the helper hairpins H_3_/H_4_ (10 nM for each one, 25 °C for 10 h) to generate the nanohole. For the reversible locking process, the anti-helper strands (H_3a_′/H_3b_′/H_4a_′/H_4b_′) (50 nM for each one) were added into the sample to remove the strands H_3_/H_4_ (25 °C for 2 h) and then the sample was irradiated with visible light (*λ* > 420 nm, 25 °C) for 10 min. For the second cycle of unlocking and locking processes, fivefold helper strands, and the anti-helper strands compared with the amount of the corresponding strands used in the forward steps were added into the sample, and the sample was irradiated under UV light (*λ* = 365 nm, 25 °C) and visible light (*λ* > 420 nm, 25 °C) for 5 min and 10 min for the two processes, respectively.

For the control experiments to unlock the tile O (2 nM), only the UV irradiation (*λ* = 365 nm) or the helper hairpins H_3_/H_4_ (10 nM for each one) was used.

Agarose electrophoreses of the locked and unlocked tiles O were performed in the TAE buffer with 6 mM Mg^2+^ and 5 mM Na^+^ (1%, 100 V, 1 h, 25 °C).

### Switchable catalysis in the nanocavity of origami tile O_f_

The tile O_f_ (30 nM, 200 μL, in TAE buffer with 6 mM Mg^2+^, 5 mM Na^+^, and 100 mM K^+^) was prepared for the measurements of the reversible catalysis. In the locked tile O_f_, the strands G_1×_ were activated by adding B_GX-1_′/B_GX-2_′ (300 nM for each, B_GX-1_′/B_GX-2_′: B_GX-1_/B_GX-2_ = 5: 1) to remove the block strands B_GX-1_/B_GX-2_ (25 °C for 1 h). For unlocking of the tile O_f_, the sample was irradiated under UV light (*λ* = 365 nm, 25 °C) for 5 min and then was added with the helper hairpins H_3_/H_4_ (300 nM for each one, 25 °C for 10 h) to generate the nanohole and form the G-quadruplexes in the cavity. To close the unlocked tile O_f_, crown ether (CE) (60 mM, 1800 μL; CE: K^+^ = 5: 1) and the block strands B_GX-1_/B_GX-2_ (3 μM, 150 μL; B_GX-1_/B_GX-2_: G_1×_ = 10: 1) were added into the sample to anneal from 30 °C to 10 °C for 2 h. Then the anti-helper strands H_3a_′/H_3b_′/H_4a_′/H_4b_′ (6 μM, 150 μL; H_3a_′/H_3b_′/H_4a_′/H_4b_′: H_3_/H_4_ = 5: 1) were added into the sample to remove the strands H_3_/H_4_ (25 °C for 2 h) and then the sample was irradiated with visible light (*λ* > 420 nm, 25 °C) for 10 min. The sample was centrifuged to remove the excess helpers/anti-helpers/CE (100 k NMWL, 3000 × *g*, 10 min, three times). In order to measure the catalytic activities of origami O_f_ in locked and unlocked states, 37.5 μL of the sample in respective state (set at 20 nM) was treated with the hemin (300 nM, 5 μL), Amplex Red (2 mM, 2.5 μL), and H_2_O_2_ (50 mM, 5 μL), and the mixture was incubated at 28 °C for 10 min. The fluorescence spectrum of the product (Resorufin) was measured using the fluorescence spectrophotometer (*λ*_ex_ = 571 nm). It should be noted that the reversibility of the photochemical opening and closure of the cavities is presented as a proof-of-concept characterizing the system for two cycles. In principle, we find that the reversible switching of the system can be extended to additional cycles. Nonetheless, the addition of the fuel and anti-fuel strands for each cycle diluted the sample and the repeated opening/closure of the cavities introduced accompanying damaged origami structures that perturb the reversible functions of the system. We find that after four cycles the switching degree decreased to 50%.

### AFM imaging

For the AFM measurements, 2 μL of the respective origami tile samples were deposited on the surface of the freshly peeled mica to adsorb for 5 min. The samples were imaged under tapping mode in an aqueous buffer using SNL-10 probes (Bruke, Multimode Nanoscope VIII). Imaging was performing with a spring constant of 0.35 N m^−1^ or with a spring constant of 0.24 N m^−1^ and at a tapping frequency of ~9 Hz in buffer. Both of the conditions yielded very similar images.

The statistical analyses of the different systems included three different methods as follows: (i) scanning and analyzing of arbitrary four different domains of the same origami droplet deposited on the mica; (ii) scanning and analyzing of arbitrary four domains of four different droplets of the same origami mixtures deposited on different mica supports; (iii) the preparation of at least two different samples of the respective origami structures and analyzing at least two arbitrary domains of the same origami sample. The different methods led to very similar statistical analysis results ± 3%.

In addition, the statistical analyses of the AFM images revealed some incomplete origami structures. These imperfect structures included ether damaged origami tiles or structures where the open cavities were questionable. These structures were included in Supplementary Tables 1–16 as imperfect structures.

## Supplementary information

Supplementary Information

Peer Review File

Source Data

## Data Availability

All Data supporting the findings of this manuscript are available from the corresponding author upon reasonable request. The source data underlying Figs. 1c, d, h, i, and 3c, d, and Supplementary Figs. 6c, 20b, 21b, and 27 are provided as a Source Data file.

## References

[1] Hong F, Zhang F, Liu Y, Yan H (2017). DNA origami: scaffolds for creating higher order structures. Chem. Rev..

[2] Rothemund PWK (2006). Folding DNA to create nanoscale shapes and patterns. Nature.

[3] Endo M, Sugiyama H (2014). Single-molecule imaging of dynamic motions of biomolecules in DNA origami nanostructures using high-speed atomic force microscopy. Acc. Chem. Res..

[4] Knudsen JB (2015). Routing of individual polymers in designed patterns. Nat. Nanotechnol..

[5] Wang Z-G, Liu Q, Ding B (2014). Shape-controlled nanofabrication of conducting polymer on planar DNA templates. Chem. Mater..

[6] Udomprasert A (2014). Amyloid fibrils nucleated and organized by DNA origami constructions. Nat. Nanotechnol..

[7] Ding B (2010). Gold nanoparticle self-similar chain structure organized by DNA origami. J. Am. Chem. Soc..

[8] Wilner OI, Willner I (2012). Functionalized DNA nanostructures. Chem. Rev..

[9] Liu W, Halverson J, Tian Y, Tkachenko AV, Gang O (2016). Self-organized architectures from assorted DNA-framed nanoparticles. Nat. Chem..

[10] Fu J, Liu M, Liu Y, Woodbury NW, Yan H (2012). Interenzyme substrate diffusion for an enzyme cascade organized on spatially addressable DNA nanostructures. J. Am. Chem. Soc..

[11] Ngo TA, Nakata E, Saimura M, Morii T (2016). Spatially organized enzymes drive cofactor-coupled cascade reactions. J. Am. Chem. Soc..

[12] Acuna GP (2012). Fluorescence enhancement at docking sites of DNA-directed self-assembled nanoantennas. Science.

[13] Zhan PF (2018). DNA origami directed assembly of gold bowtie nanoantennas for single-molecule surface-enhanced raman scattering. Angew. Chem. Int. Ed..

[14] Cecconello A, Besteiro LV, Govorov AO, Willner I (2017). Chiroplasmonic DNA-based nanostructures. Nat. Rev. Mater..

[15] Kuzyk A (2012). DNA-based self-assembly of chiral plasmonic nanostructures with tailored optical response. Nature.

[16] Wickham SFJ (2011). Direct observation of stepwise movement of a synthetic molecular transporter. Nat. Nanotechnol..

[17] Omabegho T, Sha R, Seeman NC (2009). A bipedal DNA brownian motor with coordinated legs. Science.

[18] Gu H, Chao J, Xiao S-J, Seeman NC (2010). A proximity-based programmable DNA nanoscale assembly line. Nature.

[19] Lund K (2010). Molecular robots guided by prescriptive landscapes. Nature.

[20] Kopperger E (2018). A self-assembled nanoscale robotic arm controlled by electric fields. Science.

[21] Jiang Q (2017). Stimulus-responsive plasmonic chiral signals of gold nanorods organized on DNA origami. Nano Lett..

[22] Kuzyk A (2016). A light-driven three-dimensional plasmonic nanosystem that translates molecular motion into reversible chiroptical function. Nat. Commun..

[23] Lan X (2018). DNA-guided plasmonic helix with switchable chirality. J. Am. Chem. Soc..

[24] Wang J, Zhou Z, Yue L, Wang S, Willner I (2018). Switchable triggered interconversion and reconfiguration of DNA origami dimers and their use for programmed catalysis. Nano Lett..

[25] Wu N, Willner I (2016). pH-stimulated reconfiguration and structural isomerization of origami dimer and trimer systems. Nano Lett..

[26] Wu N, Willner I (2016). DNAzyme-controlled cleavage of dimer and trimer origami tiles. Nano Lett..

[27] Wu N, Willner I (2017). Programmed dissociation of dimer and trimer origami structures by aptamer-ligand complexes. Nanoscale.

[28] Yang Y, Endo M, Hidaka K, Sugiyama H (2012). Photo-controllable DNA origami nanostructures assembling into predesigned multiorientational patterns. J. Am. Chem. Soc..

[29] Wang J, Yue L, Wang S, Willner I (2018). Triggered reversible reconfiguration of G-quadruplex-bridged “domino”-type origami dimers: application of the systems for programmed catalysis. ACS Nano.

[30] Andersen ES (2009). Self-assembly of a nanoscale DNA box with a controllable lid. Nature.

[31] Wagenbauer KF, Sigl C, Dietz H (2017). Gigadalton-scale shape-programmable DNA assemblies. Nature.

[32] Zhou C, Duan X, Liu N (2015). A plasmonic nanorod that walks on DNA origami. Nat. Commun..

[33] Chen YH (2018). A synthetic light-driven substrate channeling system for precise regulation of enzyme cascade activity based on DNA origami. J. Am. Chem. Soc..

[34] Zhang Q (2014). DNA origami as an in vivo drug delivery vehicle for cancer therapy. ACS Nano.

[35] Zhao YX (2012). DNA origami delivery system for cancer therapy with tunable release properties. ACS Nano.

[36] Douglas SM, Bachelet I, Church GM (2012). A logic-gated nanorobot for targeted transport of molecular payloads. Science.

[37] Chatterjee G, Dalchau N, Muscat RA, Phillips A, Seelig G (2017). A spatially localized architecture for fast and modular DNA computing. Nat. Nanotechnol..

[38] Funck T, Nicoli F, Kuzyk A, Liedl T (2018). Sensing picomolar concentrations of RNA using switchable plasmonic chirality. Angew. Chem. Int. Ed..

[39] Bell NAW (2012). DNA origami nanopores. Nano Lett..

[40] Wei RS, Martin TG, Rant U, Dietz H (2012). DNA origami gatekeepers for solid-state nanopores. Angew. Chem. Int. Ed..

[41] Ouyang XY (2017). Docking of antibodies into the cavities of DNA origami structures. Angew. Chem. Int. Ed..

[42] Kurokawa T (2018). DNA origami scaffolds as templates for functional tetrameric Kir3 K^+^ Channels. Angew. Chem. Int. Ed..

[43] Hernandez-Ainsa S (2013). DNA origami nanopores for controlling DNA translocation. ACS Nano.

[44] Seifert A (2015). Bilayer-spanning DNA nanopores with voltage-switching between open and closed state. ACS Nano.

[45] Burns JR, Seifert A, Fertig N, Howorka S (2016). A biomimetic DNA-based channel for the ligand-controlled transport of charged molecular cargo across a biological membrane. Nat. Nanotechnol..

[46] Freeman R, Liu X, Willner I (2011). Chemiluminescent and chemilunimeescence resonance energy transfer (CRET) detection of DNA, metal ions, and aptamer-substrate complexes using hemin/G-quadruplexes and CdSe/ZnS quantum dots. J. Am. Chem. Soc..

[47] Xiao Y (2004). Catalytic beacons for the detection of DNA and telomerase activity. J. Am. Chem. Soc..

[48] Freage L, Wang F, Orbach R, Willner I (2014). Multiplexed analysis of genes and of metal ions using enzyme/DNAzyme amplification machineries. Anal. Chem..

[49] Zhou W, Saran R, Liu J (2017). Metal sensing by DNA. Chem. Rev..

[50] Cha T-G (2014). A synthetic DNA motor that transports nanoparticles along carbon nanotubes. Nat. Nanotechnol..

[51] Hu Y (2016). Reversible modulation of DNA-based hydrogel shapes by internal stress interactions. J. Am. Chem. Soc..

[52] Chen W-H (2017). Stimuli-responsive nucleic acid-functionalized metal-organic framework nanoparticles using pH- and metal-ion-dependent DNAzymes as locks. Chem. Sci..

[53] Travascio P, Li Y, Sen D (1998). DNA-enhanced peroxidase activity of a DNA aptamer-hemin complex. Chem. Biol..

[54] Breaker RR, Joyce GF (1994). Inventing and improving ribozyme function: rational design versus iterative selection methods. Trends Biotechnol..

[55] Chandra M, Sachdeva A, Silverman SK (2009). DNA-catalyzed sequence-specific hydrolysis of DNA. Nat. Chem. Biol..

[56] Breaker RR, Joyce GF (1994). A DNA enzyme that cleaves RNA. Chem. Biol..

[57] Roth A, Breaker RR (1998). An amino acid as a cofactor for a catalytic polynucleotide. Proc. Natl Acad. Sci. USA.

[58] Wang F, Liu X, Willner I (2015). DNA switches: from principles to applications. Angew. Chem. Int. Ed..

[59] Wilner OI (2009). Enzyme cascades activated on topologically programmed DNA scaffolds. Nat. Nanotechnol..

[60] Zhao Z (2016). Nanocaged enzymes with enhanced catalytic activity and increased stability against protease digestion. Nat. Commun..

[61] Yang Y (2019). Programming rotary motions with a hexagonal DNA nanomachine. Chem. Eur. J..

