# Supplementary material for: Active generation of nanoholes in DNA origami scaffolds for programmed catalysis in nanocavities
Source: Nat Commun. 2019 Oct 31;10:4963. doi: 10.1038/s41467-019-12933-9 (PMC6823506; doi:10.1038/s41467-019-12933-9)
Supplement: Supplementary file 1 — Supplementary Information [file 41467_2019_12933_MOESM1_ESM.pdf]

Supplementary Information

Active generation of nanoholes in DNA origami scaffolds for programmed catalysis in nanocavities

Wang et al.

This file includes:

Supplementary Figures 1 to 30

Supplementary Tables 1 to 24

Supplementary References

I. Supplementary Figures and Tables

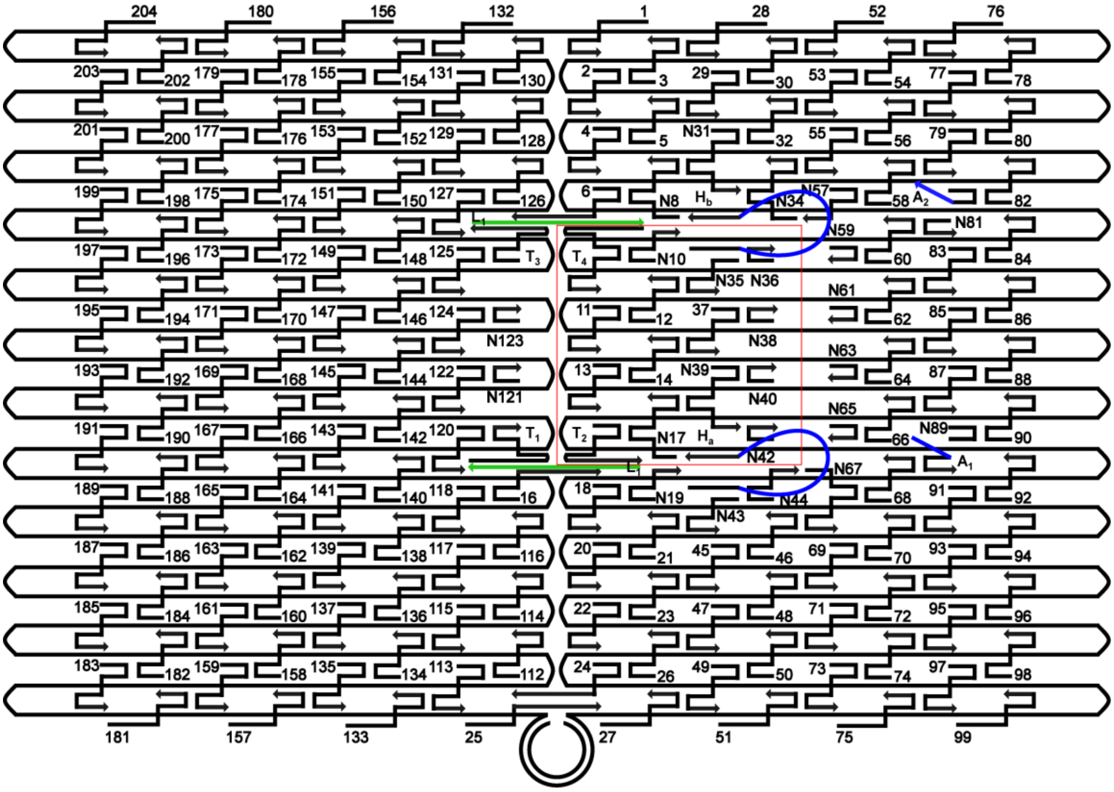

Supplementary Figure 1. Schematic of the designed origami tile N. The linker strand L_1 shows in green, the handles (H_a, H_b) are blue loops and the anchoring tethers (A_1, A_2) are short blue lines. The red square shows the area of the designed "window" in the origami tile N.

The designed "window" is a small inner part of the origami tile that is linked to the origami scaffold through the M13 strand on its right side[1]. The linker of M13 strand on the right side of the "window" is working as hinges for its opening process. The left side of the "window" is locked to the origami scaffold using two linker strands L_1 (green). One of the linkers hybridizes with the strand extending from the staple strand T_1 on the origami tile scaffold and the strand extending from the staple strand T_2 on the left side of the designed "window". Similarly, the other one interacts with T_3 and T_4. The linker strands L_1 contain the sequences of the substrate of the Zn^{2+}-ion-dependent DNAzyme and can be cleaved by the DNAzyme. The top and bottom sides of the designed "window" have no interaction with the origami scaffold except the handles (H_a and H_b, blue loop). One end of the handle is linked to the staple strand of the origami tile scaffold and the other end of the handle is linked to the staple of the "window". Two anchoring footholds (A_1 and A_2) are extending from the staple strands on the right side of the origami tile scaffold. The handles can facilitate the opening of the "unlocked window" through their hybridization with the helper strands that can bind to the anchoring tethers (in the form of $H_a/H_1/A_1$ and $H_b/H_2/A_2$). The staples on the left edge and right edge are removed to avoid the aggregation of the origami tiles[1].

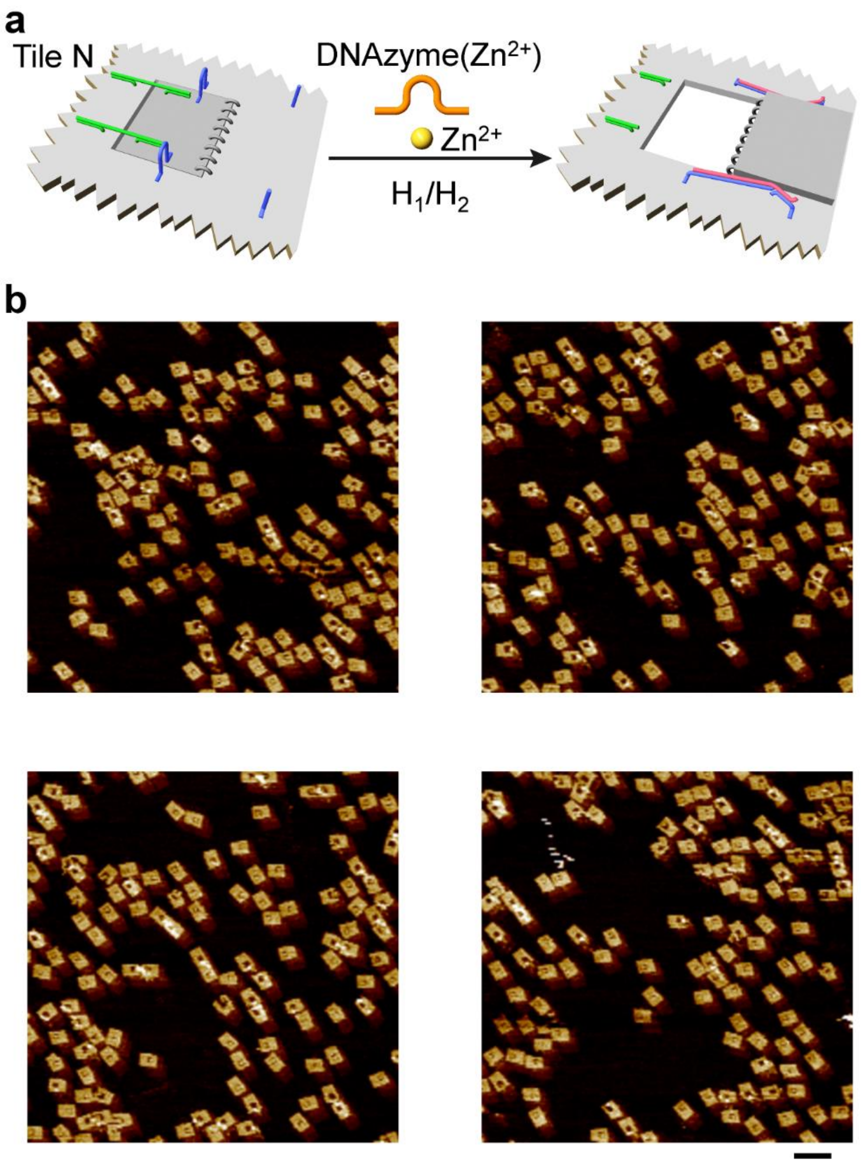

Supplementary Figure 2. Formation of nanoholes in origami tiles N. a, Schematic of the formation of the nanohole in the origami tile N using the Zn^{2+}-ion-dependent DNAzyme and the hairpin helper units H_1/H_2. **b,** Four AFM scanned areas of the Zn^{2+}-ion-dependent DNAzyme-driven unlocked origami tiles. Scale bar: 200 nm.

Supplementary Table 1. Statistical analysis of the yields of the unlocked and locked origami tiles N upon the addition of Zn^{2+} ions and the hairpin helper units H_1/H_2. Analysis of the unlocked and locked tiles in the four scanned areas shown in Supplementary Fig. 2b.

**Statistical analysis[a]**		**Unlocked**	**Locked**	**Incomplete structure**
**1**	Count	70	33	7
	Yield (%)	63.6	30	6.4
**2**	Count	72	22	6
	Yield (%)	72	22	6
**3**	Count	79	29	3
	Yield (%)	71.2	26.1	2.7
**4**	Count	71	25	6
	Yield (%)	69.6	24.5	5.9

a. The statistical analysis of the unlocked origami tiles involved the identification of intact origami structures that included open cavities. Locked structures were identified as tiles that included non-identified cavity. Imperfect structures were identified as tiles with damaged origami scaffolds or questionable evolved cavities.

Control experiments for the unlocking of the origami tiles by the Zn^{2+}-ion-dependent DNAzyme

Supplementary Fig. 3 shows the AFM images upon treatment of the origami tile N with hairpins H_1/H_2 in the absence of Zn^{2+} ions. No formation of nanoholes in the origami scaffold is observed implying that the activation of the Zn^{2+}-ion-dependent DNAzyme is essential to generate the nanoholes. Supplementary Fig. 4 show the AFM images of origami tiles N subjected to Zn^{2+} ions in the absence of the "stretching" hairpins H_1/H_2. The yield of nanohole-containing tiles is very low, ca. 8% as compared to 70% yield in the presence of H_1/H_2 (see text). These results indicate that the cleavage of the locks by the Zn^{2+}-ion-dependent DNAzyme itself is insufficient to control the "mechanical" opening of the "window". Only in the presence of H_1/H_2, the helper assisted stretching of the "window" through the helper units leads to the high-yield generation of the nanoholes. It should be noted that addition of H_1/H_2 to the low-yield origami mixture shown in Supplementary Fig. 4, stimulated the efficient high-yield unlocking of the origami structures, consistent with the H_1/H_2 mechanically driven opening of the nanoholes.

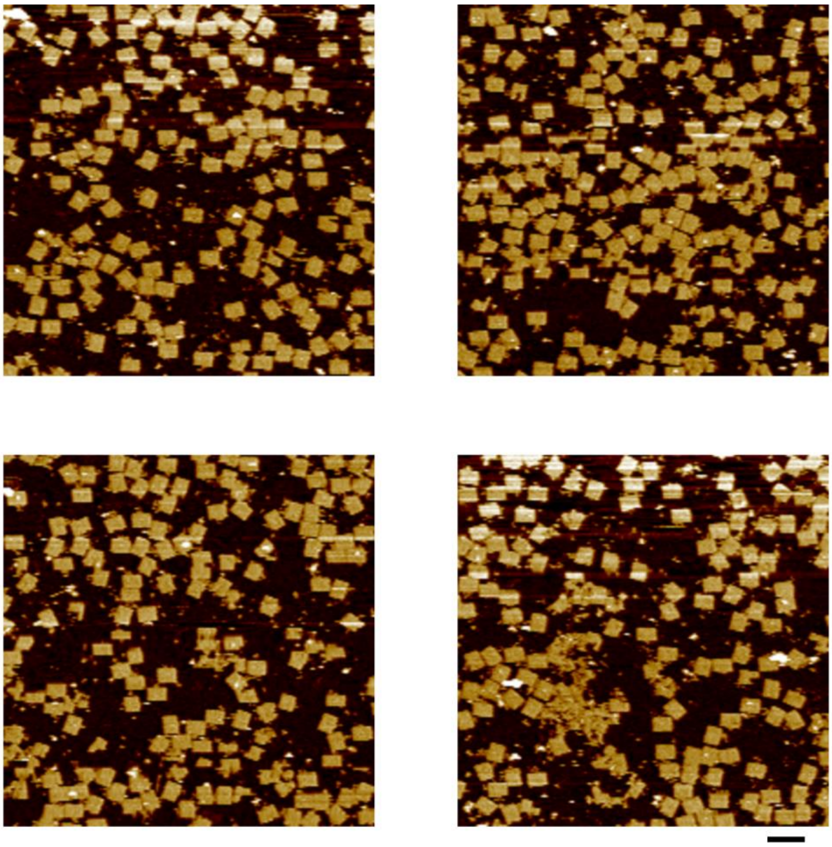

Supplementary Figure 3. AFM images of the control experiments examining the unlocking of the origami tiles N in the presence of the helper hairpins H_1/H_2 and the Zn^{2+}-ion-dependent DNAzyme sequence, in the absence of added Zn^{2+} ions. No tiles with nanoholes are observed, implying that the Zn^{2+}-ion-dependent DNAzyme is essential to unlock the origami tiles to yield the nanoholes. Scale bar: 200 nm.

Supplementary Table 2. Statistical analysis of the yields of the unlocked and locked origami tiles N upon the addition of the helper hairpins H_1/H_2 and the Zn^{2+}-ion-dependent DNAzyme sequence, in the absence of Zn^{2+} ions. Analysis of the unlocked and locked tiles in the four scanned areas shown in Supplementary Fig. 3.

Statistical analysis[a]		Unlocked	Locked	Incomplete structure
**1**	Count	0	135	13
	Yield (%)	0	91.2	8.8
**2**	Count	0	124	28
	Yield (%)	0	81.6	18.4
**3**	Count	0	116	26
	Yield (%)	0	81.7	18.3
**4**	Count	0	118	9
	Yield (%)	0	92.9	7.1

a. The statistical analysis of the locked origami tiles involved the identification of intact origami structures. Imperfect structures were identified as tiles with damaged origami scaffolds.

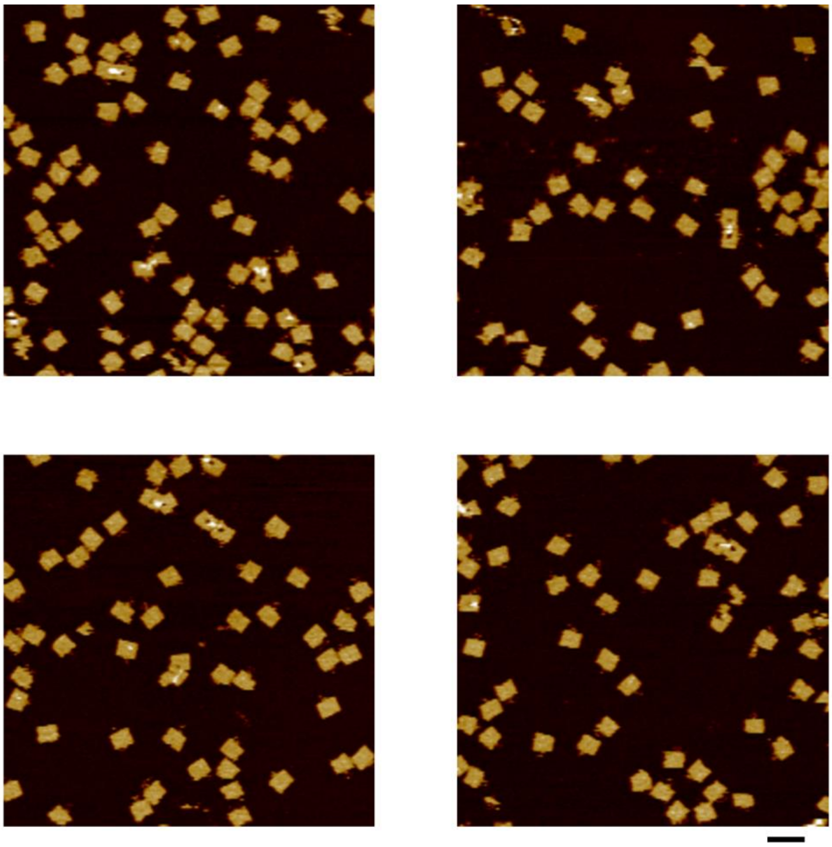

Supplementary Figure 4. AFM images of the control experiments examining the Zn^{2+}-ion-dependent DNAzyme unlocking of the origami tiles N in the absence of the hairpin helper units H_1/H_2. Only few origami tiles show open nanoholes. The results imply that the unlocked "window" exists in a flexible configuration that retains the nanohole closed, in the absence of the hairpin helper units H_1/H_2 that stretch the "window" to a rigid configuration by the hairpins/handles/anchor site units. Scale bar: 200 nm.

Supplementary Table 3. Statistical analysis of the yields of the unlocked and locked origami tiles N upon the addition of Zn^{2+} ions and the Zn^{2+}-ion-dependent DNAzyme sequence, in the absence of the helper H_1/H_2. Analysis of the unlocked and locked tiles in the four scanned areas shown in Supplementary Fig. 4.

**Statistical analysis[a]**		**Unlocked**	**Locked**	**Incomplete structure**
**1**	Count	5	46	1
	Yield (%)	9.6	88.5	1.9
**2**	Count	3	39	4
	Yield (%)	6.5	84.8	8.7
**3**	Count	4	55	5
	Yield (%)	6.3	85.9	7.8
**4**	Count	5	44	1
	Yield (%)	10	88	2

a. The statistical analysis of the unlocked origami tiles involved the identification of intact origami structures. Locked structures were identified as tiles with non-identified cavity. Imperfect structures were identified as tiles with damaged origami scaffolds or questionable evolved cavities.

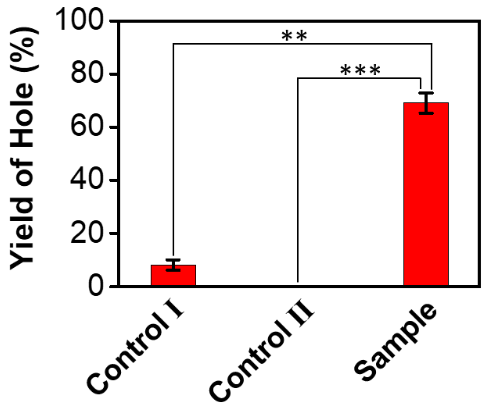

Supplementary Figure 5. Statistical analysis of the yields of nanoholes in the DNA origami N unlocking by Zn^{2+}-ion-dependent DNAzyme and the control measurement I without helper strands (H_1/H_2) or the control measurement II without Zn^{2+} ions and the statistically significant difference according to Student's T-test. **P < 0.001, ***P < 0.0005.

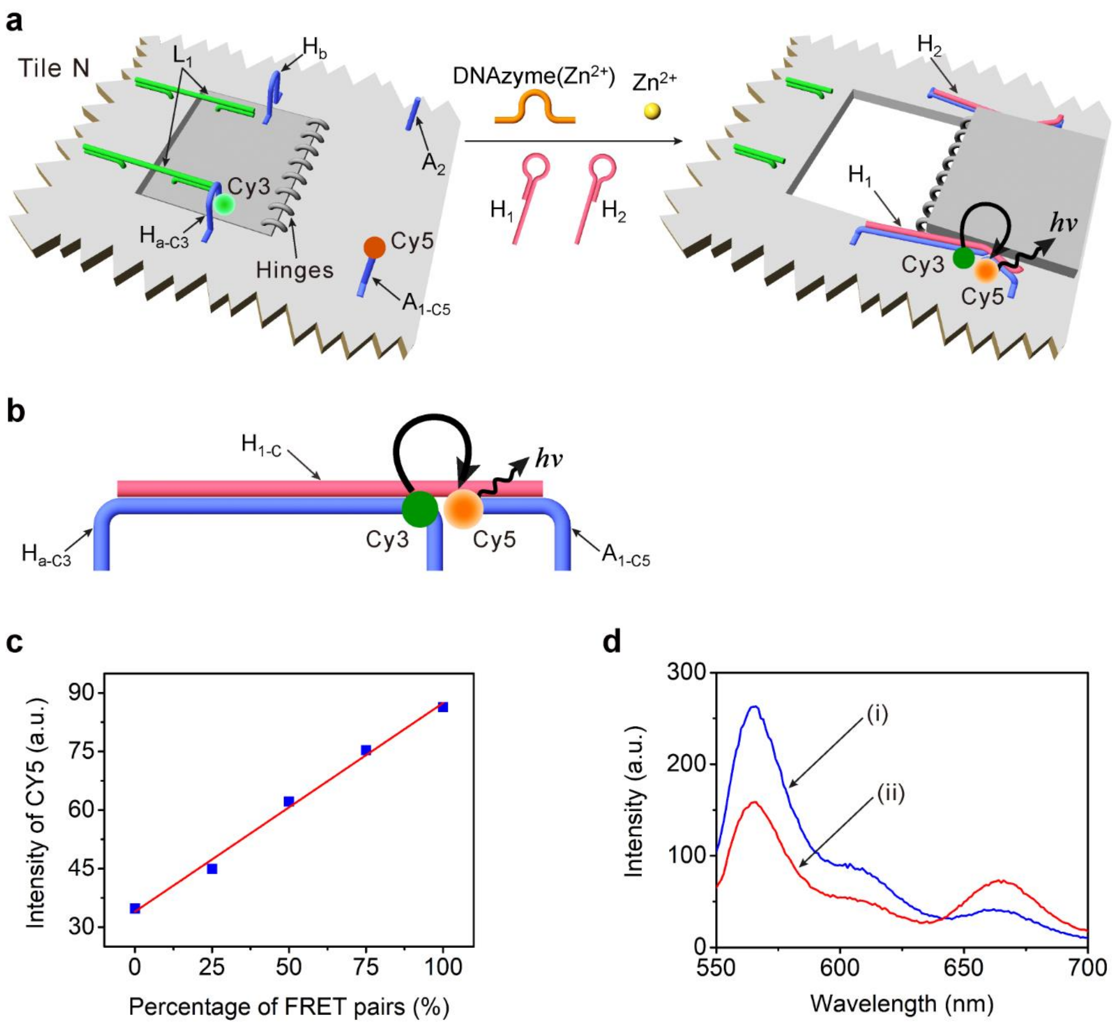

Supplementary Figure 6. Probing the Zn^{2+}-ion-dependent DNAzyme-stimulated formation of nanoholes in the origami tile using FRET. a, Analysis of the Zn^{2+}-ion-dependent DNAzyme-driven unlocking of the origami tile N by FRET. One of the handle unit associated with closed "window", $H_{a\text{-}C3}$, is internally modified by Cy3. The anchoring foothold linked to the origami scaffold $A_{1\text{-}C5}$ is functionalized with Cy5. The "window" is opened after its unlocking by the Zn^{2+}-ion-dependent DNAzyme, and using the helper hairpins H_1 and H_2 to fix the "window" to the footholds. In the resulting structure, the FRET signal provides an optical stimuli to follow the opening of the "window". **b,** In order to quantify the yield of the open cavities, a model construct consisting of the strands $H_{a\text{-}C3}$ and $A_{1\text{-}C5}$ (at concentration identical to their distribution on origami tile N, 20 nM) were crosslinked by variable concentrations of the strand $H_{1\text{-}C}$ (0 nM, 5 nM, 10 nM, 15 nM and 20 nM). The resulting FRET signal provided a quantitative measure for the hybrid model $(H_{a\text{-}C3}+A_{1\text{-}C5})/H_1$. **c,** Calibration curve corresponding to the FRET signal of the supramolecular model construct as a function of the percentage of FRET pairs. Source data are provided as a Source Data file. **d,** Luminescence spectra of: (i) The Zn^{2+}-ion-dependent DNAzyme-stimulated unlocked "window" prior to the addition of the helper units H_1 and H_2. (ii) After treatment of the Zn^{2+}-ion-dependent DNAzyme unlocked "window" with the helper units H_1 and H_2 and

the opening of the "window". In both spectra, λ_{ex} = 532 nm (for Cy3) and the FRET signal is monitored at λ_{em} = 665 nm. Note that the FRET process leads to the decrease of the fluorescence intensity of Cy3 at λ_{em} = 564 nm and the increase of the fluorescence intensity of Cy5 at λ_{em} = 665 nm.

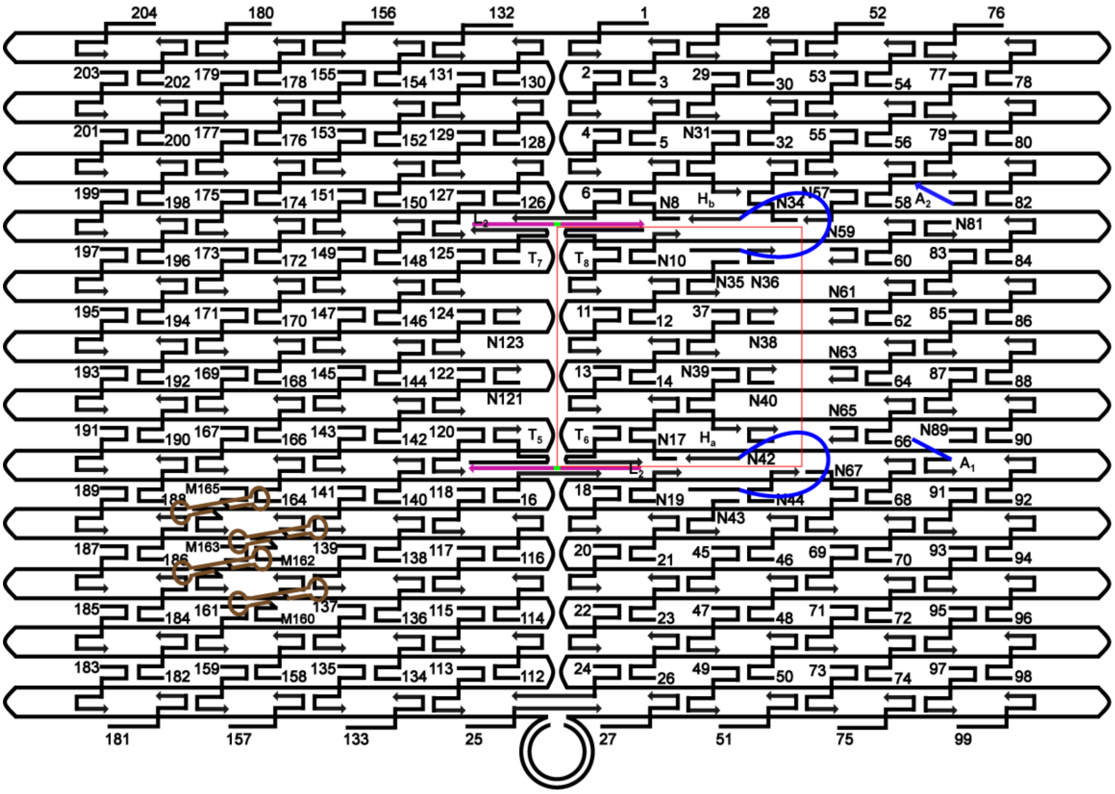

Supplementary Figure 7. Schematic of the designed origami tile M. The linker strand L_2 shows in purple, the handles (H_a, H_b) are blue loops and the anchoring tethers (A_1, A_2) are short blue lines. The red square shows the area of the designed "window" in the origami tile M. The four hairpins in brown are the labels on the bottom-left of the tile M.

The designed "window" is locked to the origami scaffold on its right side using two linker strands L_2 (purple)[1]. One of the linker L_2 is hybridized with the strand extending from the staple strand T_5 on the origami tile scaffold and the strand extending from the staple strand T_6 on the left side of the designed "window". The other one binds to the T_7 and T_8. The linker strands L_2 contain the sequences of the substrate of the Pb^{2+}-ion-dependent DNAzye that can unlock the "window" by the cleavage of the linker strands. The handles and the anchoring footholds are the same as for the origami tile N in Supplementary Fig. 1. The staples on the left edge and the right edge are removed to avoid the aggregation of the origami tiles[1].

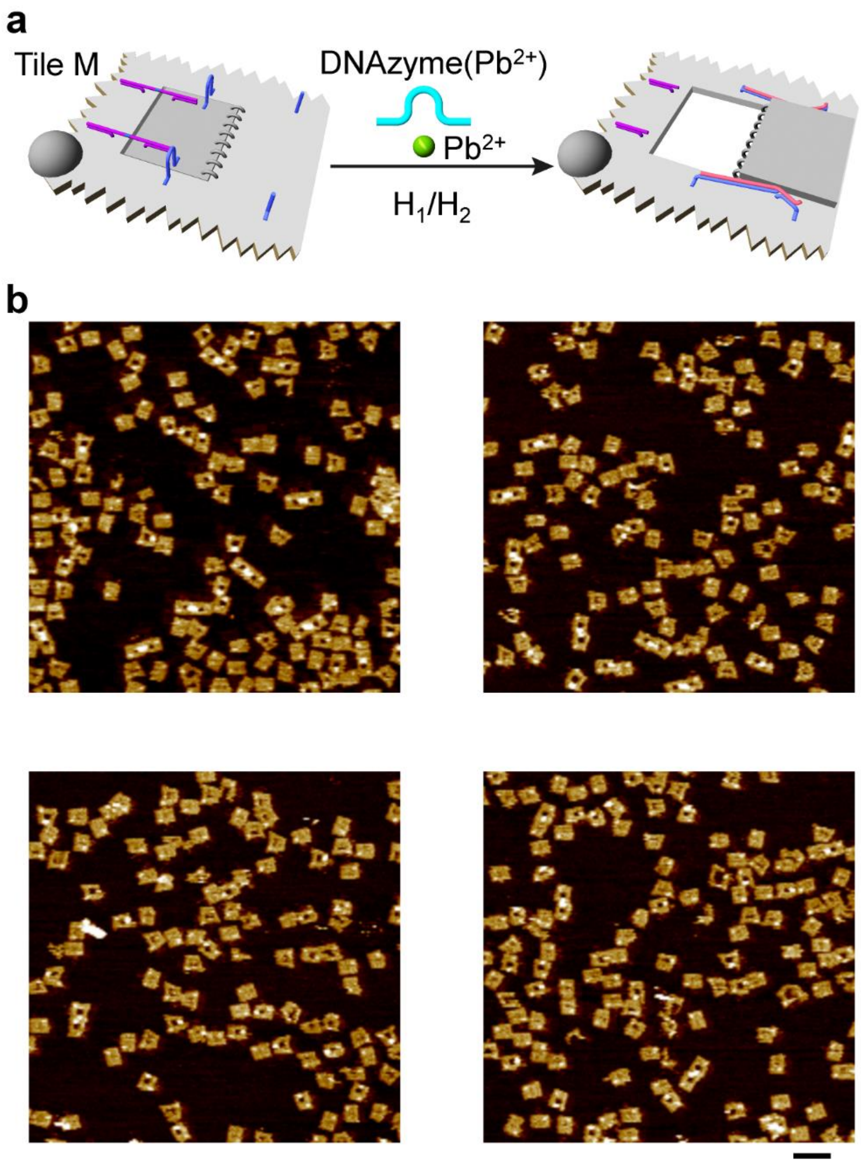

Supplementary Figure 8. Formation of nanoholes in origami tiles M. a, Schematic of the formation of the nanohole in the origami tile M using the Pb^{2+}-ion-dependent DNAzyme and the hairpin helper units H_1/H_2. **b,** Four AFM scanned areas of the Pb^{2+}-ion-dependent DNAzyme-driven unlocked origami tiles. Scale bar: 200 nm.

Supplementary Table 4. Statistical analysis of the yields of the unlocked and locked origami tiles M upon the addition of Pb^{2+} ions and the hairpin helper units H_1/H_2. Analysis of the unlocked and locked tiles in the four scanned areas shown in Supplementary Fig. 8b.

Statistical analysis[a]		Unlocked	Locked	Incomplete structure
**1**	Count	56	16	4
	Yield (%)	73.7	21.1	5.3
**2**	Count	63	16	5
	Yield (%)	75	19.0	6.0
**3**	Count	69	28	3
	Yield (%)	69	28	3
**4**	Count	66	19	5
	Yield (%)	73.3	21.1	5.6

a. The statistical analysis of the unlocked origami tiles involved the identification of intact origami structures that included open cavities and clear markers units on the tiles. Locked structures were identified as tiles that included a clear marker with non-identified cavity. Imperfect tiles were identified as damaged origami scaffolds.

Control experiments for the unlocking of the origami tiles by the Pb^{2+}-ion-dependent DNAzyme

Supplementary Fig. 9 shows the AFM images upon treatment of the origami tile M with hairpins H_1/H_2 in the absence of Pb^{2+} ions. No formation of nanoholes in the origami scaffold is observed implying that the activation of the Pb^{2+}-ion-dependent DNAzyme is essential to generate the holes. Supplementary Fig. 10 show the AFM images of origami tiles M subjected to Pb^{2+} ions in the absence of the "stretching" hairpins H_1/H_2. The yield of nanohole-containing tiles is very low, ca. 8% as compared to 72% yield in the presence of H_1/H_2 (see text). These results indicate that the cleavage of the locks by the Pb^{2+}-ion-dependent DNAzyme is insufficient to control the "mechanical" opening of the "window". Only in the presence of H_1/H_2, the helper assisted stretching of the "window" through the helper units leads to the high-yield generation of the nanoholes. It should be noted that addition of H_1/H_2 to the low-yield origami mixture shown in Supplementary Fig. 10, stimulated the efficient high-yield unlocking of the origami structures, consistent with the H_1/H_2 mechanically driven opening of the nanoholes.

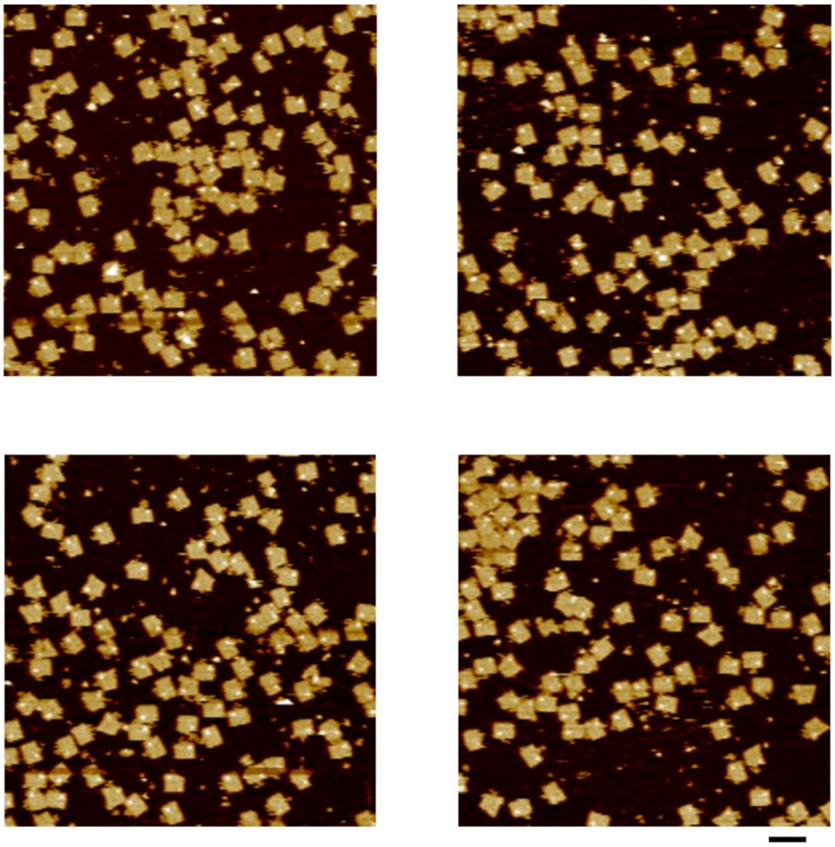

Supplementary Figure 9. AFM images of the control experiments examining the unlocking of the origami tiles M in the presence of the helper hairpins H_1/H_2 and the Pb^{2+}-ion-dependent DNAzyme sequence, in the absence of added Pb^{2+} ions. No tiles with nanoholes are observed, implying that the Pb^{2+}-ion-dependent DNAzyme is essential to unlock the origami tiles to yield the nanoholes. Scale bar: 200 nm.

Supplementary Table 5. Statistical analysis of the yields of the unlocked and locked origami tiles M upon the addition of the helper hairpins H_1/H_2 and the Pb^{2+}-ion-dependent DNAzyme sequence, in the absence of Pb^{2+} ions. Analysis of the unlocked and locked tiles in the four scanned areas shown in Supplementary Fig. 9.

**Statistical analysis**[a]		**Unlocked**	**Locked**	**Incomplete structure**
**1**	Count	0	93	8
	Yield (%)	0	92.1	7.9
**2**	Count	0	83	3
	Yield (%)	0	96.5	3.5
**3**	Count	0	84	2
	Yield (%)	0	97.7	2.3
**4**	Count	0	88	3
	Yield (%)	0	96.7	3.3

a. The statistical analysis of the locked origami tiles involved the identification of intact origami structures without cavity. Imperfect tiles were identified as damaged origami scaffolds.

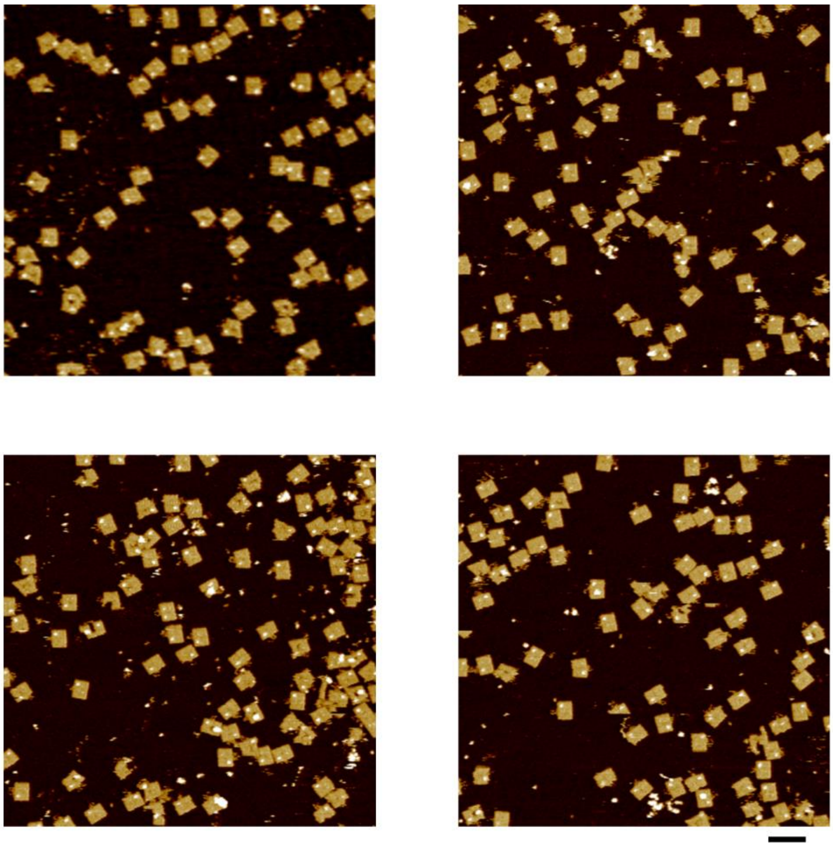

Supplementary Figure 10. AFM images of the control experiments examining the Pb^{2+}-ion-dependent DNAzyme unlocking of the origami tiles M in the absence of the hairpin helper units H_1/H_2. Only few origami tiles show open nanoholes. The results imply that the unlocked "window" exists in a flexible configuration that retains the nanohole closed, in the absence of the hairpin helper units H_1/H_2 that stretch the "window" to a rigid configuration by the hairpins/handles/anchor site units. Scale bar: 200 nm.

Supplementary Table 6. Statistical analysis of the yields of the unlocked and locked origami tiles M upon the addition of Pb^{2+} ions and the Pb^{2+}-ion-dependent DNAzyme sequence, in the absence of the helper hairpins H_1/H_2. Analysis of the unlocked and locked tiles in the four scanned areas shown in Supplementary Fig. 10.

**Statistical analysis[a]**		**Unlocked**	**Locked**	**Incomplete structure**
**1**	Count	6	61	1
	Yield (%)	8.7	89.7	1.5
**2**	Count	7	61	7
	Yield (%)	9.3	81.3	9.3
**3**	Count	5	52	8
	Yield (%)	7.7	80	12.3
**4**	Count	4	62	2
	Yield (%)	5.9	91.2	2.9

a. The statistical analysis of the unlocked origami tiles involved the identification of intact origami structures that included open cavities and clear markers units on the tiles. Locked structures were identified as tiles that included a clear marker with non-identified cavity. Imperfect tiles were identified as damaged origami scaffolds.

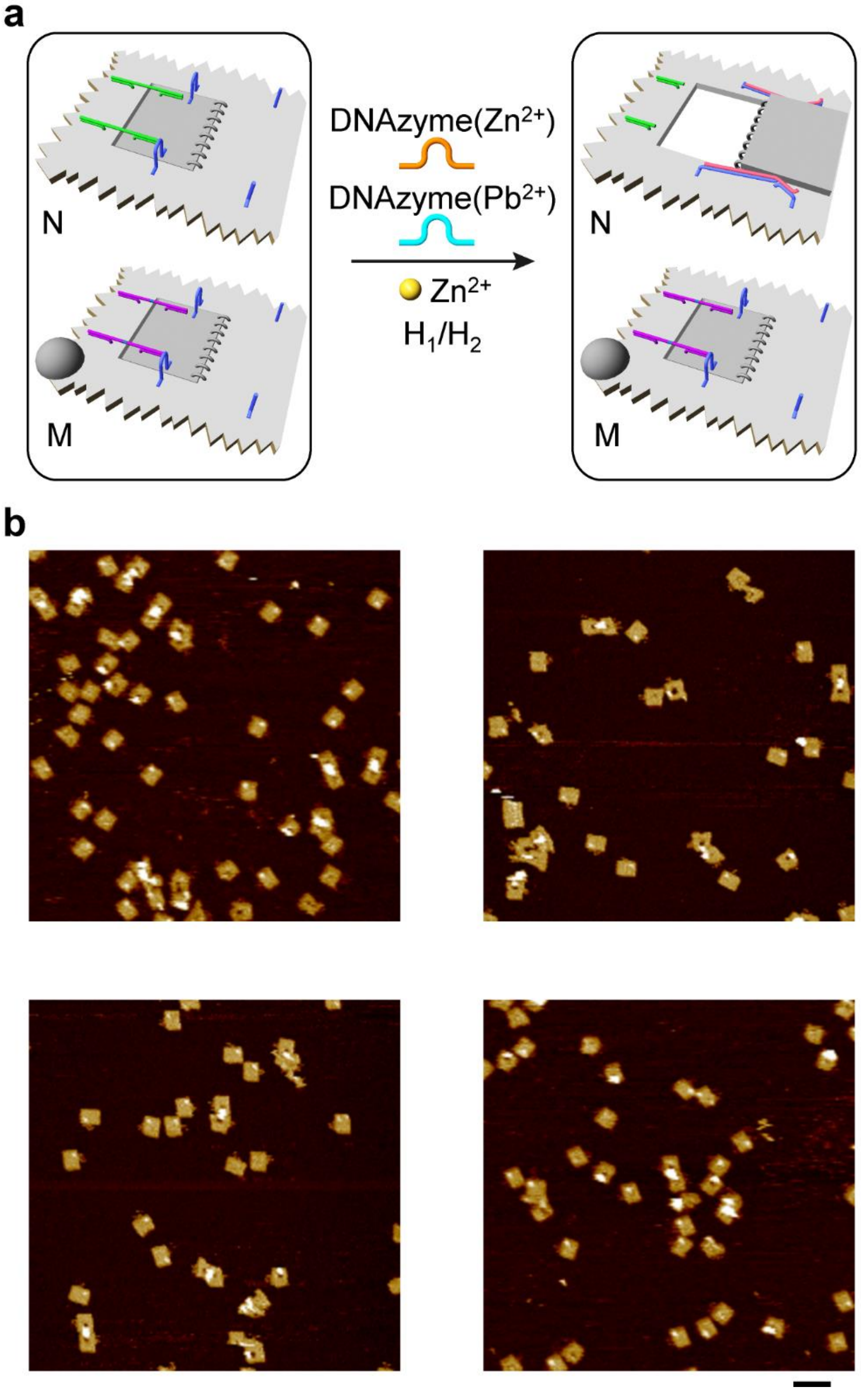

Supplementary Figure 11. Selective formation of the nanohole associated with the origami tile N in the mixture of N and M. a, Schematic of the selective unlocking of the origami tile N in the tile mixture of N and M by adding Zn^{2+} ions and the hairpins H_1/H_2, in the presence of Zn^{2+}-ion- and Pb^{2+}-ion-dependent DNAzyme sequences. **b,** Four AFM images corresponding to the selective formation of nanoholes in origami tiles N in the tile mixture of N and M. Scale bar: 200 nm.

Supplementary Table 7. Statistical analysis of the yields of the unlocked and locked origami tiles N and M by subjecting the tile mixture to Zn^{2+} ions and the hairpins H_1/H_2, in the presence of Zn^{2+}-ion- and Pb^{2+}-ion-dependent DNAzyme sequences. The analysis is performed on the images shown in Supplementary Fig. 11b.

Statistical analysis[a]		Origami N			Origami M	
		Unlocked	Locked	Incomplete structure	Unlocked	Locked
**1**	Count	20	6	3	1	22
	Yield (%)	69.0	20.7	10.3	4.3	95.7
**2**	Count	8	2	1	1	12
	Yield (%)	72.7	18.2	9.1	7.7	92.3
**3**	Count	10	3	1	0	12
	Yield (%)	71.4	21.4	7.1	0	100
**4**	Count	11	5	1	1	15
	Yield (%)	64.7	29.4	5.9	6.3	93.7

a. The statistical analysis of the unlocked origami tiles involved the identification of intact origami structures that included open cavities on no marker units. In parallel, marker-containing scaffold with an open cavity were searched (very low content of such structures were identified). Imperfect structures were identified as non-marker containing tiles with damaged origami scaffolds or questionable evolved cavities.

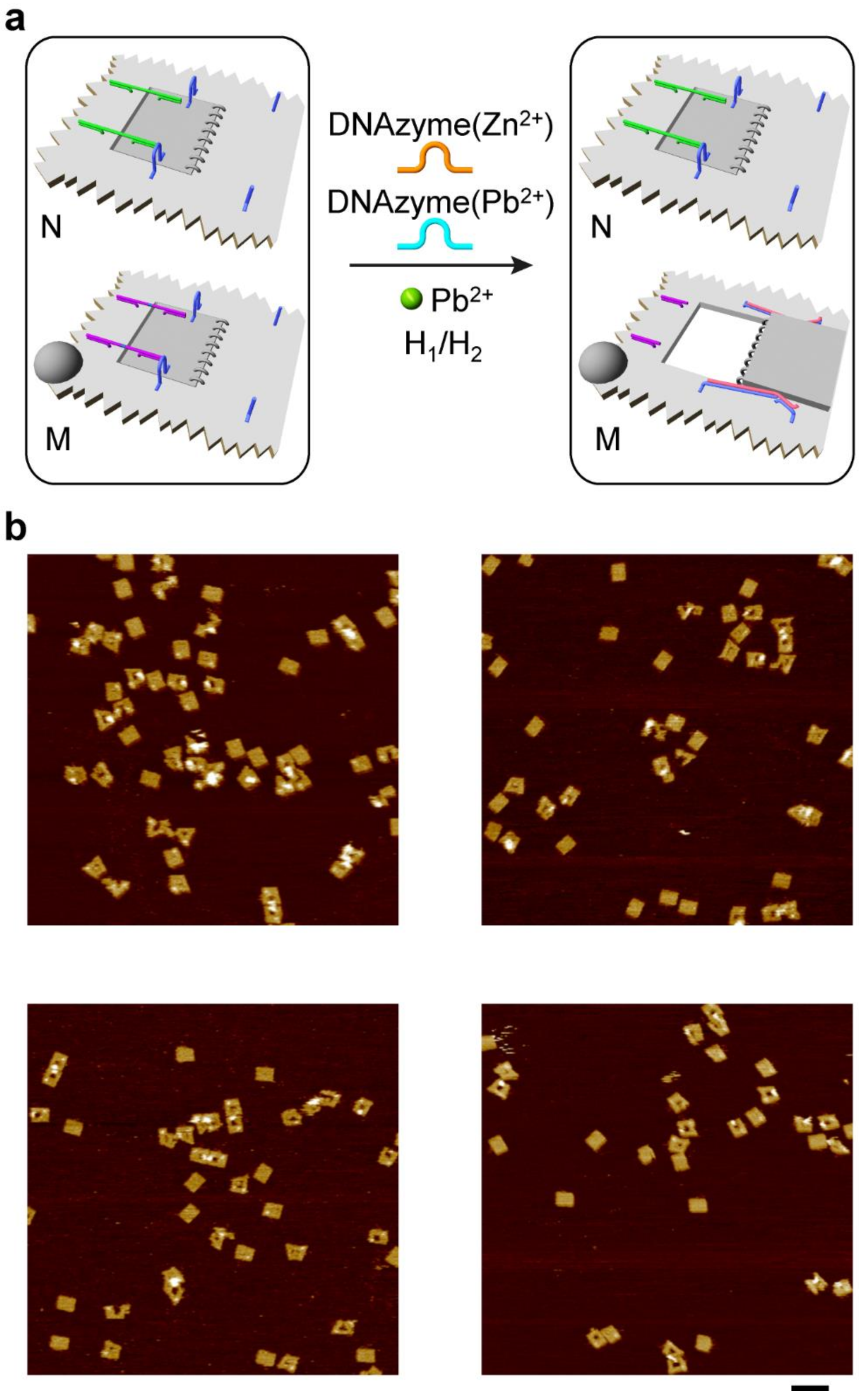

Supplementary Figure 12. Selective formation of the nanohole in the origami tile M of the mixture of N and M. a, Schematic of the selective unlocking of the origami tile M in the mixture of N and M by adding Pd^{2+} ions and the hairpins H_1/H_2, in the presence to Zn^{2+}-ion- and Pb^{2+}-ion-dependent DNAzyme sequences. **b,** Four AFM images corresponding to the selective formation of nanoholes in tiles M in the mixture of N and M. Scale bar: 200 nm.

Supplementary Table 8. Statistical analysis of the yields of the unlocked and locked origami tiles N and M by subjecting the tile mixture to Pb^{2+} ions and the hairpins H_1/H_2, in the presence of Zn^{2+}-ion- and Pb^{2+}-ion-dependent DNAzyme sequences. The analysis is performed on the images shown in Supplementary Fig. 12b.

Statistical analysis[a]		Origami N		Origami M		
		Unlocked	Locked	Unlocked	Locked	Incomplete structure
**1**	Count	0	20	16	4	3
	Yield (%)	0	100	69.6	17.4	13.0
**2**	Count	0	19	12	2	2
	Yield (%)	0	100	75	12.5	12.5
**3**	Count	0	13	16	4	2
	Yield (%)	0	100	72.7	18.2	9.1
**4**	Count	0	13	13	4	1
	Yield (%)	0	100	72.2	22.2	5.6

a. The statistical analysis of the unlocked origami tiles involved the identification of intact origami structures that included open cavities and clear markers units on the respective open tiles. Locked structures were identified as tiles that included a clear marker with non-identified cavity. Imperfect tiles were identified as marker-containing tiles with damaged origami scaffolds.

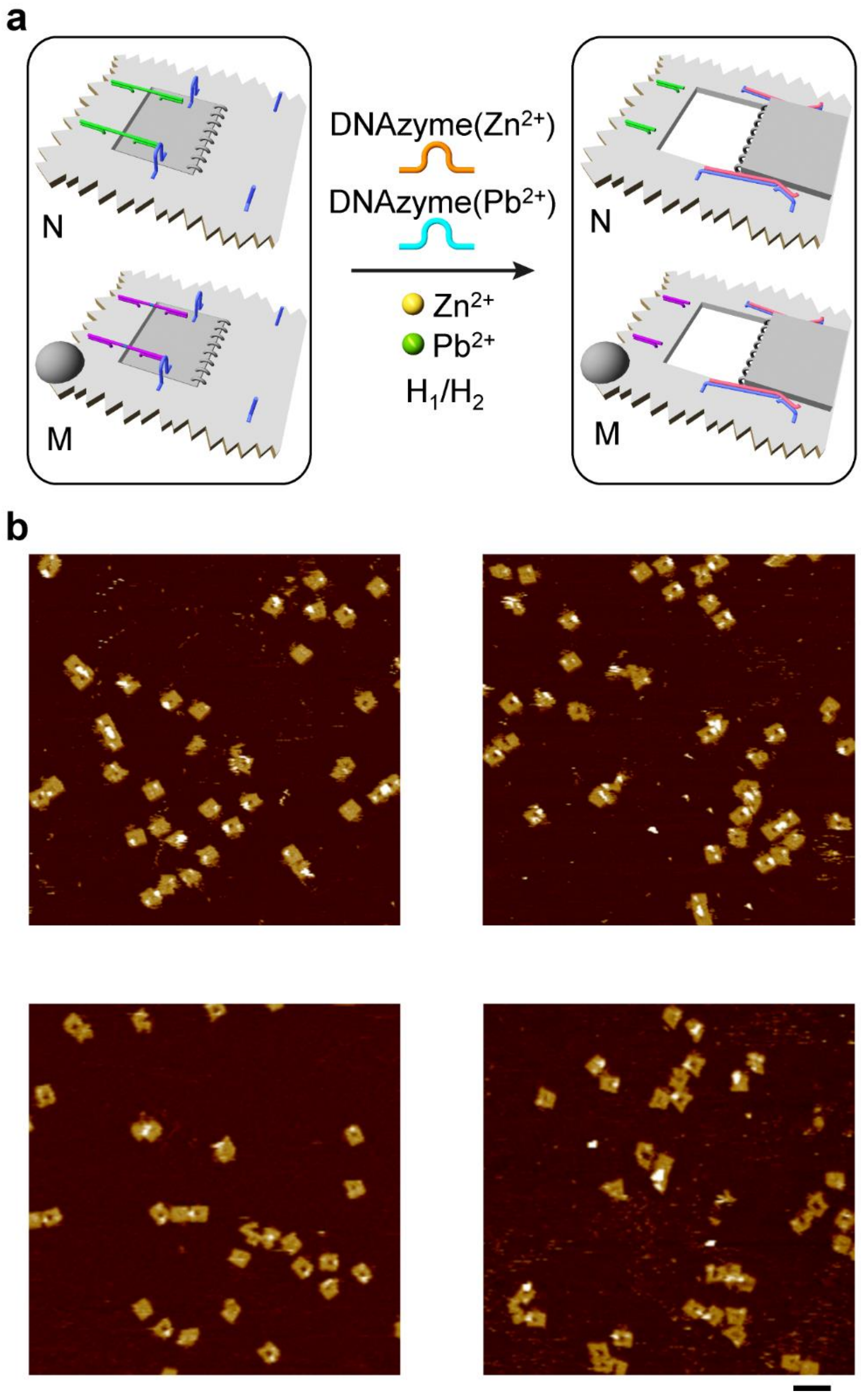

Supplementary Figure 13. Programmed formation of nanoholes in the origami tile mixture of N and M. a, Schematic of the programmed unlocking of the origami tiles N and M and formation of nanoholes upon the treatment of the mixture with Zn^{2+} ions and Pb^{2+} ions and the helper hairpins H_1/H_2, in the presence of Zn^{2+}-ion- and Pb^{2+}-ion-dependent DNAzyme sequences. **b,** Four AFM images of the mixture of origami tiles N and M with programmed nanoholes. Scale bar: 200 nm.

Supplementary Table 9. Statistical analysis of the yields of the unlocked and locked origami tiles N and M upon the treatment of the tile mixture of N and M with Zn^{2+} ions and Pb^{2+} ions and the helper units H_1/H_2, in the presence of Zn^{2+}-ion- and Pb^{2+}-ion-dependent DNAzyme sequences. The analysis is performed on the images shown in Supplementary Fig. 13b.

Statistical analysis[a]		Origami N			Origami M		
		**Unlocked**	**Locked**	**Incomplete structure**	**Unlocked**	**Locked**	**Incomplete structure**
**1**	Count	10	3	1	10	3	1
	Yield (%)	71.4	21.4	7.1	71.4	21.4	7.1
**2**	Count	11	2	2	13	3	1
	Yield (%)	73.3	13.3	13.3	76.5	17.6	5.9
**3**	Count	7	2	1	7	2	0
	Yield (%)	70	20	10	77.8	22.2	0
**4**	Count	8	2	1	8	2	1
	Yield (%)	72.7	18.2	9.1	72.7	18.2	9.1

a. The statistical analysis of the unlocked origami tiles involved the identification of intact origami structures that included open cavities and without marker or with clear markers units on the respective tiles. Locked structures were identified as tiles that included non-identified cavity with and without clear marker. Imperfect tiles were identified as no marker or marker-containing tiles with damaged origami scaffolds.

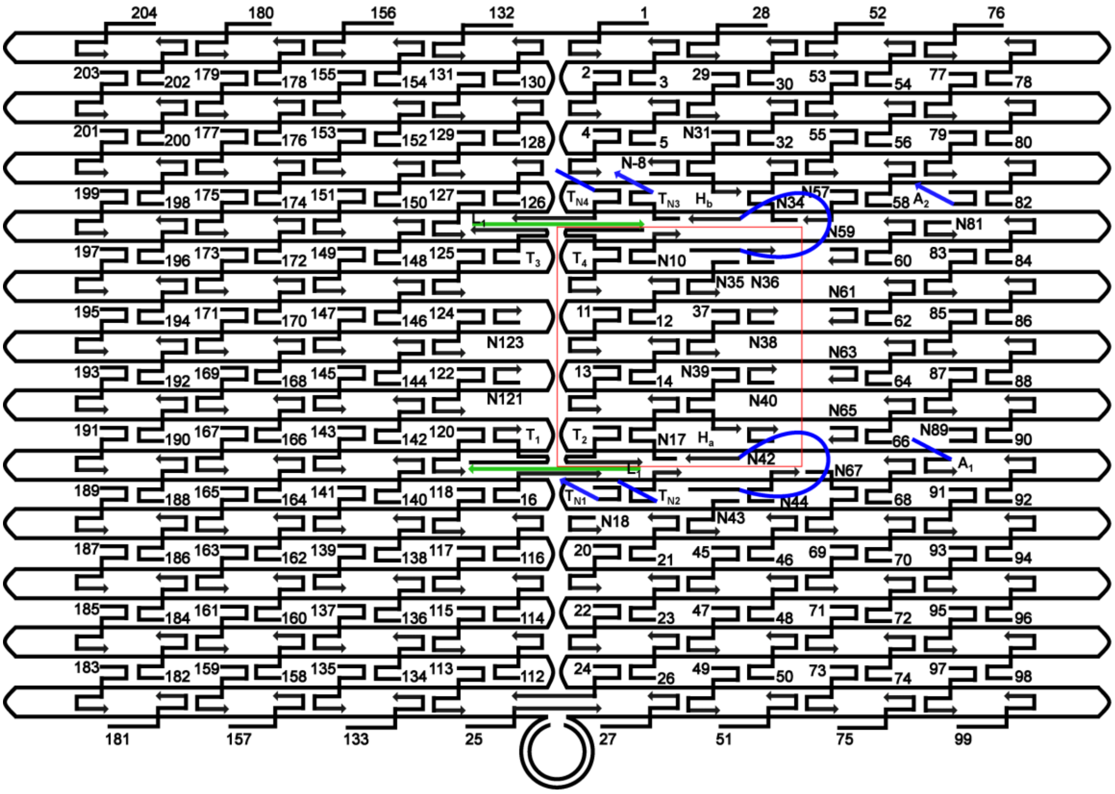

Supplementary Figure 14. Schematic of the designed origami tile N_f. The tile is functionalized with two pairs of tethers T_{N1}/T_{N3} and T_{N2}/T_{N4} that hybridize with the K_{his} and E_{his} strands on opposite sides of the tile raft, respectively. The tile N_f includes the Zn^{2+}-ion-dependent DNAzyme-driven unlocking apparatus in tile N, as shown in Supplementary Fig. 1.

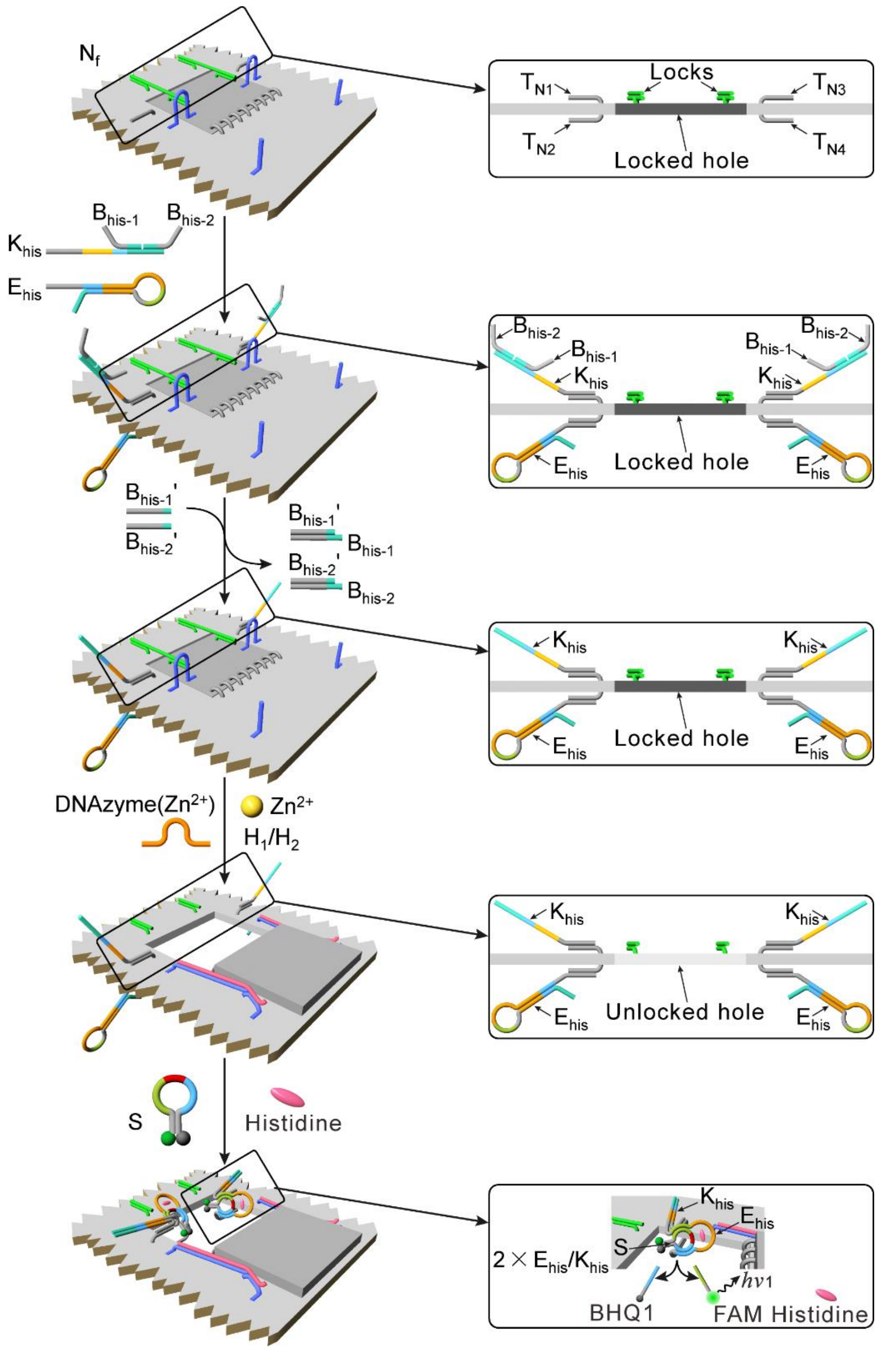

Supplementary Figure 15. Schematic of engineering of the origami tile N_f and activation of the catalysis in the confined nanocavity. The tile N_f consists of the Zn^{2+}-ion-dependent DNAzyme unlocking mechanism for the operation of the catalytic histidine-dependent DNAzyme in the confined cavity generated in the origami tile. T_{N1}/T_{N3} and T_{N2}/T_{N4} protruding tethers are linked to the opposite sides of the tile N_f. The blocked duplex $K_{his}/B_{his-1}/B_{his-2}$ and the hairpin E_{his} are hybridized with the tethers T_{N1}/T_{N3} and T_{N2}/T_{N4}, respectively. Prior to the opening of the "window" guided by the Zn^{2+}-ion-dependent DNAzyme, the protecting strands B_{his-1} and B_{his-2} are displaced by the respective counter strands B_{his-1}' and B_{his-2}', resulting in the free strand K_{his}. The Zn^{2+}-ion-dependent DNAzyme-driven opening of the nanohole in the presence of the

Zn^{2+} ions and the helper hairpins H_1/H_2 allows the subsequent interaction between the hairpin E_{his} and the strand K_{his}. As the hairpin E_{his} includes, in a caged configuration, the histidine-dependent DNAzyme, its opening by K_{his} yields the uncaged histidine-dependent DNAzyme. The resulting catalyst catalyzes the cleavage of FAM/BHQ1-functionalized substrate S, and the fluorescence of the cleaved FAM-modified fragment provides a readout signal for the catalytic process that proceeds in the nanocavity.

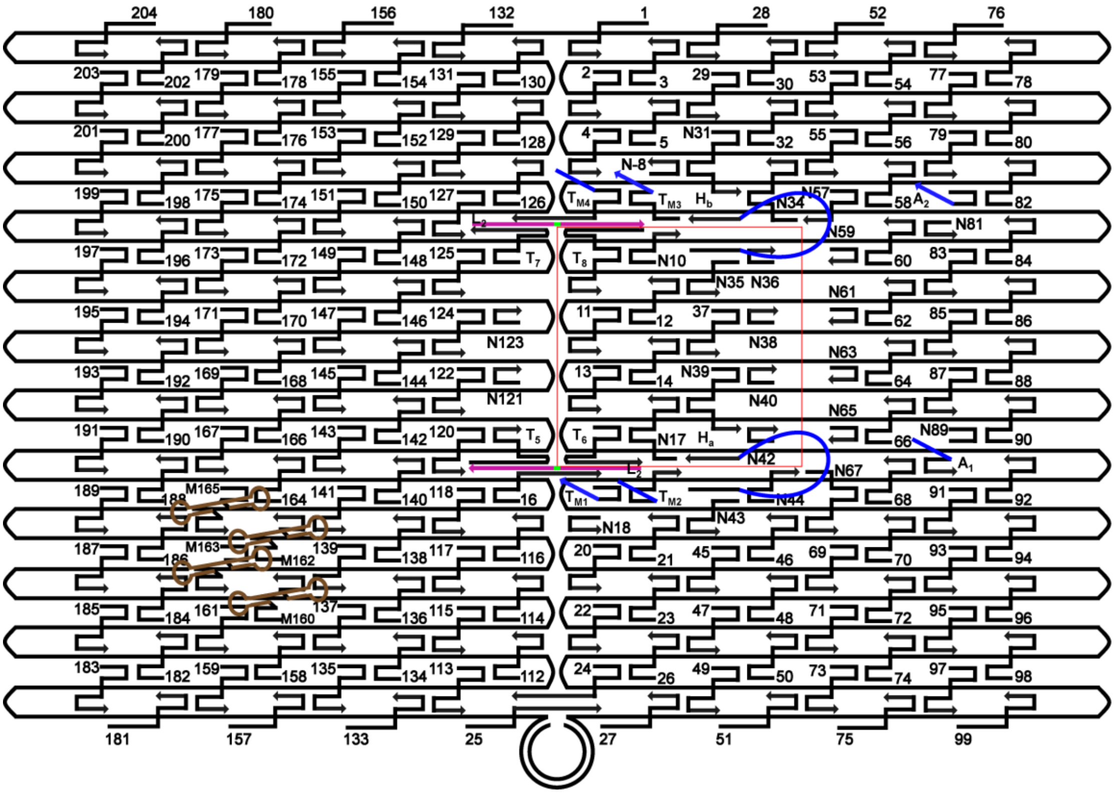

Supplementary Figure 16. Schematic of the designed origami tile M_f. The tile is functionalized with two pairs of tethers T_{M1}/T_{M3} and T_{M2}/T_{M4} that hybridize with the G_1 and G_2 strands on opposite sides of the tile raft, respectively. The tile M_f includes the Pb^{2+}-ion-dependent DNAzyme-driven unlocking apparatus in tile M, as shown in Supplementary Fig. 7.

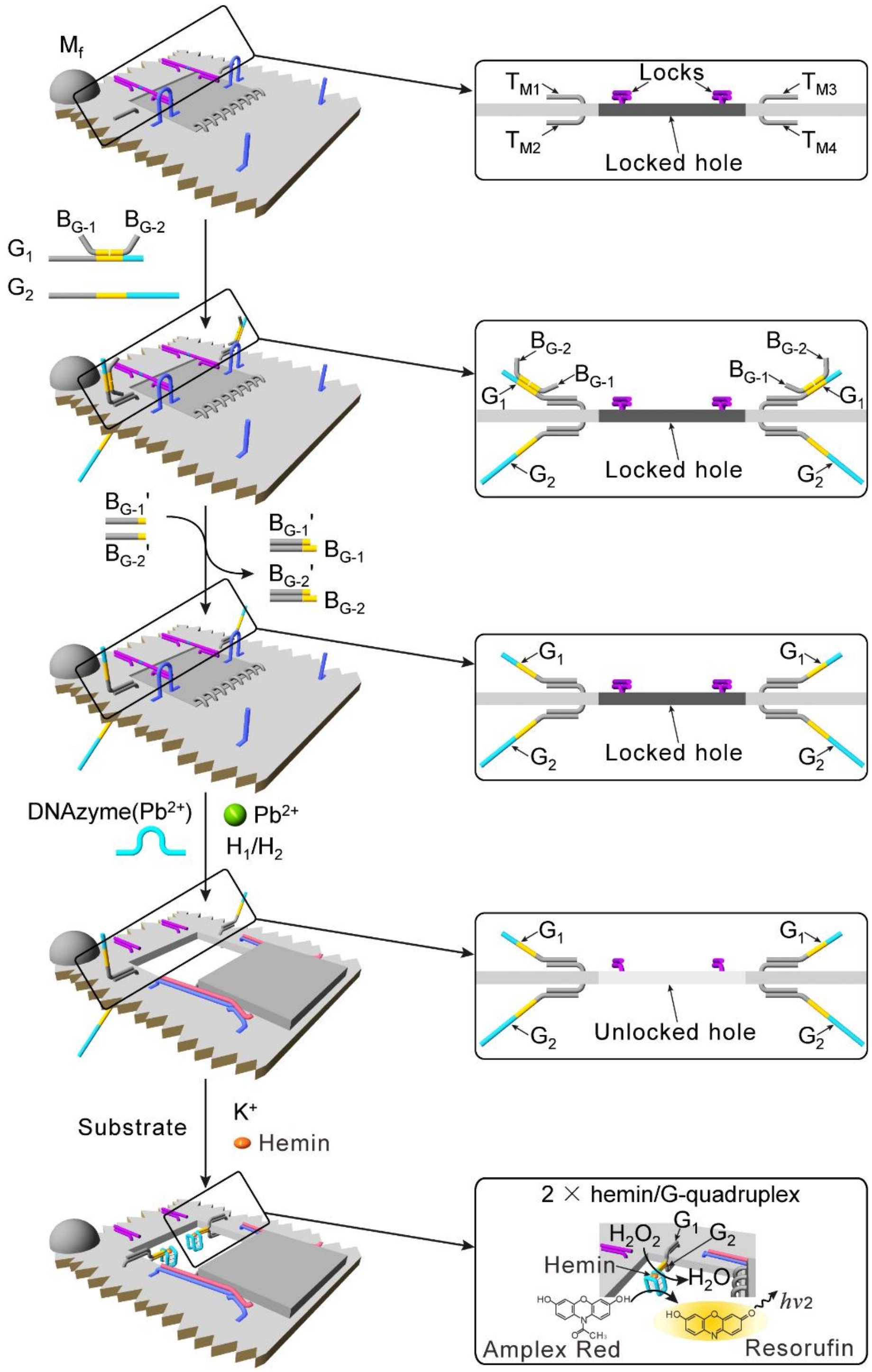

Supplementary Figure 17. Schematic of engineering of the origami tile M_f and activation of the catalysis in the confined nanocavity. The tile M_f consists of the Pb^{2+}-ion-dependent DNAzyme unlocking mechanism for the operation of the catalytic hemin/G-quadruplex DNAzyme in the confined cavity generated in the origami tile. T_{M1}/T_{M3} and T_{M2}/T_{M4} protruding tethers are linked to the opposite sides of the tile M_f. The blocked duplex $G_1/B_{G-1}/B_{G-2}$ and the strand G_2 are hybridized with the tethers T_{M1}/T_{M3} and T_{M2}/T_{M4}, respectively. Prior to the opening of the "window" guided by the Pb^{2+}-ion-dependent DNAzyme, the protecting strands B_{G-1} and B_{G-2} are displaced by the respective counter strands B_{G-1}' and B_{G-2}', resulting in the free strand G_1. The Pb^{2+}-

ion-dependent DNAzyme-driven opening of the nanohole in the presence of the Pb^{2+} ions and the helper hairpins H_1/H_2 allows the subsequent interaction between the strands G_1 and G_2. In the presence of K^+ ions and hemin, the strands G_1 and G_2 form the hemin/G-quadruplex horseradish peroxidase mimicking-DNAzymes that catalyze the H_2O_2 oxidation of Amplex Red to the fluorescent Resorufin that provides a readout signal for the catalytic process that proceeds in the nanocavity.

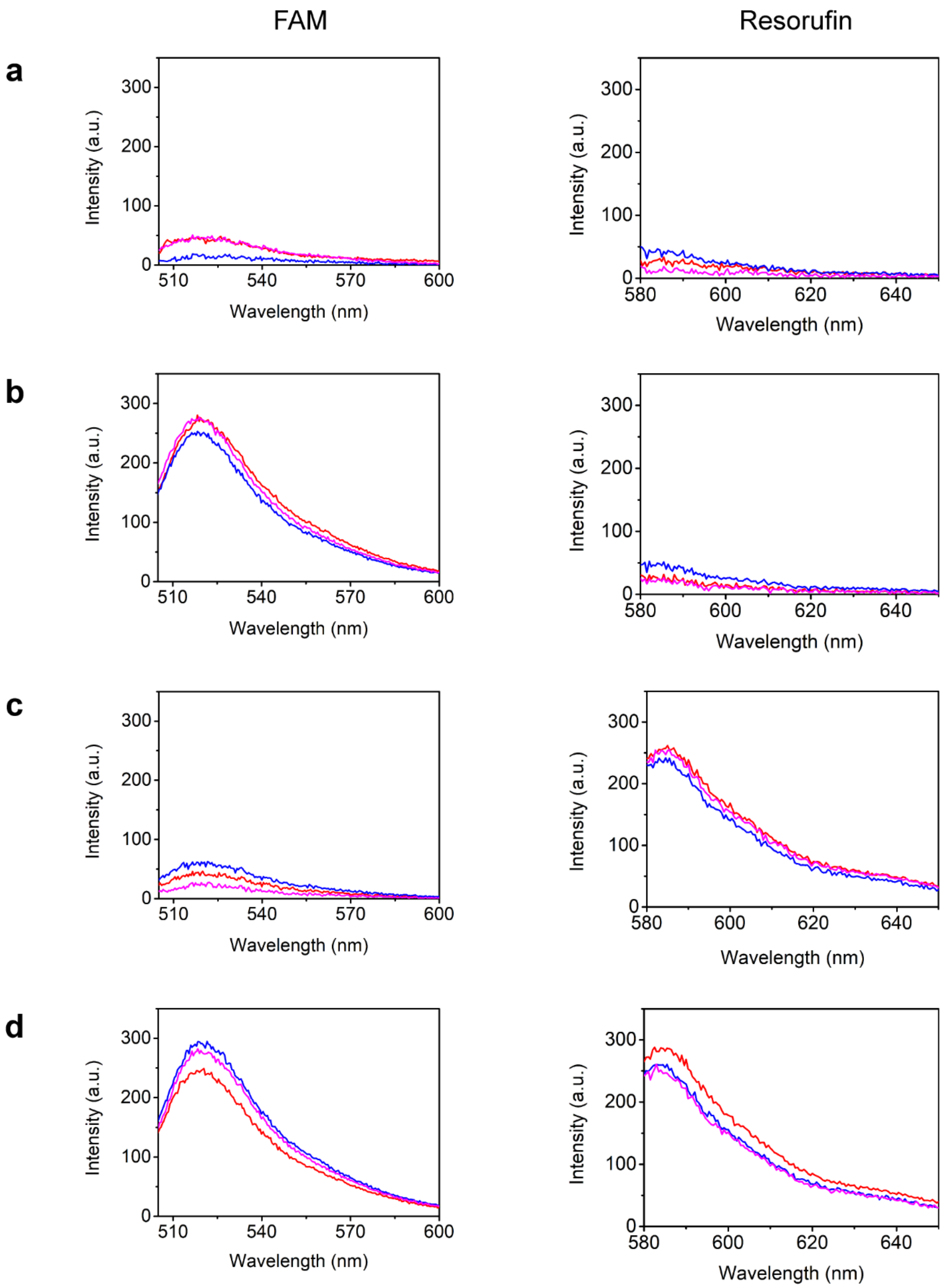

Supplementary Figure 18. Fluorescence spectra corresponding to the programmed activation of the histidine-dependent DNAzyme and/or the hemin/G-quadruplex DNAzyme in the confined nanocavities. The programmed activation of the catalytic functions of the histidine-dependent DNAzyme and/or the hemin/G-quadruplex DNAzyme were performed by unlocking the nanoholes in the tile mixture of N_f and M_f by treatment of the mixture with the Zn^{2+} ions and/or Pb^{2+} ions and the

help hairpins H_1/H_2, in the presence of Zn^{2+}-ion- and Pb^{2+}-ion-dependent DNAzyme sequences. **a**, Fluorescence spectra of FAM (left) and Resorufin (right) generated by the locked origami mixture of N_f and M_f (shown in top middle of Fig. 3). **b**, Fluorescence spectra of FAM (left) and Resorufin (right) generated upon selective unlocking of the tile N_f in the mixture with the Zn^{2+}-ion-dependent DNAzyme (cf. middle left in Fig. 3). The fluorescence spectra of FAM shows the enhanced intensity over the background signal generated in the confined cavity. **c**, Fluorescence spectra of FAM (left) and resorufin (right) upon subjecting the tile mixture of N_f and M_f to the Pb^{2+}-ion-dependent DNAzyme (cf. process shown in middle center in Fig. 3). No fluorescence change of FAM above the background signal is observed, while the fluorescence of Resorufin is enhanced as compared to the background fluorescence signal. **d**, Fluorescence spectra of FAM (left) and Resorufin (right) generated upon subjecting the tile mixture of N_f and M_f to the Zn^{2+}-ion- and Pb^{2+}-ion-dependent DNAzymes (cf. process shown in middle right of Fig. 3). The fluorescence of FAM and the fluorescence of Resorufin are intensified as compared to the background signals, consistent with the activation of the two DNAzymes in the nanocavities. Each of the fluorescence spectra of the respective system shows the spectra of three different experiments (N = 3).

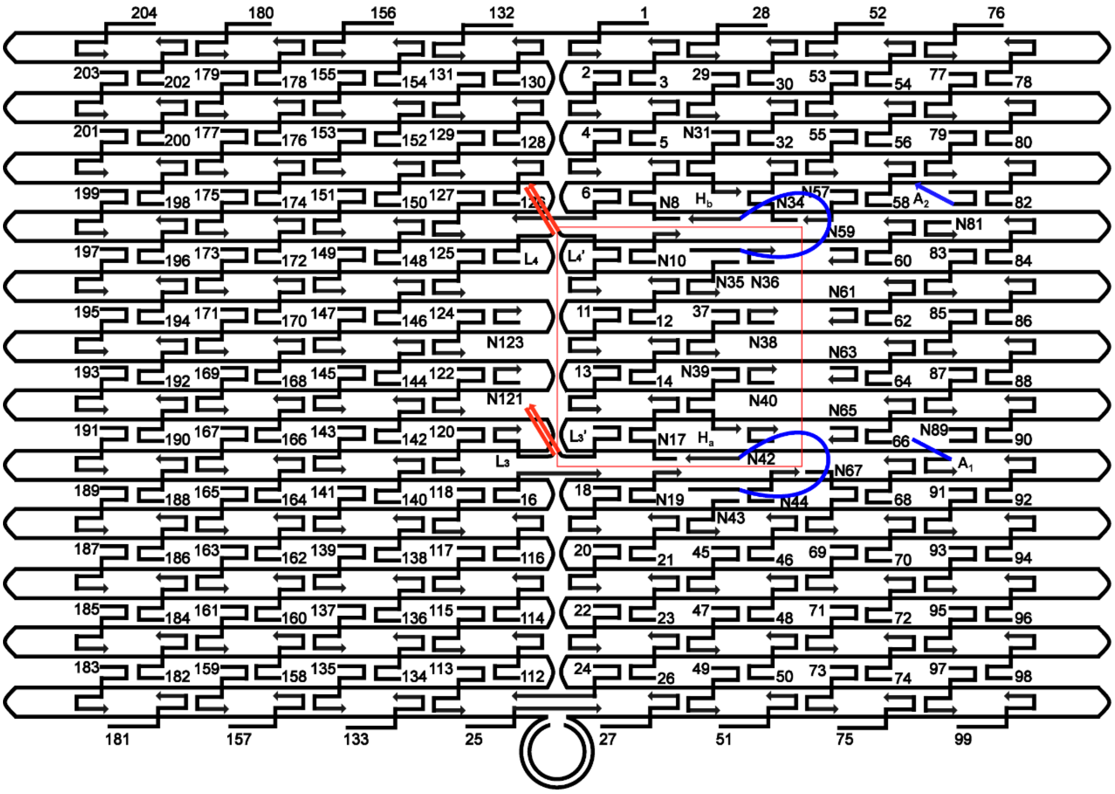

Supplementary Figure 19. Schematic of the designed origami tile O. The photochemical switchable locks (L_3/L_3', L_4/L_4') are shown in red double lines, the handles (H_a, H_b) are blue loops and the anchoring tethers (A_1, A_2) are short blue lines. The red square shows the area of the designed “window” in the origami tile O.

The left side of the designed “window” is locked to the origami tile scaffold using linker strands (L_3/L_3', L_4/L_4', in red color) that extend from the staple strands on the “window” and the origami tile scaffold[1]. L_3' and L_4 are modified with azobenzene units that response to UV (λ = 365 nm) and visible light (λ > 420 nm) to unlock or lock the “window”, respectively. The handles (blue loop) and the anchoring footholds are same as that in the origami tile N in Supplementary Fig. 1. The staples on the left and right edges are removed to avoid the aggregation of the origami tiles[1].

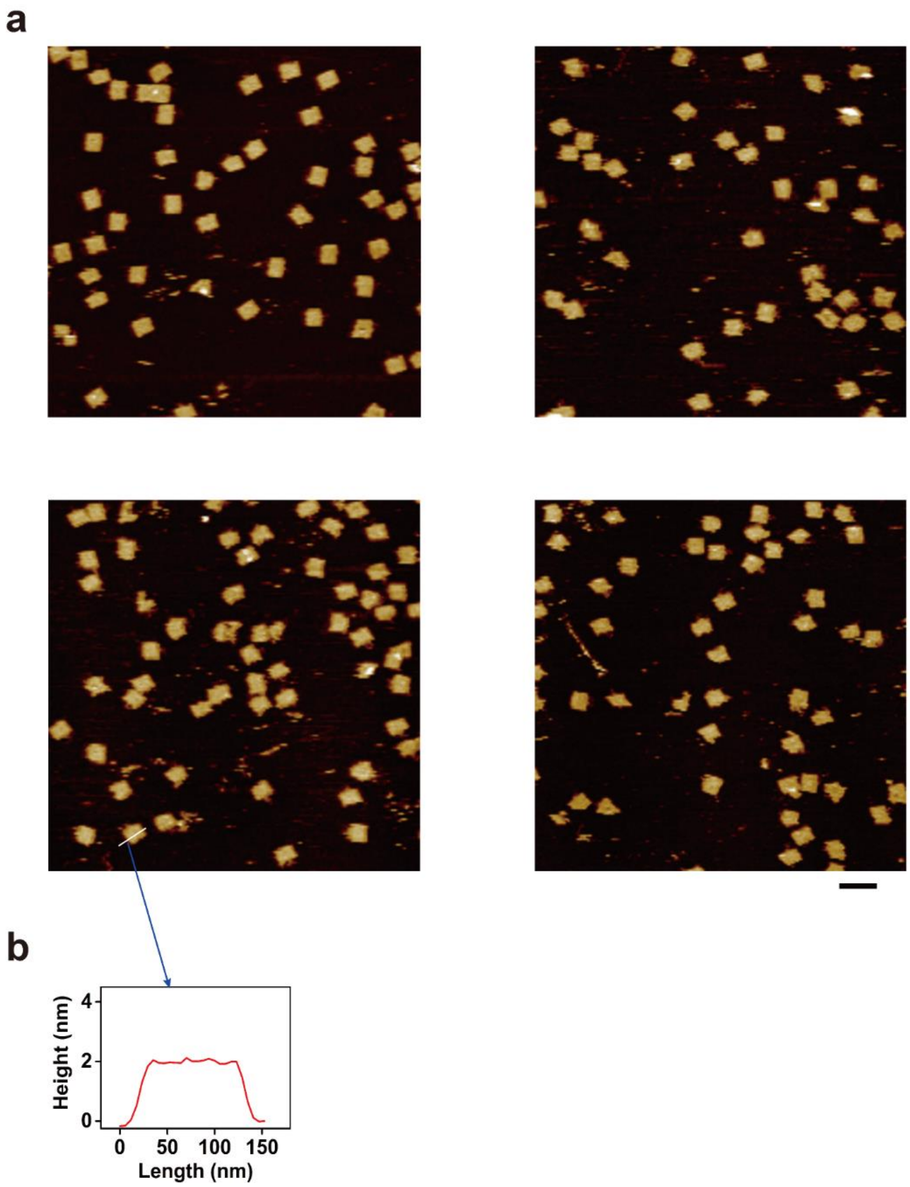

Supplementary Figure 20. AFM measurements of the locked origami tiles O. a, Four AFM images of the initial locked origami tiles O before the treatment with UV light irradiation and the helper hairpins H_3/H_4. **b,** cross-section analysis of the tile O. Source data are provided as a Source Data file. Scale bar: 200 nm.

Supplementary Table 10. Statistical analysis of the yields of the unlocked and locked origami tiles O before the treatment with UV light irradiation and the helper hairpins H_3/H_4. The analysis is performed on the images shown in Supplementary Fig. 20a.

**Statistical analysis**[a]		**Unlocked**	**Locked**	**Incomplete structure**
**1**	Count	0	47	1
	Yield (%)	0	97.9	2.1
**2**	Count	0	37	1
	Yield (%)	0	97.4	2.6
**3**	Count	0	41	1
	Yield (%)	0	97.6	2.4
**4**	Count	0	51	2
	Yield (%)	0	96.2	3.8

a. The statistical analysis of the locked origami tiles involved the identification of intact origami structures. Imperfect tiles were identified as damaged origami scaffolds.

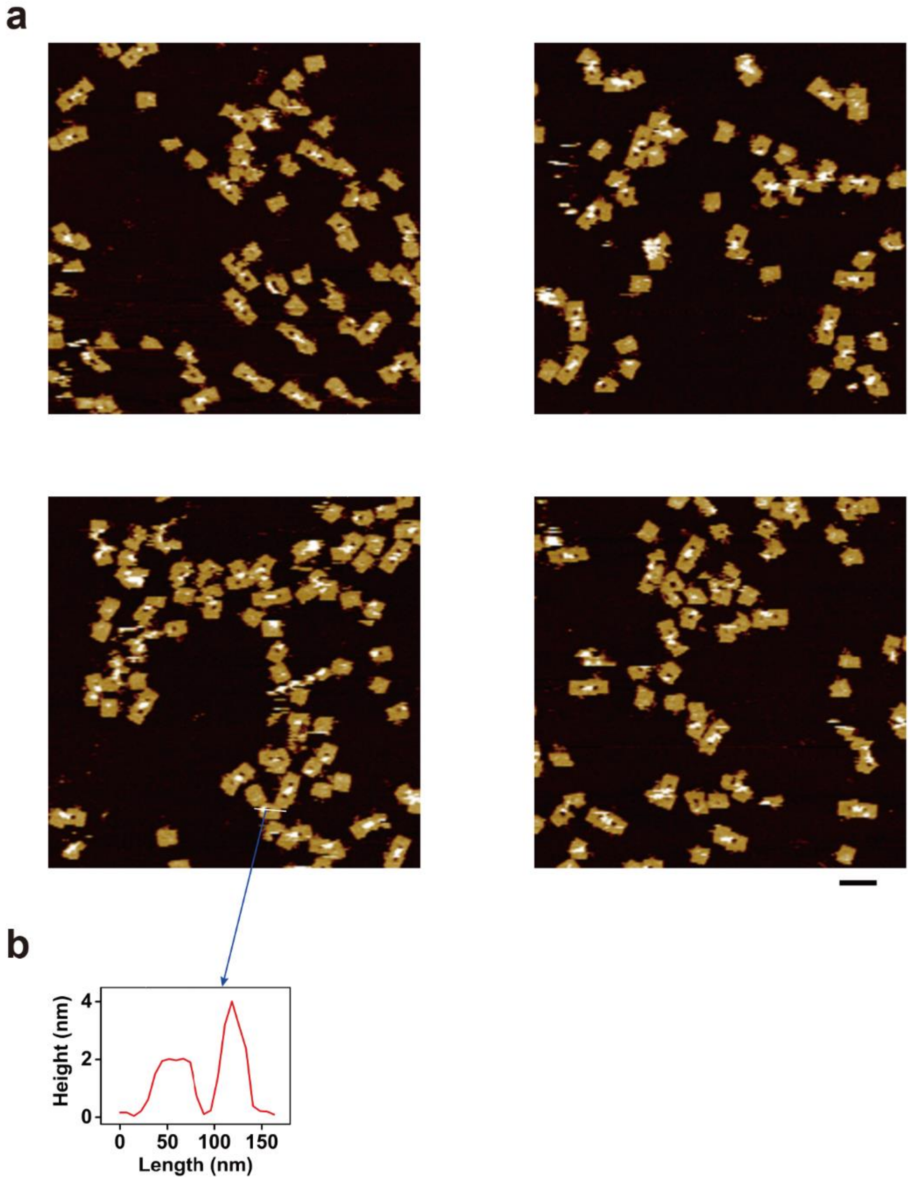

Supplementary Figure 21. AFM measurements of the unlocked origami tiles O. a, Four AFM images of the unlocked origami tiles O upon the treatment of the tiles O with UV light irradiation (λ = 365 nm) and the helper hairpins H_3/H_4 (first cycle). **b,** cross-section analysis of the tile O confirming the formation of the nanohole. Source data are provided as a Source Data file. Scale bar: 200 nm.

Supplementary Table 11. Statistical analysis of the yields of the unlocked and locked origami tiles O in the unlocked state (II) after the treatment with UV light irradiation (λ = 365 nm) and the helper hairpins H_3/H_4 (first cycle). The analysis is performed on the images shown in Supplementary Fig. 21a.

**Statistical analysis[a]**		**Unlocked**	**Locked**	**Incomplete structure**
**1**	Count	38	6	5
	Yield (%)	77.6	12.2	10.2
**2**	Count	38	13	2
	Yield (%)	71.7	24.5	3.8
**3**	Count	39	10	4
	Yield (%)	73.6	18.9	7.5
**4**	Count	48	14	2
	Yield (%)	75	21.9	3.1

a. The statistical analysis of the unlocked origami tiles involved the identification of intact origami structures that included open cavities on the tiles. Locked structures were identified as tiles without identified cavity. Imperfect tiles were identified as damaged origami scaffolds.

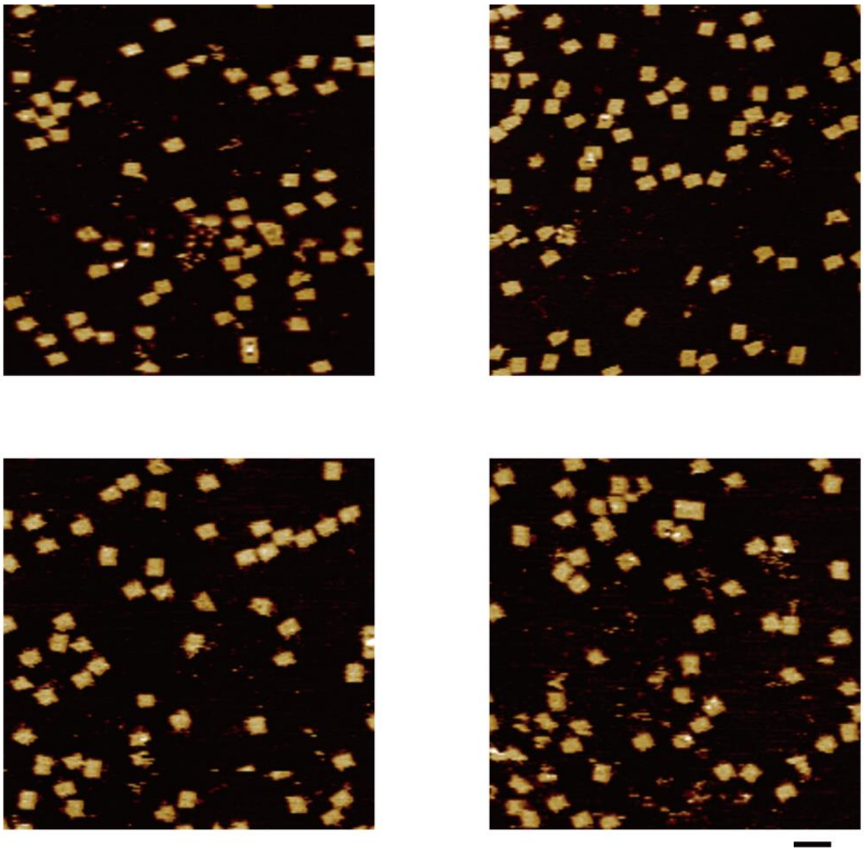

Supplementary Figure 22. AFM measurements of the regeneration of the locked origami tiles O. Four AFM images of the regenerated and locked origami tiles O upon the treatment of the unlocked tiles with anti-helper strands (H_{3a}'/H_{3b}' and H_{4a}'/H_{4b}') and visible light irradiation (λ > 420 nm). Scale bar: 200 nm.

Supplementary Table 12. Statistical analysis of the yields of the unlocked and locked origami tiles O upon the treatment of the unlocked tiles with anti-helper strands (H_{3a}'/H_{3b}' and H_{4a}'/H_{4b}') and visible light irradiation (λ > 420 nm). The analysis is performed on the images shown in Supplementary Fig. 22.

**Statistical analysis[a]**		**Unlocked**	**Locked**	**Incomplete structure**
**1**	Count	3	46	2
	Yield (%)	5.9	90.2	3.9
**2**	Count	3	52	2
	Yield (%)	5.3	91.2	3.5
**3**	Count	2	42	2
	Yield (%)	4.3	91.3	4.3
**4**	Count	2	37	3
	Yield (%)	4.8	88.1	7.1

a. The statistical analysis of the unlocked and locked origami tiles involved the identification of intact origami structures. Imperfect tiles were identified as damaged origami scaffolds.

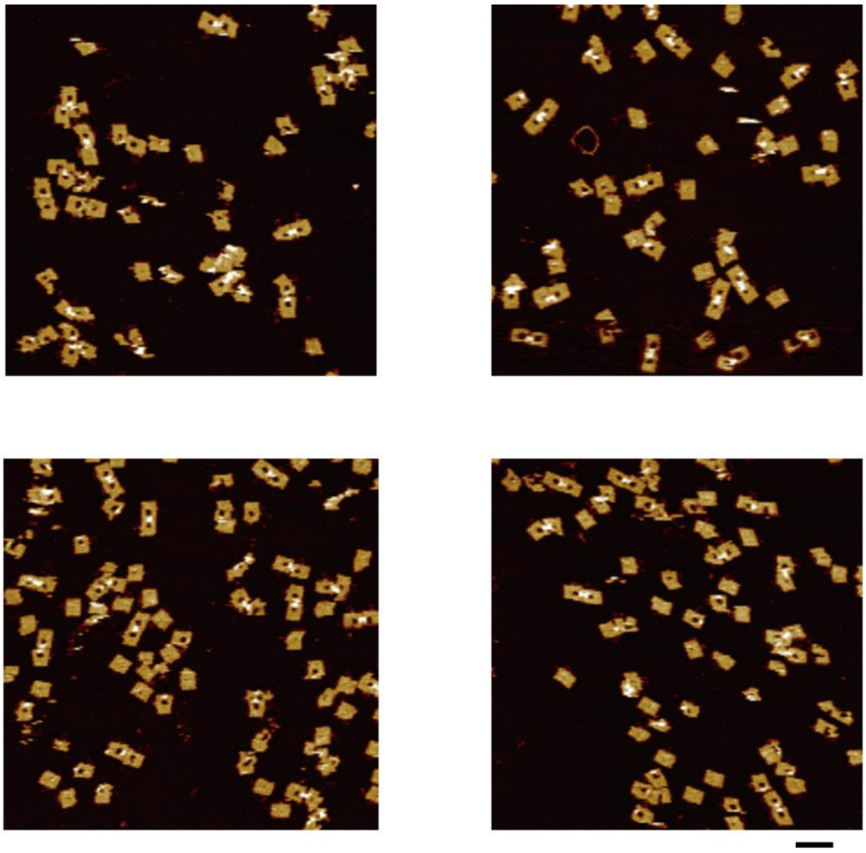

Supplementary Figure 23. AFM measurements of the unlocked origami tiles O (second cycle). Four AFM images of the unlocked origami tiles O after the treatment with UV light irradiation (λ = 365 nm) and the helper hairpins H_3/H_4 (second cycle). Scale bar: 200 nm.

Supplementary Table 13. Statistical analysis of the yields of the unlocked and locked origami tiles O after the treatment with UV light irradiation (λ = 365 nm) and the helper hairpins H_3/H_4 (second cycle). The analysis is performed on the images shown in Supplementary Fig. 23.

**Statistical analysis**[a]		**Unlocked**	**Locked**	**Incomplete structure**
**1**	Count	32	10	3
	Yield (%)	71.1	22.2	6.7
**2**	Count	29	6	3
	Yield (%)	76.3	15.8	7.9
**3**	Count	39	8	5
	Yield (%)	75	15.4	9.6
**4**	Count	40	16	5
	Yield (%)	65.6	26.2	8.2

a. The statistical analysis of the unlocked and locked origami tiles involved the identification of intact origami structures. Imperfect tiles were identified as damaged origami scaffolds.

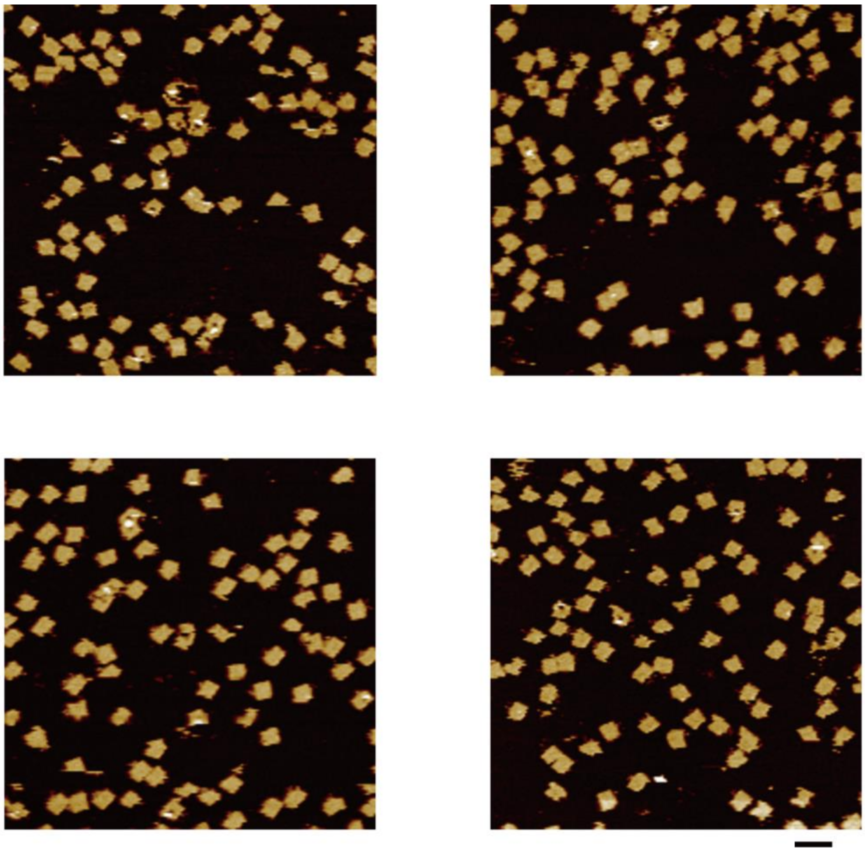

Supplementary Figure 24. AFM measurements of the locked origami tile O (second cycle). Four AFM images of the regenerated and locked origami tiles after the treatment of the unlocked tiles with anti-helper strands (H_{3a}'/H_{3b}' and H_{4a}'/H_{4b}') and visible light irradiation (λ > 420 nm) (second cycle). Scale bar: 200 nm.

Supplementary Table 14. Statistical analysis of the yields of the unlocked and locked origami tiles O after the treatment of the unlocked tiles with anti-helper strands (H_{3a}'/H_{3b}' and H_{4a}'/H_{4b}') and visible light irradiation (λ > 420 nm) (second cycle). The analysis is performed on the images shown in Supplementary Fig. 24.

Statistical analysis[a]		Unlocked	Locked	Incomplete structure
**1**	Count	5	59	5
	Yield (%)	7.2	85.5	7.2
**2**	Count	7	58	6
	Yield (%)	9.9	81.7	8.5
**3**	Count	5	46	3
	Yield (%)	9.3	85.2	5.6
**4**	Count	7	58	3
	Yield (%)	10.3	85.3	4.4

a. The statistical analysis of the unlocked and locked origami tiles involved the identification of intact origami structures. Imperfect tiles were identified as damaged origami scaffolds.

Control experiments for the light-induced unlocking of the origami tile O

Supplementary Fig. 25 shows the AFM images of origami tiles subjected to the hairpins H_3/H_4 in the absence of light. No formation of nanoholes is detected implying that the irradiation of the tiles O is essential to generate the nanoholes. Supplementary Fig. 26 shows the AFM images of the origami tiles O subjected to the photoinduced isomerization of the *trans*-azobenzene to *cis*-azobenzene units, leading to the unlocking of the locks, in the absence of the hairpins H_3/H_4. Very low yield (< 8%) of the hole-containing origami tiles is observed, despite the cleavage of locks. These results indicate that the H_3/H_4-driven mechanical stretching of the "window" domain through the handle units is essential to produce the high-yield nanopore-containing tiles (74%). Indeed, treatment of the light-induced origami mixture shown in Supplementary Fig. 26 with hairpins H_3/H_4 leads to the stretching of the "window" domains and to the formation of the high-yield nanopore-containing tiles.

Furthermore, the AFM image of the nanohole-containing tiles shown in Fig. 4b(II), text, reveals some dimer structures of the origami tiles. These dimer structures are attributed to inter-origami tiles interaction upon deposition on the mica surface. The nanohole-containing tiles exist, however, as monomers in solution. This was confirmed by electrophoretic measurement shown in Supplementary Fig. 27[2,3].

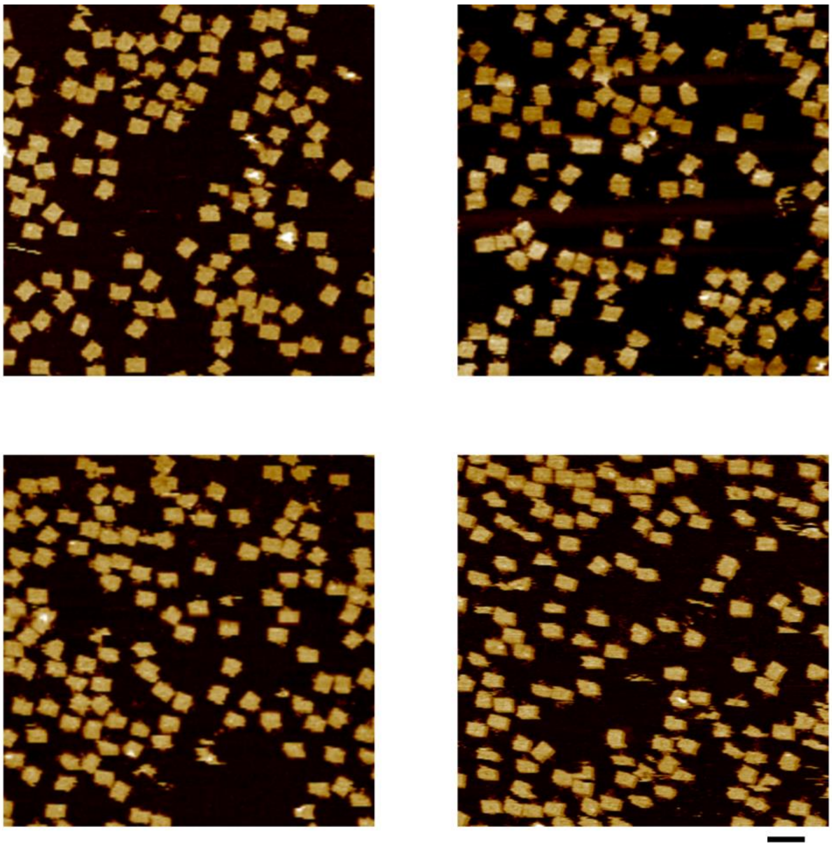

Supplementary Figure 25. AFM images of the control experiment examining the unlocking of the origami tiles O in the presence of the helper hairpins H_3/H_4 and in the absence of UV irradiation (λ = 365 nm). No tiles with nanoholes are observed, implying that the UV irradiation is essential to unlock the origami tiles to yield the nanoholes. Scale bar: 200 nm.

Supplementary Table 15. Statistical analysis of the yields of the unlocked and locked origami tiles O in the presence of the helper hairpins H_3/H_4 and in the absence of UV irradiation (λ = 365 nm). The analysis is performed on the images shown in Supplementary Fig. 25.

**Statistical analysis[a]**		**Unlocked**	**Locked**	**Incomplete structure**
**1**	Count	0	93	9
	Yield (%)	0	91.2	8.8
**2**	Count	0	94	8
	Yield (%)	0	92.2	7.8
**3**	Count	0	103	8
	Yield (%)	0	92.8	7.2
**4**	Count	0	97	21
	Yield (%)	0	82.2	17.8

a. The statistical analysis of the locked origami tiles involved the identification of intact origami structures. Imperfect tiles were identified as damaged origami scaffolds.

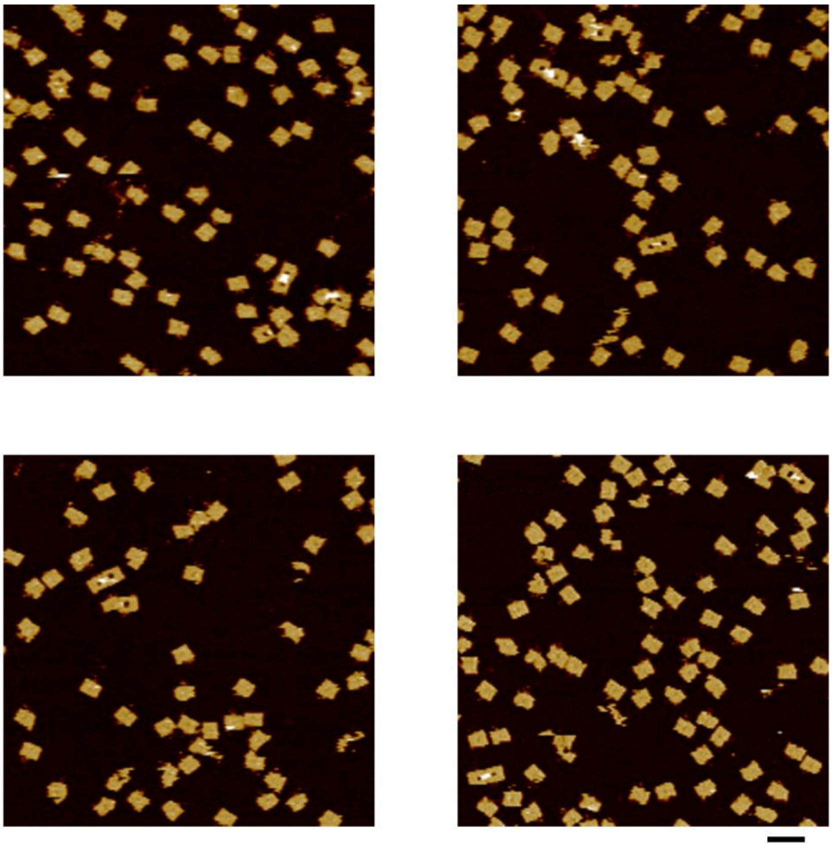

Supplementary Figure 26. AFM images of the control experiment examining the photo-driven unlocking of the origami tiles O in the absence of the helper hairpins H_3/H_4. Only few origami tiles show open nanoholes. The results imply that the unlocked “window” exists in a flexible configuration that retains the nanohole closed, in the absence of the hairpin helper units H_3/H_4 that stretch the “window” to a rigid configuration by the hairpins/handles/anchor site units. Scale bar: 200 nm.

Supplementary Table 16. Statistical analysis of the yields of the unlocked and locked origami tiles O in the presence of UV irradiation (λ = 365 nm) and in the absence of the helper hairpins H_3/H_4. The analysis is performed on the images shown in Supplementary Fig. 26.

**Statistical analysis[a]**		**Unlocked**	**Locked**	**Incomplete structure**
**1**	Count	5	51	2
	Yield (%)	8.6	87.9	3.4
**2**	Count	5	51	3
	Yield (%)	8.5	86.4	5.1
**3**	Count	3	45	4
	Yield (%)	5.8	86.5	7.7
**4**	Count	4	63	4
	Yield (%)	5.6	88.7	5.6

a. The statistical analysis of the unlocked and locked origami tiles involved the identification of intact origami structures. Imperfect tiles were identified as damaged origami scaffolds.

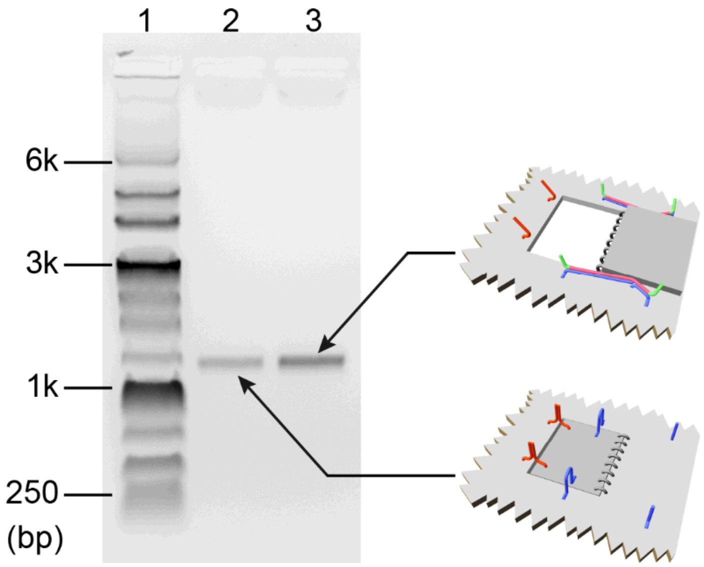

Supplementary Figure 27. Electrophoresis gel result and bands corresponding to the locked tile and unlocked tile solutions (lane 2 and 3, respectively), indicating that the tiles exist in solutions as single tiles, and the small population of the dimer constitutions observed in the AFM images on the mica originate from the surface adhesion phenomena. Lane 1 is the reference ladder (kilobase) to indicate the relative position of the migration band of the origami tile structures[4]. Source data is provided as a Source Data file.

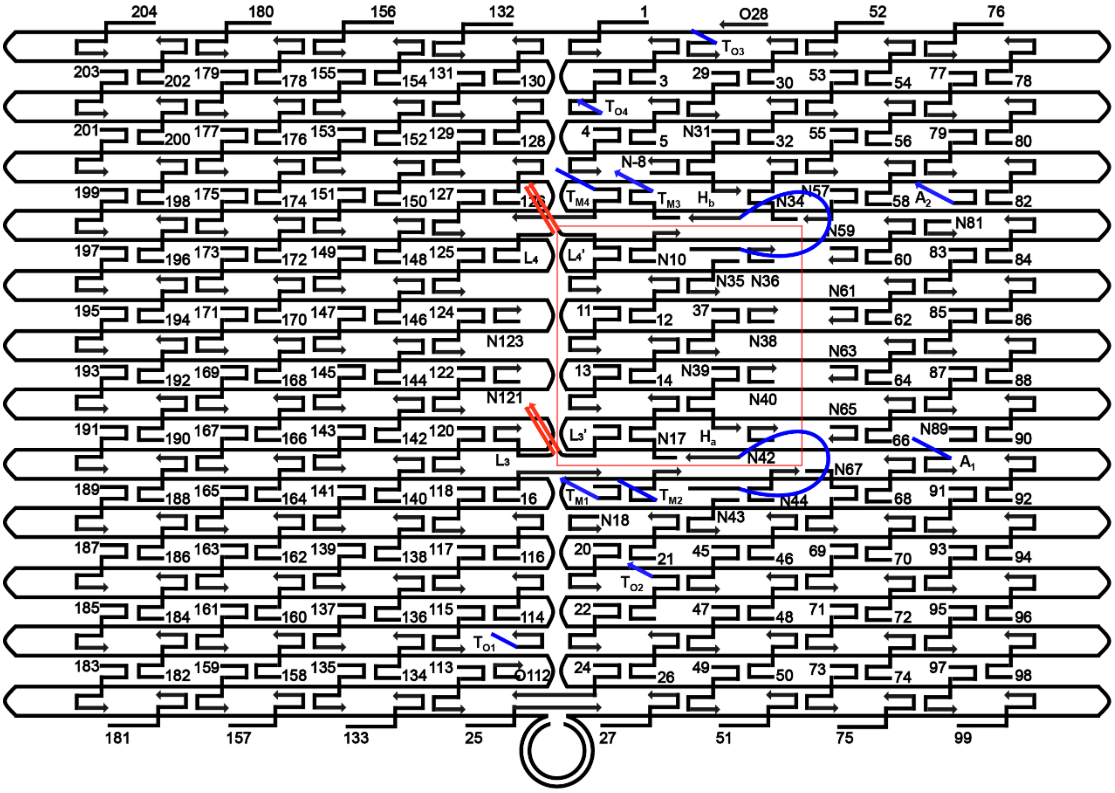

Supplementary Figure 28. Schematic of the design of the origami tile O_f. The tile is functionalized with four pairs of tethers that hybridize with the G_{1x} and G_2 strands and separate the duplexes of the two strands during the switching process on opposite sides of the tile scaffold. The tile O_f includes the photochemical unlocking apparatus in tile O, as shown in Supplementary Fig. 19.

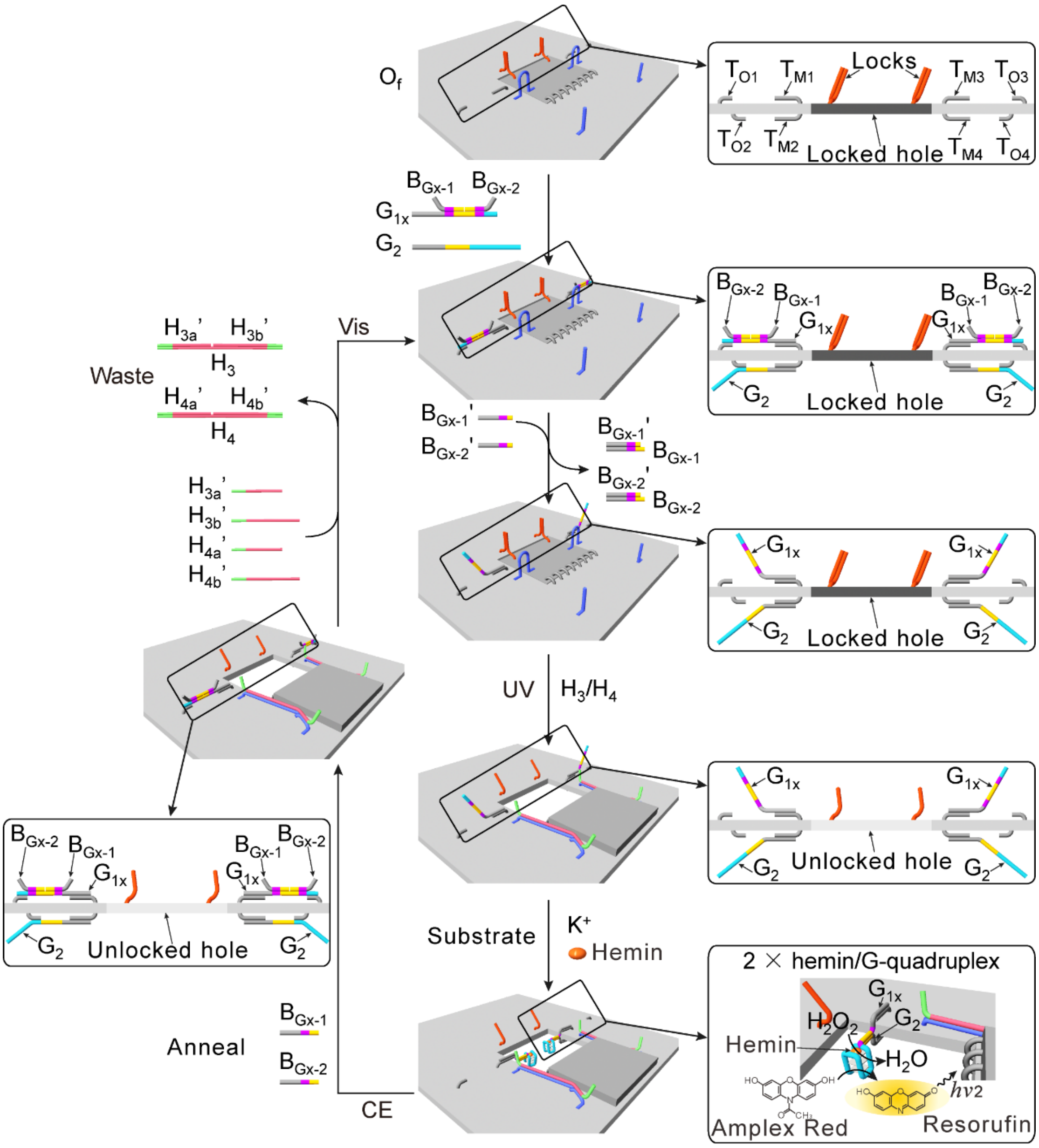

Supplementary Figure 29. Schematic of engineering of the origami tile O_f and the photo-switchable catalysis of the hemin/G-quadruplex DNAzyem in the nanocavity. The tile O_f includes the photochemically-driven unlocking apparatus (discussed in Fig. 4 and text). Protruding tethers T_{M1}/T_{M3} and T_{M2}/T_{M4} are designed on the opposite sides of the origami raft. The duplex G_{1x}/B_{Gx-1}/B_{Gx-2} and the strand G_2 are hybridized with the tethers T_{M1}/T_{M3} and T_{M2}/T_{M4}, respectively. Prior to the photochemical unlocking of the tile, G_{1x}/B_{Gx-1}/B_{Gx-2} are unblocked by the strand displacement process, using appropriate anti-blockers (B_{Gx-1}'/B_{Gx-2}'). The deblocked strand G_{1x} and G2 correspond to G-quadruplex subunits. The photochemically induced unlocking of the origami tile O_f, in the presence of the helper hairpins H_3 and H_4 leads to the formation of the nanohole. In the presence of K^+ ions and hemin, the hemin/G-quadruplex DNAzymes that catalyze the H_2O_2 oxidation of Amplex Red to the fluorescent Resorufin are formed in the confined nanocavity. Treatment of the catalytic system with 18-crown-6-ether(CE) and the blockers B_{Gx-1} and B_{Gx-2} separates the G-

quadruplex units and the subsequent photo-isomerization of the *cis*-azobenzene units in the presence of the respective counter helper units (H_{3a}'/H_{3b}'/H_{4a}'/H_{4b}'), leads to the closure of the nanohole (cf. Fig. 4 and accompanying discussion in the text). The scheme represents the mechanistic path for the cyclic photo-switching of the activity of the hemin/G-quadruplex in the confined nanocavity associated with the origami tile. The reversible switching of the fluorescence of Resorufin provides the readout signal for the “ON”/“OFF” photochemical switching of the catalytic functions of the system.

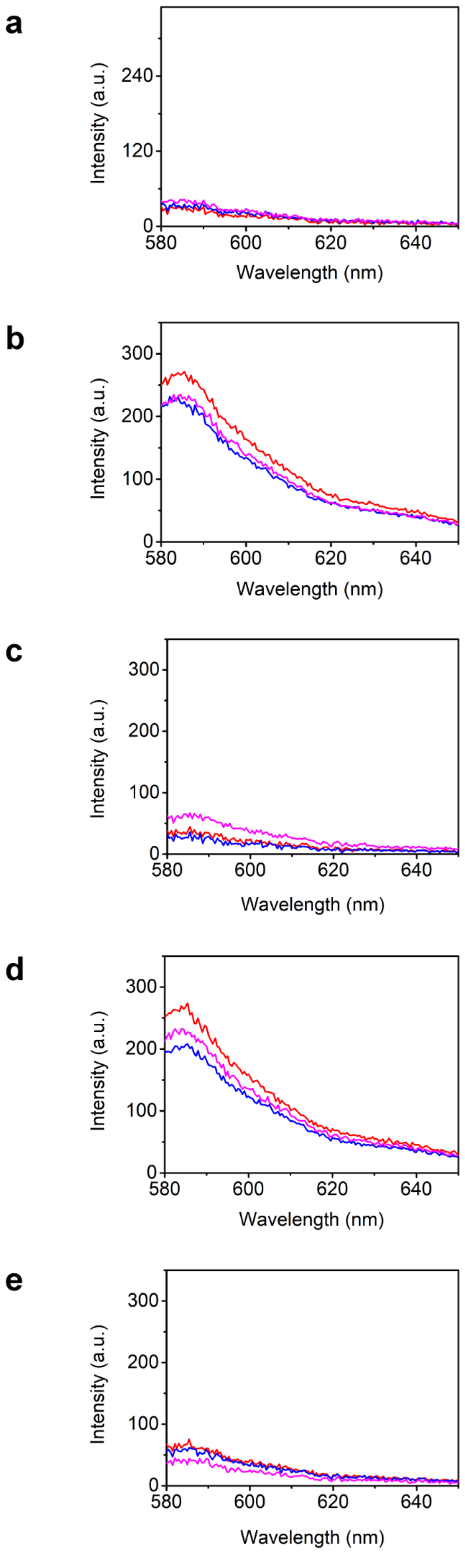

Supplementary Figure 30. Fluorescence spectra corresponding to the cyclic photo-switching activation of the hemin/G-quadruplex DNAzyme upon the photo-driven unlocking and locking of the nanoholes in the origami O_f. a, Fluorescence spectra of Resorufin generated by the locked origami O_f. **b**, Fluorescence spectra of Resorufin generated upon unlocking of the origami tile O_f with the UV irradiation (λ = 365 nm) and the helper hairpins H_3/H_4 (cf. process shown in Supplementary Fig. 29). The

fluorescence spectra of Resorufin show the enhanced intensity over the background signal generated in the confined cavity (cf. first cycle in Fig. 4e). **c**, Fluorescence spectra of Resorufin upon subjecting the unlocked origami to the crown ether (CE), blockers B_{Gx-1}/B_{Gx-2}, anti-helpers $H_{3a}'/H_{3b}'/H_{4a}'/H_{4b}'$ and visible light (λ > 420 nm) (cf. process shown in Supplementary Fig. 29). No fluorescence changes of Resorufin above the background signal are observed (cf. first cycle in Fig. 4e). **d**, Fluorescence spectra of Resorufin generated upon subjecting the locked origami tile O_f to the anti-blockers (B_{Gx-1}'/B_{Gx-2}'), UV irradiation (λ = 365 nm) and helper hairpins H_3/H_4 (cf. process shown in Supplementary Fig. 29). The fluorescence intensities of Resorufin are enhanced as compared to the background signal, consistent with the activation of the hemin/G-quadruplex DNAzyme in the nanocavity (cf. second cycle in Fig. 4e). **e**, Fluorescence spectra of Resorufin upon subjecting the unlocked origami tile O_f to the CE, blockers (B_{Gx-1}/B_{Gx-2}), anti-helpers $H_{3a}'/H_{3b}'/H_{4a}'/H_{4b}'$ and visible light irradiation (λ > 420 nm) (cf. process shown in Supplementary Fig. 29). No fluorescence changes of Resorufin above the background signal are observed (cf. second cycle in Fig. 4e). Each of the fluorescence spectra of the respective systems shows the spectra of three different experiments (N = 3).

Supplementary Table 17. DNA sequences of origami tile N.

T_1	CATGGTAGGTAGTGCTCATCTTTGGCCGGAGACAGTCATTCAAA A
T_2	GCGTTATAGAAAAAGCCTGTTTAGTTTGATGTCATGGACTCTGT CTC
T_3	CATAACCCGAGGCATAGTAAGAGCTTTGATGTCATGGACTCTGT CTC
T_4	CATGGTAGGTAGTGCTCATCTTTTTTAAGAAAAGTAATATCTTAC CGAAGCCCTTCCAGAG
N123	TTTTAATTGCCCGAAA
N121	TTTCATTTGGTCAATA
N8	TTCAACCGATTGAGGGTAAAGGTGAATTATCAATCACCGG
N10	GCAATAGCGCAGATAGCCGA
N17	GGAATCATAATTACTACAAATTCTTACCAGTAATCCCATC
N19	TAGAATCCCTGAGAAGAGTC
N31	GCCACCACTCTTTTCATAATCAAACCGTCACCGACTTGAG
N35	ATAAGAGCAAGAAACAACAGCCAT
N39	GGTATTAAGAACAAGA AAAATAATTAAAGCCAACGCTCAA
N43	CGTCGCTATTAATTAACGGATTCG
N34	GGTTTACCAGCGCCAACCATTTGGGAATTAGATTATTAGC
N36	GCCCAATACCGAGGAA
N38	TATTTTGCTCCCAATC
N40	TAAGTCCTACCAAGTA
N42	AGGCGTTACAGTAGGG
N44	CTGTAAATCATAGGTCTGAGAGAC
N57	AATCACCAAATAGAAAATTCATAT
N59	ATACCCAAGATAACCC
N61	TTTTGTTTAAGCCTTA
N63	CAAGCAAGACGCGCCT
N65	CATATTTAGAAATACC
N67	TACCTTTTTAACCTCCATATGTGAGTGAATAAACAAAATC
N81	GAATAAGTAAGACTCC
N89	AGAGGCATAATTTTCAT
H_a	AATAGTGAATTTATCAAATTTTAAATTCAACTTTTTTACCATTTTA ATTTTAACCTTTTCCTTTTAATAAGAATAAACACC
H_b	ACAAAGTTACCAGAAGGATTTTCATCCATTCATTTTCATTACTTT AATTCCTTAATTCTTTTCTTAGACAAAAGGGCGACA
A_1	TTGGAAAAGGTTTTTTCTTCTGACTATAACTA
A_2	CCGGAAACACACCACGTTTTTGAATGGATGTT
$A_{1\text{-}C5}$	Cy5-TTGGAAAAGGTTTTTTCTTCTGACTATAACTA
$H_{a\text{-}C3}$	AATAGTGAATTTATCAAATTTTAAATTCAACTTTTTTACCATTTTA ATTTTAACCTTTTCCTTTT[Cy3]AATAAGAATAAACACC

Supplementary Table 18. DNA sequences of origami tile M.

T_5	GAGTCGATCTTCAGTGACTGTTTGGCCGGAGACAGTCATTCAAAA
T_6	GCGTTATAGAAAAAGCCTGTTTAGTTTCTGGATTGTCTACTCCTTCG
T_7	CATAACCCGAGGCATAGTAAGAGCTTTCTGGATTGTCTACTCCTTCG
T_8	GAGTCGATCTTCAGTGACTGTTTTTTAAGAAAAGTAATATCTTACCGAAGCCCTTCCAGAG
M160	ACTGCCCGCCGAGCTCATGAATCCTTTTGGATTCATCAAGTGCTTTTTAGCACTTGGAATTCGTTATTACGC
M162	CAGCTGGCGGACGACGATGAATCCTTTTGGATTCATCAAGTGCTTTTTAGCACTTGACAGTATCGTAGCCAG
M163	GTTTGAGGGAAAGGGGATGAATCCTTTTGGATTCATCAAGTGCTTTTTAGCACTTGGATGTGCTAGAGGATC
M165	AGAAAAGCAACATTAAATGAATCCTTTTGGATTCATCAAGTGCTTTTTAGCACTTGATGTGAGCATCTGCCA

Supplementary Table 19. DNA sequences of origami tile N_f.

T_{N1}	TTAAGACGTTGAAAACTTTTGATGGTCAGGTTGTCCTG
T_{N2}	GTCACTGGTAGAGGTGTCTTTTTAGAATCCCTGAGAAGAGTC
T_{N3}	TTCAACCGATTGAGGGTAAAGGTGTTTTGATGGTCAGGTTGTCCTG
T_{N4}	GTCACTGGTAGAGGTGTCTTTTTTATTCATAGGGAAGGTAAATATTCATTCAGT
N18	ATAGCGATAACAGTAC
N-8	AATTATCAATCACCGG

Supplementary Table 20. DNA sequences of origami tile M_f.

T_{M1}	TTAAGACGTTGAAAACTTTTGAGCTGTATCACACGTAC
T_{M2}	GTAGTCCAACCTGTCTACTTTTTAGAATCCCTGAGAAGAGTC
T_{M3}	TTCAACCGATTGAGGGTAAAGGTGTTTTGAGCTGTATCACACGTAC
T_{M4}	GTAGTCCAACCTGTCTACTTTTTTATTCATAGGGAAGGTAAATATTCATTCAGT

Supplementary Table 21. DNA sequences of origami tile O

L_3	GTAGACAGTGTTTGGCCGGAGACAGTCATTCAAAA
L_3'	GCGTTATAGAAAAAGCCTGTTTAGTTTCA*Azo*CT*Azo*GT*Azo*CT*Azo* AC
L_4	CATAACCCGAGGCATAGTAAGAGCTTTGA*Azo*GT*Azo*CA*Azo*TG*Azo* TG
L_4'	CACATGACTCTTTTTTAAGAAAAGTAATATCTTACCGAAGCCCT TCCAGAG

Supplementary Table 22. DNA sequences of origami tile O_f.

T_{O1}	GTTACCCATTTCTGTTTGAAGCCGGAA
T_{O2}	GGATTTAGCGTATTAAATCCTTTGTTTTCAGGTTTACCCAGTC
T_{O3}	GTTACCCATTTTCATTTTCCTATTATT
T_{O4}	AATGCCCCGTAACAGTGCCCGTATCTCCCTCATTTACCCAGTC
O112	CCGAAATCCGAAAATC
O28	CTCAGAGCCACCACCC

Supplementary Table 23. Other DNA sequences for the origami tiles.

L_1	GAGACAGAGTCCATGACATCGACTAGACGTTGAAGGATACCG ATGAGCACTACCTACCATG
L_2	CGAAGGAGTAGACAATCCAGCTCACTATrAGGAAGAGATGAG ACAGTCACTGAAGATCGACTC
DNAzyme(Zn^{2+})	GGTATCTAGTTGAGCTGTCTAGTC
DNAzyme(Pb^{2+})	TCTCATCTCTGAAGTAGCGCCGCCGTATAGTGAG
H_1	TTAACCTTTTCCTTTAAAAAGGAAAAGGTTAAAATTAAAATGG TAAAAAAGTTGAATTTAAAA
H_2	AAGAAAAGAATTAAGGAATTAAAGTAATGAAAATGAATGGAT GAA AAAATTCATCCATTCATT
E_{his}	GACACCTCTACCAGTGACTTTTTTTCTGCTAACGGGGCTGTGC GGCTAGGAAGTAATACTCTAGTTTTTACAGCCCCGTTAGCAGC AGGTCTCTGCATCAC
K_{his}	GAATGTGATGCAGAGACCTGCTGCTAACGGGGCTGTTTTTCA GGACAACCTGACCATC
B_{his-1}	CATCCGTCGCAGCAGGTCTC
B_{his-2}	TGCATCACATTCAGCCCCTT

B_{his-1}'	CCTGCTGCGACGGATG
B_{his-2}'	AAGGGGCTGAATGTGA
S	FAM-CATTCTTCACTAGAGTATrAGGAGCAGAAAGAAGAATG-BHQ1
G_1	TGGGTGATGGTCGTCTACAGACTGCCAGATTTTGTACGTGTGATACAGCTC
G_2	GTAGACAGGTTGGACTACTTTTGCAGTCTGTAGACGACTGGGTAGGGCGGG
B_{G-1}	TACCCTTCTCTGGCAGTCTG
B_{G-2}	TAGACGACCATCAGTGCTCC
B_{G-1}'	CTGCCAGAGAAGGGTA
B_{G-2}'	GGAGCACTGATGGTCG
H_3	CAGCACACCTAGTTAACCTTTTCCTTTAAAAAGGAAAAGGTTAAAATTAAAATGGTAAAAAAGTTGAATTTAAAACGAGAGGTCATG
H_4	CAGCACACCTAGAAAAGGAAAAGGTTAAAATTAAAATGGTAAAAAAGTTGAATTTAAAAATTTAAATTCAACTTTTCGAGAGGTCATG
H_{3a}'	CATGACCTCTCGTTTTAAATTCAACTTTTTTACCATTTTA
H_{3b}'	ATTTTAACCTTTTCCTTTTTAAAGGAAAAGGTTAACTAGGTGTGCTG
H_{4a}'	ACCATTTTAATTTTAAGGTTTTCCTTTTCTAGGTGTGCTG
H_{4b}'	CATGACCTCTCGAAAAGTTGAATTTAAATTTTTAAATTCAACTTTTTT
G_{1x}	TGGGTCACTGATGGTCGTCTACAGACTGCCAGAGAAGTTTTGTACGTGTGATACAGCTC
B_{GX-1}	GAGCTGTACCCTTCTCTGGCAGTCTG
B_{GX-2}	TAGACGACCATCAGTGCTCCTGACTC
B_{GX-1}'	CTGCCAGAGAAGGGTACAGCTC
B_{GX-2}'	GAGTCAGGAGCACTGATGGTCG
H_{1-C}	GAAGAAAAAACCTTTTCCTTTAAAAAGGAAAAGGTTAAAATTAAAATGGTAAAAAAGTTGAATTTAAAA

Supplementary Table 24. Staple sequences for the rectangle origami.

1	CAAGCCCAATAGGAAC CCATGTACAAACAGTT
2	AATGCCCCGTAACAGT GCCCGTATCTCCCTCA
3	TGCCTTGACTGCCTAT TTCGGAACAGGGATAG
4	GAGCCGCCCCACCACC GGAACCGCGACGGAAA
5	AACCAGAGACCCTCAG AACCGCCAGGGGTCAG
6	TTATTCATAGGGAAGG TAAATATTCATTCAGT
7	CATAACCCGAGGCATA GTAAGAGCTTTTTAAG
8	ATTGAGGGTAAAGGTG AATTATCAATCACCGG

9	AAAAGTAATATCTTAC CGAAGCCCTTCCAGAG
10	GCAATAGCGCAGATAG CCGAACAATTCAACCG
11	CCTAATTTACGCTAAC GAGCGTCTAATCAATA
12	TCTTACCAGCCAGTTA CAAAATAAATGAAATA
13	ATCGGCTGCGAGCATG TAGAAACCTATCATAT
14	CTAATTTATCTTTCCT TATCATTCATCCTGAA
15	GCGTTATAGAAAAAGC CTGTTTAGAAGGCCGG
16	GCTCATTTTCGCATTA AATTTTTGAGCTTAGA
17	AATTACTACAAATTCT TACCAGTAATCCCATC
18	TTAAGACGTTGAAAAC ATAGCGATAACAGTAC
19	TAGAATCCCTGAGAAG AGTCAATAGGAATCAT
20	CTTTTACACAGATGAA TATACAGTAAACAATT
21	TTTAACGTTCGGGAGA AACAATAATTTTCCCT
22	CGACAACTAAGTATTA GACTTTACAATACCGA
23	GGATTTAGCGTATTAA ATCCTTTGTTTTCAGG
24	ACGAACCAAAACATCG CCATTAAATGGTGGTT
25	GAACGTGGCGAGAAAG GAAGGGAACAAACTAT
26	TAGCCCTACCAGCAGA AGATAAAAACATTTGA
27	CGGCCTTGCTGGTAAT ATCCAGAACGAACTGA
28	CTCAGAGCCACCACCC TCATTTTCCTATTATT
29	CTGAAACAGGTAATAA GTTTTAACCCCTCAGA
30	AGTGTACTTGAAAGTA TTAAGAGGCCGCCACC
31	GCCACCACTCTTTTCA TAATCAAACCGTCACC
32	GTTTGCCACCTCAGAG CCGCCACCGATACAGG
33	GACTTGAGAGACAAAA GGGCGACAAGTTACCA
34	AGCGCCAACCATTTGG GAATTAGATTATTAGC
35	GAAGGAAAATAAGAGC AAGAAACAACAGCCAT
36	GCCCAATACCGAGGAA ACGCAATAGGTTTACC
37	ATTATTTAACCCAGCT ACAATTTTCAAGAACG
38	TATTTTGCTCCCAATC CAAATAAGTGAGTTAA
39	GGTATTAAGAACAAGA AAAATAATTAAAGCCA
40	TAAGTCCTACCAAGTA CCGCACTCTTAGTTGC
41	ACGCTCAAAATAAGAA TAAACACCGTGAATTT
42	AGGCGTTACAGTAGGG CTTAATTGACAATAGA
43	ATCAAAATCGTCGCTA TTAATTAACGGATTCG
44	CTGTAAATCATAGGTC TGAGAGACGATAAATA
45	CCTGATTGAAAGAAAT TGCGTAGACCCGAACG
46	ACAGAAATCTTTGAAT ACCAAGTTCCTTGCTT
47	TTATTAATGCCGTCAA TAGATAATCAGAGGTG
48	AGATTAGATTTAAAAG TTTGAGTACACGTAAA
49	AGGCGGTCATTAGTCT TTAATGCGCAATATTA
50	GAATGGCTAGTATTAA CACCGCCTCAACTAAT
51	CCGCCAGCCATTGCAA CAGGAAAAATATTTTT

52	CCCTCAGAACCGCCAC CCTCAGAACTGAGACT
53	CCTCAAGAATACATGG CTTTTGATAGAACCAC
54	TAAGCGTCGAAGGATT AGGATTAGTACCGCCA
55	CACCAGAGTTCGGTCA TAGCCCCCGCCAGCAA
56	TCGGCATTCCGCCGCC AGCATTGACGTTCCAG
57	AATCACCAAATAGAAA ATTCATATATAACGGA
58	TCACAATCGTAGCACC ATTACCATCGTTTTCA
59	ATACCCAAGATAACCC ACAAGAATAAACGATT
60	ATCAGAGAAAGAACTG GCATGATTTTATTTTG
61	TTTTGTTTAAGCCTTA AATCAAGAATCGAGAA
62	AGGTTTTGAACGTCAA AAATGAAAGCGCTAAT
63	CAAGCAAGACGCGCCT GTTTATCAAGAATCGC
64	AATGCAGACCGTTTTT ATTTTCATCTTGCGGG
65	CATATTTAGAAATACC GACCGTGTTACCTTTT
66	AATGGTTTACAACGCC AACATGTAGTTCAGCT
67	TAACCTCCATATGTGA GTGAATAAACAAAATC
68	AAATCAATGGCTTAGG TTGGGTTACTAAATTT
69	GCGCAGAGATATCAAA ATTATTTGACATTATC
70	AACCTACCGCGAATTA TTCATTTCCAGTACAT
71	ATTTTGCGTCTTTAGG AGCACTAAGCAACAGT
72	CTAAAATAGAACAAAG AAACCACCAGGGTTAG
73	GCCACGCTATACGTGG CACAGACAACGCTCAT
74	GCGTAAGAGAGAGCCA GCAGCAAAAAGGTTAT
75	GGAAATACCTACATTT TGACGCTCACCTGAAA
76	TATCACCGTACTCAGG AGGTTTAGCGGGGTTT
77	TGCTCAGTCAGTCTCT GAATTTACCAGGAGGT
78	GGAAAGCGACCAGGCG GATAAGTGAATAGGTG
79	TGAGGCAGGCGTCAGA CTGTAGCGTAGCAAGG
80	TGCCTTTAGTCAGACG ATTGGCCTGCCAGAAT
81	CCGGAAACACACCACG GAATAAGTAAGACTCC
82	ACGCAAAGGTCACCAA TGAAACCAATCAAGTT
83	TTATTACGGTCAGAGG GTAATTGAATAGCAGC
84	TGAACAAACAGTATGT TAGCAAACTAAAAGAA
85	CTTTACAGTTAGCGAA CCTCCCGACGTAGGAA
86	GAGGCGTTAGAGAATA ACATAAAAGAACACCC
87	TCATTACCCGACAATA AACAACATATTTAGGC
88	CCAGACGAGCGCCCAA TAGCAAGCAAGAACGC
89	AGAGGCATAATTTCAT CTTCTGACTATAACTA
90	TTTTAGTTTTTCGAGC CAGTAATAAATTCTGT
91	TATGTAAACCTTTTTT AATGGAAAAATTACCT
92	TTGAATTATGCTGATG CAAATCCACAAATATA
93	GAGCAAAAACTTCTGA ATAATGGAAGAAGGAG
94	TGGATTATGAAGATGA TGAAACAAAATTTCAT

95	CGGAATTATTGAAAGG AATTGAGGTGAAAAAT
96	ATCAACAGTCATCATA TTCCTGATTGATTGTT
97	CTAAAGCAAGATAGAA CCCTTCTGAATCGTCT
98	GCCAACAGTCACCTTG CTGAACCTGTTGGCAA
99	GAAATGGATTATTTAC ATTGGCAGACATTCTG
100	TTTT TATAAGTA TAGCCCGGCCGTCGAGAGGGTTGA
101	TTTT ATAAATCC TCATTAAATGATATTCACAAACAA
102	TTTT AATCAGTA GCGACAGATCGATAGCAGCACCGT
103	TTTT TAAAGGTG GCAACATAGTAGAAAATACATACA
104	TTTT GACGGGAG AATTAACTACAGGGAAGCGCATTA
105	TTTT GCTTATCC GGTATTCTAAATCAGATATAGAAG
106	TTTT CGACAAAA GGTAAAGTAGAGAATATAAAGTAC
107	TTTT CGCGAGAA AACTTTTTATCGCAAGACAAAGAA
108	TTTT ATTAATTA CATTTAACACATCAAGAAAACAAA
109	TTTT TTCATCAA TATAATCCTATCAGATGATGGCAA
110	TTTT AATCAATA TCTGGTCACAAATATCAAACCCTC
111	TTTT ACCAGTAA TAAAAGGGATTCACCA GTCACACG TTTT
112	CCGAAATCCGAAAATC CTGTTTGAAGCCGGAA
113	CCAGCAGGGGCAAAAT CCCTTATAAAGCCGGC
114	GCATAAAGTTCCACAC AACATACGAAGCGCCA
115	GCTCACAATGTAAAGC CTGGGGTGGGTTTGCC
116	TTCGCCATTGCCGGAA ACCAGGCATTAAATCA
117	GCTTCTGGTCAGGCTG CGCAACTGTGTTATCC
118	GTTAAAATTTTAACCA ATAGGAACCCGGCACC
119	AGACAGTCATTCAAAA GGGTGAGAAGCTATAT
120	AGGTAAAGAAATCACC ATCAATATAATATTTT
121	TTTCATTTGGTCAATA ACCTGTTTATATCGCG
122	TCGCAAATGGGGCGCG AGCTGAAATAATGTGT
123	TTTTAATTGCCCGAAA GACTTCAAAACACTAT
124	AAGAGGAACGAGCTTC AAAGCGAAGATACATT
125	GGAATTACTCGTTTAC CAGACGACAAAAGATT
126	GAATAAGGACGTAACA AAGCTGCTCTAAAACA
127	CCAAATCACTTGCCCT GACGAGAACGCCAAAA
128	CTCATCTTGAGGCAAA AGAATACAGTGAATTT
129	AAACGAAATGACCCCC AGCGATTATTCATTAC
130	CTTAAACATCAGCTTG CTTTCGAGCGTAACAC
131	TCGGTTTAGCTTGATA CCGATAGTCCAACCTA
132	TGAGTTTCGTCACCAG TACAAACTTAATTGTA
133	CCCCGATTTAGAGCTT GACGGGGAAATCAAAA
134	GAATAGCCGCAAGCGG TCCACGCTCCTAATGA
135	GAGTTGCACGAGATAG GGTTGAGTAAGGGAGC
136	GTGAGCTAGTTTCCTG TGTGAAATTTGGGAAG
137	TCATAGCTACTCACAT TAATTGCGCCCTGAGA

138	GGCGATCGCACTCCAG CCAGCTTTGCCATCAA
139	GAAGATCGGTGCGGGC CTCTTCGCAATCATGG
140	AAATAATTTTAAATTG TAAACGTTGATATTCA
141	GCAAATATCGCGTCTG GCCTTCCTGGCCTCAG
142	ACCGTTCTAAATGCAA TGCCTGAGAGGTGGCA
143	TATATTTTAGCTGATA AATTAATGTTGTATAA
144	TCAATTCTTTTAGTTT GACCATTACCAGACCG
145	CGAGTAGAACTAATAG TAGTAGCAAACCCTCA
146	GAAGCAAAAAAGCGGA TTGCATCAGATAAAAA
147	TCAGAAGCCTCCAACA GGTCAGGATCTGCGAA
148	CCAAAATATAATGCAG ATACATAAACACCAGA
149	CATTCAACGCGAGAGG CTTTTGCATATTATAG
150	ACGAGTAGTGACAAGA ACCGGATATACCAAGC
151	AGTAATCTTAAATTGG GCTTGAGAGAATACCA
152	GCGAAACATGCCACTA CGAAGGCATGCGCCGA
153	ATACGTAAAAGTACAA CGGAGATTTCATCAAG
154	CAATGACACTCCAAAA GGAGCCTTACAACGCC
155	AAAAAAGGACAACCAT CGCCCACGCGGGTAAA
156	TGTAGCATTCCACAGA CAGCCCTCATCTCCAA
157	GTAAAGCACTAAATCG GAACCCTAGTTGTTCC
158	AGTTTGGAGCCCTTCA CCGCCTGGTTGCGCTC
159	AGCTGATTACAAGAGT CCACTATTGAGGTGCC
160	ACTGCCCGCCGAGCTC GAATTCGTTATTACGC
161	CCCGGGTACTTTCCAG TCGGGAAACGGGCAAC
162	CAGCTGGCGGACGACG ACAGTATCGTAGCCAG
163	GTTTGAGGGAAAGGGG GATGTGCTAGAGGATC
164	CTTTCATCCCCAAAAA CAGGAAGACCGGAGAG
165	AGAAAAGCAACATTAA ATGTGAGCATCTGCCA
166	GGTAGCTAGGATAAAA ATTTTTAGTTAACATC
167	CAACGCAATTTTTGAG AGATCTACTGATAATC
168	CAATAAATACAGTTGA TTCCCAATTTAGAGAG
169	TCCATATACATACAGG CAAGGCAACTTTATTT
170	TACCTTTAAGGTCTTT ACCCTGACAAAGAAGT
171	CAAAAATCATTGCTCC TTTTGATAAGTTTCAT
172	TTTGCCAGATCAGTTG AGATTTAGTGGTTTAA
173	AAAGATTCAGGGGGTA ATAGTAAACCATAAAT
174	TTTCAACTATAGGCTG GCTGACCTTGTATCAT
175	CCAGGCGCTTAATCAT TGTGAATTACAGGTAG
176	CGCCTGATGGAAGTTT CCATTAAACATAACCG
177	TTTCATGAAAATTGTG TCGAAATCTGTACAGA
178	ATATATTCTTTTTTCA CGTTGAAAATAGTTAG
179	AATAATAAGGTCGCTG AGGCTTGCAAAGACTT
180	CGTAACGATCTAAAGT TTTGTCGTGAATTGCG

181	ACCCAAATCAAGTTTT TTGGGGTCAAAGAACG
182	TGGACTCCCTTTTCAC CAGTGAGACCTGTCGT
183	TGGTTTTTAACGTCAA AGGGCGAAGAACCATC
184	GCCAGCTGCCTGCAGG TCGACTCTGCAAGGCG
185	CTTGCATGCATTAATG AATCGGCCCGCCAGGG
186	ATTAAGTTCGCATCGT AACCGTGCGAGTAACA
187	TAGATGGGGGGTAACG CCAGGGTTGTGCCAAG
188	ACCCGTCGTCATATGT ACCCCGGTAAAGGCTA
189	CATGTCAAGATTCTCC GTGGGAACCGTTGGTG
190	TCAGGTCACTTTTGCG GGAGAAGCAGAATTAG
191	CTGTAATATTGCCTGA GAGTCTGGAAAACTAG
192	CAAAATTAAAGTACGG TGTCTGGAAGAGGTCA
193	TGCAACTAAGCAATAA AGCCTCAGTTATGACC
194	TTTTTGCGCAGAAAAC GAGAATGAATGTTTAG
195	AAACAGTTGATGGCTT AGAGCTTATTTAAATA
196	ACTGGATAACGGAACA ACATTATTACCTTATG
197	ACGAACTAGCGTCCAA TACTGCGGAATGCTTT
198	CGATTTTAGAGGACAG ATGAACGGCGCGACCT
199	CTTTGAAAAGAACTGG CTCATTATTTAATAAA
200	GCTCCATGAGAGGCTT TGAGGACTAGGGAGTT
201	ACGGCTACTTACTTAG CCGGAACGCTGACCAA
202	AAAGGCCGAAAGGAAC AACTAAAGCTTTCCAG
203	GAGAATAGCTTTTGCG GGATCGTCGGGTAGCA
204	ACGTTAGTAAATGAAT TTTCTGTAAGCGGAGT
205	TTTT CGATGGCC CACTACGTAAACCGTC TATCAGGG
206	TTTT CGGTTTGC GTATTGGGAACGCGCG GGGAGAGG
207	TTTT TGTAAAAC GACGGCCATTCCCAGT CACGACGT
208	TTTT GTAATGGG ATAGGTCAAAACGGCG GATTGACC
209	TTTT GATGAACG GTAATCGTAGCAAACA AGAGAATC
210	TTTT GGTTGTAC CAAAAACAAGCATAAA GCTAAATC
211	TTTT CTGTAGCT CAACATGTATTGCTGA ATATAATG
212	TTTT CATTGAAT CCCCCTCAAATCGTCA TAAATATT
213	TTTT GGAAGAAA AATCTACGACCAGTCA GGACGTTG
214	TTTT TCATAAGG GAACCGAAAGGCGCAG ACGGTCAA
215	TTTT GACAGCAT CGGAACGAACCCTCAG CAGCGAAA
216	TTTT AACTTTCA ACAGTTTCTGGGATTT TGCTAAAC TTTT
Loop1	AACATCACTTGCCTGAGTAGAAGAACT
Loop2	TGTAGCAATACTTCTTTGATTAGTAAT
Loop3	AGTCTGTCCATCACGCAAATTAACCGT
Loop4	ATAATCAGTGAGGCCACCGAGTAAAAG
Loop5	ACGCCAGAATCCTGAGAAGTGTTTTT
Loop6	TTAAAGGGATTTTAGACAGGAACGGT
Loop7	AGAGCGGGAGCTAAACAGGAGGCCGA

Loop8	TATAACGTGCTTTCCTCGTTAGAATC
Loop9	GTACTATGGTTGCTTTGACGAGCACG
Loop10	GCGCTTAATGCGCCGCTACAGGGCGC

II. Supplementary References

1. Rothemund, P. W. K. Folding DNA to create nanoscale shapes and patterns. *Nature* **440**, 297–302 (2006).
2. Gerling, T., Wagenbauer, K. F., Neuner, A. M. & Dietz, H. Dynamic DNA devices and assemblies formed by shape-complementary, non–base pairing 3D components. *Science* **347**, 1446–1452 (2015).
3. Wagenbauer, K. F., Sigl, C. & Dietz, H. Gigadalton-scale shape-programmable DNA assemblies. *Nature* **552**, 78–83 (2017).
4. Bellot, G., McClintock, M. A., Lin, C. & Shih, W. M. Recovery of intact DNA nanostructures after agarose gel-based separation. *Nat. Methods.* **8,** 192–194 (2011).
